# Silicate-Enabled Mechanochemical Mineralization of Polymeric and Nonpolymeric PFAS into Sodium Fluoride

**DOI:** 10.1021/jacs.6c01470

**Published:** 2026-04-20

**Authors:** Long Yang, Columbus L. Layton, Christopher A. Goult, Zijun Chen, Robert S. Paton, Véronique Gouverneur

**Affiliations:** † Chemistry Research Laboratory, 6396 University of Oxford, Oxford OX1 3TA, U.K.; ‡ Department of Chemistry, 224023 Colorado State University, Fort Collins, Colorado 80528, United States

## Abstract

Per- and polyfluoroalkyl substances (PFAS) are persistent environmental pollutants, some associated with detrimental impacts on human health upon chronic exposure. Many processes have been reported to destroy PFAS, although high-temperature thermal methods such as incineration or pyrolysis are still the most commonly used for bulk waste. Herein, we report a highly effective mechanochemical process designed to destroy polymeric and nonpolymeric PFAS with recovery of fluorine as NaF, a salt endorsed by international organizations for public oral health. The process consists of ball milling PFAS with commercially available sodium silicate, an inexpensive and easy-to-handle reagent found to be superior to sodium phosphate or carbonate. This approach represents a promising step toward remediating the global PFAS crisis with a circular fluorine economy in mind.

## Graphical abstract

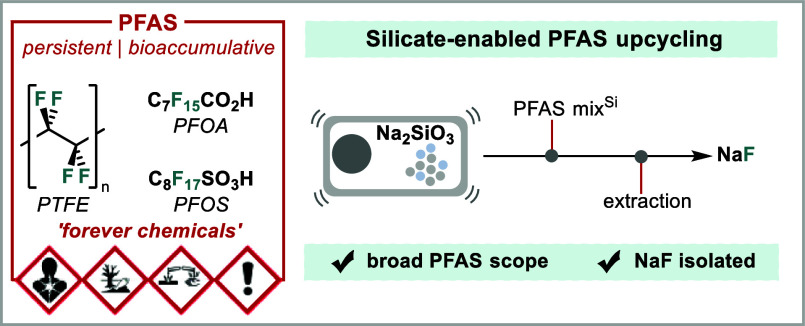

## Introduction

Fluorochemicals play essential roles across a wide range of industries, including materials, agrochemicals, and pharmaceuticals.^1,2^ For synthesis, fluorine is primarily sourced from fluorite (fluorspar, CaF_2_), a mineral currently classified as critical in many countries.^3^ Industrial fluorination processes typically utilize CaF_2_-derived hydrogen fluoride (HF) as the key commodity intermediate,^4^ although strategies bypassing dangerous HF have recently been developed (Figure 1A).^5–10^ At present, global fluorspar reserves are estimated to meet the demand of the steel and fluorochemical industries for only a few more decades.^11^ Although the exploitation of new fluorspar deposits or alternative sources such as fluorapatite may alleviate supply constraints, the escalating demand for fluorinated products, in part driven by rising living standards, will place increasing pressure on existing reserves. This state of play poses significant challenges to the long-term sustainability of fluorine chemistry. Novel strategies are therefore urgently needed to ensure the availability of fluorine-containing products essential to modern life. Such strategies may include resource recovery, recycling of fluorinated waste, and more generally, innovation toward a circular fluorine economy.^12–24^

**Figure 1:**
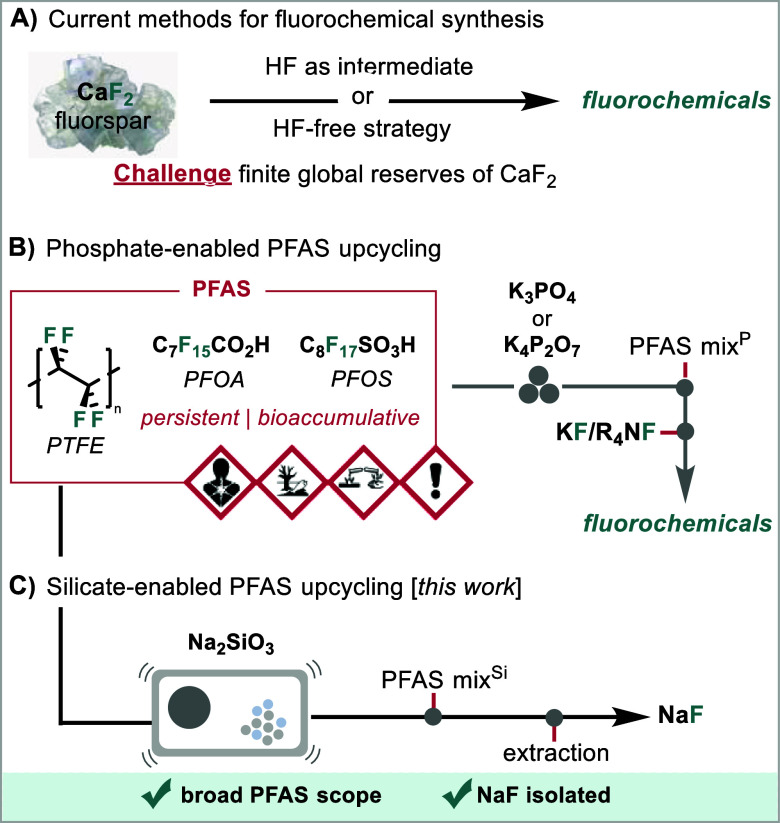
Synthesis of fluorochemicals. (A) Synthesis of fluorochemicals from fluorspar. (B) Phosphate-enabled mechanochemical PFAS destruction coupled with fluoride reuse. (C) Silicate-enabled mechanochemical PFAS destruction for NaF synthesis [this work].

Per- and polyfluoroalkyl substances (PFAS) are synthetic organofluorine compounds widely employed in diverse industrial and consumer applications due to their exceptional thermal and chemical stability, as well as hydro- and lipophobic properties, conferred by the high density of C–F bonds.^25,26^ These characteristics also result in environmental persistence, bioaccumulative potential, and toxicological risks.^27,28^ Over many years, several PFAS degradation methods were developed including advanced oxidation/reduction,^29^ thermal incineration,^30^ and mechanochemical processes^31–41^ among other techniques,^42–51^ but these studies did not demonstrate experimentally fluorine recovery for re-entry in the fluorochemical sector.

In 2025, we reported a highly effective phosphate-enabled mechanochemical process coupling PFAS destruction with fluorine recovery as reagents such as KF, that are suitable for S–F and C–F bond forming processes (Figure 1B).^12^ This chemistry stood out because it is compatible with all PFAS classes, and it provides a path toward a circular fluorine economy. With this method, an effective strategy was implemented to recycle the phosphate salts because of their strategic importance in food security and clean energy. The process was less effective for fluorine recovery as NaF, a limitation considering the global use of this salt in water fluoridation. Our next objective was therefore to invent a technology equally general in terms of PFAS range for the formation and isolation of NaF, ideally using an easy-to-handle activator other than a phosphate salt in order to avoid the necessity of a recycling step. Methods that mineralize PFAS into NaF are known but present limitations. Shibata^14^ and Armstrong^23^ described the use of sodium metal dispersion as a reductant to convert selected PFAS to NaF. These methods are elegant, but raise safety concerns due to the violent reaction of sodium upon contact with water or air. Departing from mechanochemistry, NaOH was disclosed for the destruction of functionalized PFAS,^32,44–46^ but these reports do not demonstrate fluoride recovery as isolated NaF.

## Results and Discussion

In preliminary experiments, ball milling PTFE with sodium phosphate revealed significantly reduced reactivity compared to potassium phosphate (Figure 2A, entries 1 and 2). In our previous work,^12^ potassium oxyanions other than phosphate, were considered but found to be less efficient. Among the activators that we considered, potassium carbonate ranked as second best after phosphates releasing 75% of the fluorine content of polytetrafluoroethylene (PTFE) as fluoride, a process accompanied by substantial CO_2_ emission.^12^ A preliminary experiment performed with Na_2_CO_3_ revealed 27% release of fluoride from PTFE (Table S1), prompting the search for a better activator. We hypothesized that sodium metasilicate Na_2_SiO_3_ may be better suited to destroy PFAS while simultaneously enabling recovery of NaF upon PFAS mineralization. The generation of SiO_2_ as a potential byproduct upon PFAS mineralization may provide a thermodynamic advantage versus Na_2_CO_3_,^52^ and will not pose immediate recycling challenges that we encountered with phosphate salts (Figure 1). We were encouraged by the elegant work from Weber^36–39^ and Sperry^40,41^ demonstrating that SiO_2_ promotes the mineralization of PFAS such as PFOA and PFOS under mechanical conditions. The authors propose a process based on homolytic Si–O bond cleavage and the formation of silicon- and oxygen-centered radicals with the resulting products identified as a neutral fluorine complex, an [Si–F] species, which is expected in the absence of metal counterions.^41,53^ Complementing this methodology, a silicate-enabled mechanochemical process may directly produce NaF from a broad range of PFAS including highly persistent fluoroplastics.

**Figure 2:**
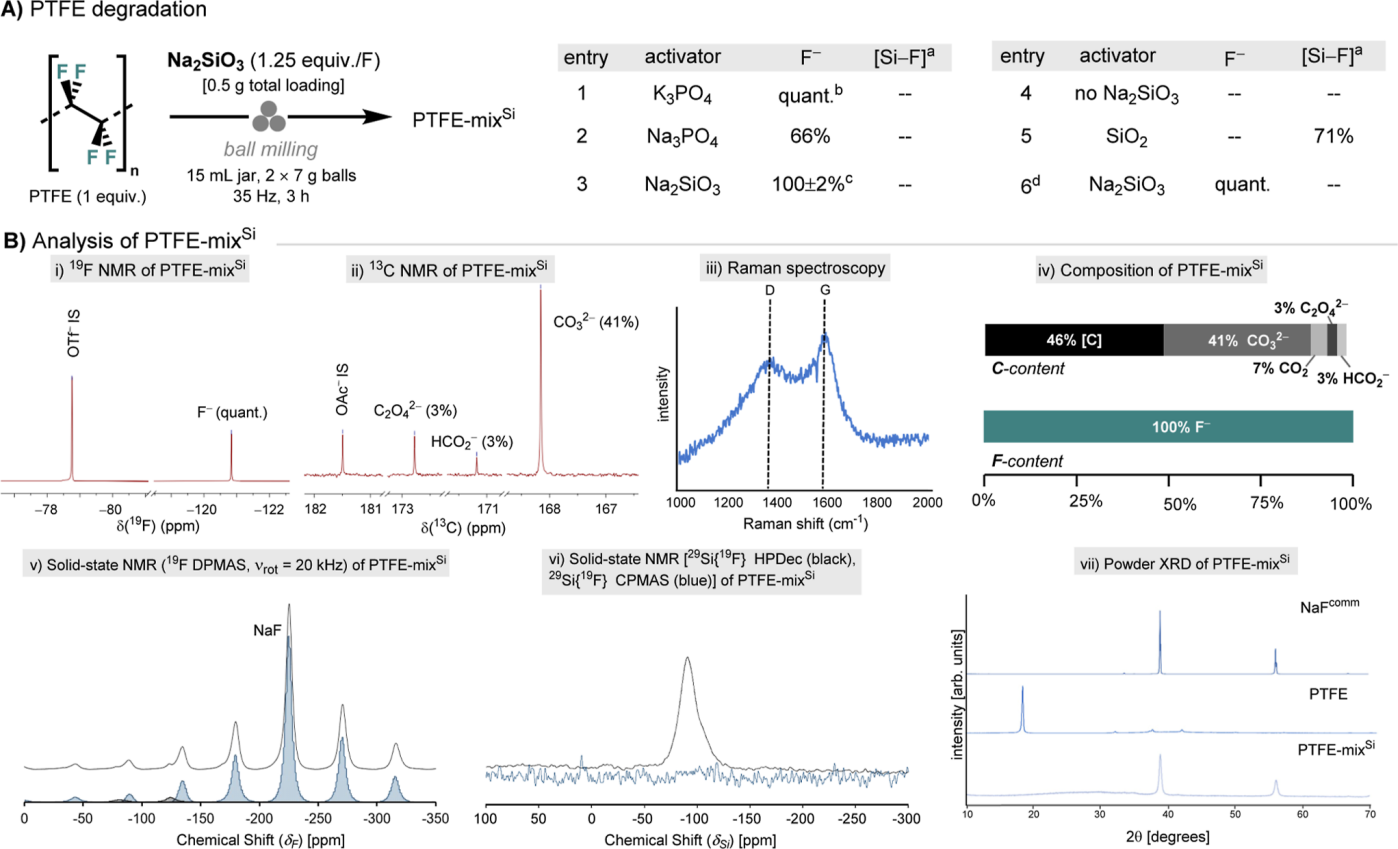
PFAS degradation. (A) PTFE degradation with different activators. Ball milling conditions: PFAS (1 equiv) milled with activator (1.25 equiv/F) in a 15 mL stainless-steel milling jar, two chrome steel balls (2 × 7 g) at 35 Hz for 3 h; the yield of fluoride (F^–^) release was determined by quantitative ^19^F NMR spectroscopy (10% D_2_O in H_2_O, NaOTf as an internal standard) using an aliquot of PTFE-mix^Si^; ^a^The yield of [Si–F] species was determined by quantitative ^19^F NMR spectroscopy (10% D_2_O in H_2_O, NaOTf as an internal standard) after the treating an aliquot of PTFE-mix^Si^ with KOH (0.1 mL, 10 M). ^b^F^–^/PO_3_F^2–^ (5.6:1). ^c^Recorded in triplicate, value reported as the mean with one standard deviation. ^d^15 mL zirconia jar and 2 × 6 g zirconia balls were used instead of 15 mL stainless-steel jar and 2 × 7 g chrome steel. (B) Analysis of PTFE-mix^Si^. (i) Quantitative ^19^F NMR spectroscopy (10% D_2_O in H_2_O, NaOTf as an internal standard) using an aliquot of PTFE-mix^Si^, indicating quantitative fluoride release. (ii) Quantitative ^13^C NMR spectroscopy (10% D_2_O in H_2_O, KOAc as an internal standard) using an aliquot of PTFE-mix^Si^, indicating CO_3_^2–^ (δ_C_ = 168.1) along with trace amounts of C_2_O_4_^2–^ (δ_C_ = 172.8) and HCO_2_^–^ (δ_C_ = 171.2). (iii) Raman spectroscopy of water-insoluble black residue after aqueous extraction, indicating D (disordered carbon) and G (graphitic carbon) bands. (iv) Carbon and fluorine content of PTFE-mix^Si^ based on the data sets (i–iii), the remaining carbon [C] is water insoluble and assumed to be mineralized. (v) ^19^F DPMAS of PTFE-mix^Si^. (vi) ^29^Si­{^19^F} HPDec (black) and ^29^Si­{^19^F} CPMAS (blue) of PTFE-mix^Si^. (vii) Powder X-ray diffractogram (XRD) of NaF (commercial), PTFE (commercial) and PTFE-mix^Si^ (top to bottom). IS = internal standard, quant. = quantitative, [Si–F] = silicon–fluorine species, NaF^comm^ = commercial NaF.

The reactivity of commercially available and inexpensive Na_2_SiO_3_ (1.25 equiv/F) with PTFE (1.0 equiv) was investigated applying vibrational ball milling (35 Hz, 3 h) within a steel milling jar equipped with a rubber sealing ring. An aliquot of the resulting solid material (PTFE-mix^Si^) was analyzed by ^19^F qNMR spectroscopy (10% D_2_O in H_2_O), that revealed a resonance at −120.8 ppm ascribed to F^–^ being released quantitatively (Figure 2A, entry 3). Control experiments involving mechanical milling of PTFE in the absence of Na_2_SiO_3_ showed no detectable fluoride release, whereas the use of SiO_2_ (1.25 equiv/F) instead of Na_2_SiO_3_ resulted in 71% fluoride release, which was determined upon subsequent base hydrolysis of the resulting [Si–F] species (Figure 2A, entries 4, 5). Performing the reaction with Na_2_SiO_3_ in a zirconia jar equipped with zirconia coated balls gave quantitative fluoride release (Figure 2A entry 6), confirming that PTFE degradation is induced by the silicate salt under the reaction conditions and is not linked to metal leaching from steel components.

*Ex situ* time-course analysis of PTFE-mix^Si^ indicated that Na_2_SiF_6_ or other [Si–F] fluorine species were not detected upon milling with Na_2_SiO_3_ (Figure S2). Further analysis was carried out to elucidate the composition of PTFE-mix^Si^. Quantitative ^13^C­{^1^H} NMR spectroscopy gave insight into the fate of the carbon skeleton (Figure 2B). The water-soluble fraction of PTFE-mix^Si^ contained CO_3_^2–^ (δ_C_ = 168.1 ppm, 41% *C*_tot_) along with trace amounts of C_2_O_4_^2–^ (δ_C_ = 172.8 ppm, 3% *C*_tot_) and HCO_2_^–^ (δ_C_ = 171.2 ppm, 3% *C*_tot_). Gas capture analysis revealed that CO_2_ is produced (7% *C*_tot_, Figure S6). The remaining carbon (46%) is a black solid insoluble in water. Raman spectroscopy of this insoluble solid isolated from PTFE–mix^Si^ revealed bands at 1355 and 1579 cm^–1^, corresponding to the disordered (D) and graphitic (G) bands of carbon (Figure 2B­(iii)).^54^ Further analysis of this insoluble solid by X-ray photoelectron spectroscopy revealed (C)–C, (Si)–O, (C)–O, (C)–F (trace) peaks. Solid-state ^19^F NMR spectroscopy of PTFE-mix^Si^ revealed that fluorine was near-quantitatively mineralized as NaF (δ_F_ = −225.4 ppm, 97%) with trace amount of residual PTFE (δ_F_ = −123.4 ppm, 3%); no [Si–F] species were observed by solid-state ^29^Si­{^19^F} NMR (Figure 2B­(v,vi)). The FTIR spectrum of PTFE-mix^Si^ confirmed the absence of peak at 714 cm^–1^ characteristic of SiF_6_^2–^ (Figure S12). The formation of NaF was unambiguously confirmed by powder X-ray diffraction (XRD) analysis of PTFE-mix^Si^ (Figure 2B­(vii)). Following experimental determination of the components of PTFE-mix^Si^ (Figure 2B), the standard reaction enthalpy change (Δ_r_ *H*°) for the idealized process affording PTFE-mix^Si^ from PTFE and Na_2_SiO_3_ was calculated to be −158.5 kcal/mol (see Supporting Information, Section 7 Thermochemistry). Such silicate-mediated destruction of PTFE is therefore thermodynamically favorable.

Preliminary computational studies provided further mechanistic insights of this silicate-enabled destruction process. Density functional theory (DFT) calculations were performed on a putative nucleophilic substitution reaction between sodium silicate and the model PFAS perfluorobutane in gas phase (Figure S32). The solid state of sodium silicate features SiO_4_ tetrahedra bridged by oxygen atoms.^55^ Thus, the cyclic silicate trimer Na_6_(SiO_3_)_3_ was selected as the truncated sodium silicate nucleophile because this species maintains the tetrahedral geometry around silicon. The computed activation barrier was found to be 42.2 kcal/mol. Theoretical models of the mechanical effects of ball-milling estimate that reductions in bimolecular activation energies of up to −16.5 kcal/mol can arise for some directions of impact.^56^ As a comparison, the activation barrier increased when the nucleophile was replaced by a cluster containing three units of sodium phosphate (Δ*G* ^‡^ = 48.7 kcal/mol), a result correlating with the experimental observation that sodium silicate is a superior activator. Of note, the weakest homolytic bond dissociation of perfluorobutane is 103 kcal/mol and the average C–C bond energy in PTFE is 90 kcal/mol. Under high-energy milling conditions, the mechanistic pathway may therefore involve both nucleophilic and radical processes.

Next, we examined the generality of this process with a range of representative PFAS (Figure 3A). The methodology was successfully extended to both polymeric (1–7) and nonpolymeric PFAS (8–14), achieving up to quantitative yields of fluoride mineralization. The fluoropolymers included in our study are polyvinylidene fluoride (PVDF, 2), polychlorotrifluoroethylene (PCTFE, 3), polyvinyl fluoride film (PVF, 4), ethylene tetrafluoroethylene (ETFE, 5), poly­(vinylidene fluoride-*co*-hexafluoropropylene) (PVDF-HFP, 6), and perfluoroalkoxy alkane (PFA) tubing (7). The method was equally effective for nonpolymeric PFAS, including perfluorooctanoic acid (PFOA, 8), potassium perfluorohexanesulfonate (KPFHxS, 9), tetraethylammonium perfluorooctanesulfonate (PFOSNEt_4_, 10), 6:2 fluorotelomer phosphonic acid (6:2 FTPA, 11), 6:2 fluorotelomer alcohol (6:2 FTOH, 12), 8:2 fluorotelomer alcohol (8:2 FTOH, 13) and sodium trifluoroacetate (CF_3_CO_2_Na, 14). The method was also successfully applied to real-world consumer products, including PTFE seals (15), PTFE tapes (16), ETFE wires (17), fluorinated ethylene propylene (FEP) tubing (18), and PVDF fittings (19). All materials were decomposed effectively under identical reaction conditions, with near-quantitative mineralization into sodium fluoride. Additionally, samples of PFAS adsorbed on powdered activated carbon (PAC) or granular activated carbon (GAC) were also completely destroyed, indicating utility in the treatment of PFAS-contaminated water matrices.

**Figure 3:**
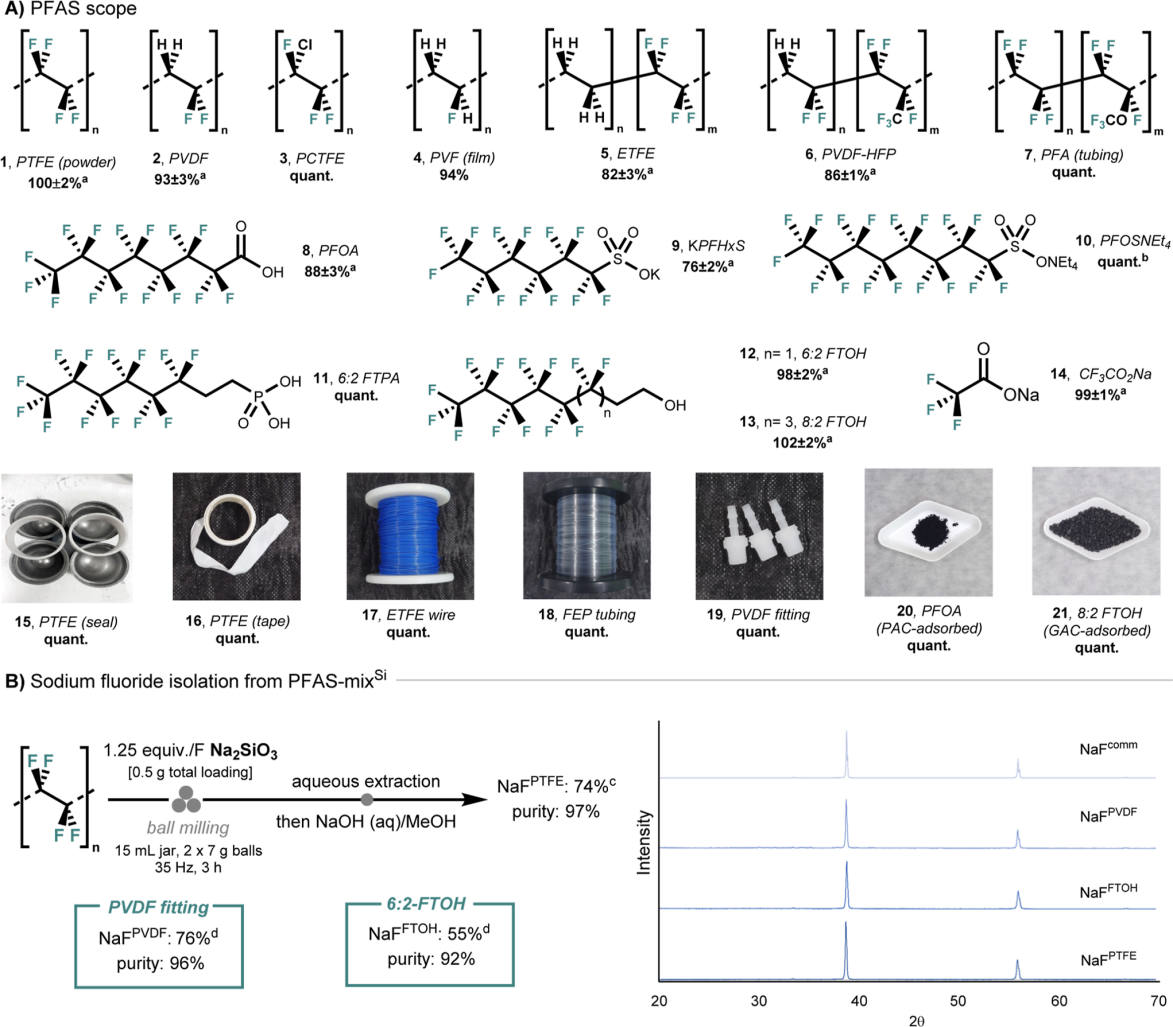
PFAS scope and NaF synthesis. (A) PFAS degradation scope with Na_2_SiO_3_. Ball milling conditions: PFAS (1 equiv) milled with Na_2_SiO_3_ (1.25 equiv/F) in a 15 mL stainless-steel milling jar, two chrome steel balls (2 × 7 g) at 35 Hz for 3 h. The yields of released fluoride were determined by ^19^F qNMR spectroscopy (10% D_2_O in H_2_O, NaOTf as an internal standard). (B) NaF isolation from PTFE-mix^Si^. ^a^Recorded in triplicate, value reported as the mean with one standard deviation. ^b^Na_2_SiO_3_ (2 equiv/F), milling 6 h. ^c^11 jars of PFAS-mix^Si^ were used to isolate NaF^PFAS^ from the procedure in (B). ^d^3 jars of PFAS-mix^Si^ were used to isolate NaF^PFAS^ from the procedure in (B). Purities were determined by ^19^F qNMR spectroscopy (10% D_2_O in H_2_O, NaOTf as an internal standard). NaF^comm^ = commercial NaF, NaF^PVDF^ = NaF synthesized from PVDF fittings, NaF^FTOH^ = NaF synthesized from 6:2-FTOH, NaF^PTFE^ = NaF synthesized from PTFE.

With a general method to destroy PFAS in hand, we developed an efficient route to isolate NaF from PTFE-mix^Si^ (Figure 3B). Extraction of PTFE-mix^Si^ with H_2_O followed by aqueous NaOH^57^ (common ion effect) facilitated the isolation of NaF^PTFE^ in 74% yield and 97% purity. The protocol was further extended to PVDF fittings (NaF^PVDF^: 76% yield, 96% purity), and to nonpolymeric 6:2-FTOH (NaF^FTOH^: 55% yield, 92% purity). Samples of NaF were characterized by powder X-ray diffraction (Figure 3B), and their purity was determined by ^19^F qNMR, with elemental analysis for NaF^PTFE^ (Figures S15, S18, and S21, and Table S3).

## Conclusion

In summary, we have developed a novel silicate-enabled mechanochemical process for PFAS destruction coupled with fluoride recovery as sodium fluoride. Given the global crisis of PFAS waste, and the finite nature of fluorspar, our process offers dual benefits as it enables highly effective PFAS mineralization, and it supports a circular fluorine economy for the fluorochemical industry, illustrated here with fluorine recovery as sodium fluoride.

## Supplementary Material



## Notes

Materials and methods, optimization studies, control and mechanistic experiments, detailed experimental procedures, and characterization data (PXRD traces, NMR spectra) (PDF)

## References

[1] Groult, H. ; Leroux, F. ; Tressaud, A. Modern Synthesis Processes and Reactivity of Fluorinated Compounds: Progress in Fluorine Science; Elsevier, 2016.

[2] Britton R., Gouverneur V., Lin J.-H., Meanwell M., Ni C., Pupo G., Xiao J.-C., Hu J. (2021). Contemporary synthetic
strategies in organofluorine chemistry. Nat.
Rev. Methods Primers.

[3] Milewski, A. Fluorspar: the EV critical mineral no one has heard of, Oregon Group, https://theoregongroup.com/investment-insights/fluorspar-the-ev-critical-mineral-no-one-has-heard-of/ (accessed April 08, 2026).

[4] Harsanyi A., Sandford G. (2015). Organofluorine chemistry:
applications, sources and
sustainability. Green Chem..

[5] Patel C., André-Joyaux E., Leitch J. A., de Irujo-Labalde X. M., Ibba F., Struijs J., Ellwanger M. A., Paton R., Browne D. L., Pupo G., Aldridge S., Hayward M. A., Gouverneur V. (2023). Fluorochemicals from fluorspar via
a phosphate-enabled mechanochemical process that bypasses HF. Science.

[6] Klose I., Patel C., Mondal A., Schwarz A., Pupo G., Gouverneur V. (2024). Fluorspar
to fluorochemicals upon low-temperature activation
in water. Nature.

[7] Schlatzer T., Goult C. A., Hayward M. A., Gouverneur V. (2025). One-Step HF-Free
Synthesis of Alkali Metal Fluorides from Fluorspar. J. Am. Chem. Soc..

[8] Liu J., Cai Y., Xiao C., Zhang H., Lv F., Luo C., Hu Z., Cao Y., Cao B., Yu L. (2019). Synthesis of LiPF_6_ Using CaF_2_ as the Fluorinating Agent Directly:
An Advanced Industrial Production Process Fully Harmonious to the
Environments. Ind. Eng. Chem. Res..

[9] Liu J., Cai Y., Pang H., Cao B., Luo C., Hu Z., Xiao C., Zhang H., Lv F., Cao Y., Yu L. (2022). Chloro-free synthesis of LiPF_6_ using the fluorine-oxygen
exchange technique. Chin. Chem. Lett..

[10] Tarbutton G., Egan E. P., Frary S. G. (1941). Phosphorus-Halogen
Compounds from
Phosphorus Pentoxide and Halides. Properties of Phosphorus Trifluoride
and Phosphorus Oxyfluoride. J. Am. Chem. Soc..

[11] McRae, M. E. Fluorspar Statistics and Information, U.S. Geological Survey. 2024, https://www.usgs.gov/centers/national-minerals-information-center/fluorspar-statistics-and-information (accessed May 27, 2025).

[12] Yang L., Chen Z., Goult C. A., Schlatzer T., Paton R. S., Gouverneur V. (2025). Phosphate-enabled
mechanochemical
PFAS destruction for fluoride reuse. Nature.

[13] Hattori M., Saha D., Bacho M. Z., Shibata N. (2025). Mechanochemical pathway
for converting fluoropolymers to fluorochemicals. Nat. Chem..

[14] Araki T., Ota H., Murata Y., Sumii Y., Hamaura J., Adachi H., Kagawa T., Hori H., Escorihuela J., Shibata N. (2025). Room-temperature defluorination
of PTFE and PFAS via
sodium dispersion. Nat. Commun..

[15] Hattori M., Kiyono T., Zhao Z., Higashi M., Fujishiro M., Kishikawa Y., Escorihuela J., Shibata N. (2025). Upcycling of PTFE and
PVDF to fluorochemicals through mechanochemical process. Nat. Commun..

[16] Bui M., Heinekamp C., Fuhry E., Weidner S., Radnik J., Ahrens M., Scheurell K., Balasubramanian K., Emmerling F., Braun T. (2025). Lewis-acid induced mechanochemical
degradation of polyvinylidene fluoride: transformation into valuable
products. Chem. Sci..

[17] Long H., Kirby G., Ackermann L. (2026). Single-Pot Mechanochemically-Enabled
Fluorine Atom Closed-Loop Economy Using PFASs as Fluorinating Agents. Nat. Commun..

[18] Farley S. E. S., Crimmin M. R. (2026). Synthetic Methodologies
for the Chemical Recycling
of Fluorocarbons. Nat. Chem. Rev..

[19] Sheldon D. J., Parr J. M., Crimmin M. R. (2023). Room Temperature
Defluorination of
Poly­(tetrafluoroethylene) by a Magnesium Reagent. J. Am. Chem. Soc..

[20] Yang W., White A. J. P., Crimmin M. R. (2025). Boron,
Aluminum, and Gallium Fluorides
as Catalysts for the Defluorofunctionalization of Electron-Deficient
Arenes: The Role of NaBAr^F^
_4_ Promoters. Inorg. Chem..

[21] Sheldon D. J., Parr J. M., Crimmin M. R. (2024). Defluorination
of HFCs by a magnesium
reagent. Dalton Trans..

[22] Patrick S. L., Bull J. A., Miller P. W., Crimmin M. R. (2024). A Continuous Flow
Process for the Defluorosilylation of HFC-23 and HFO-1234yf. Org. Lett..

[23] Lowe M. E., Gallant B. M., Davison N., Hopkinson M. N., Kubicki D. J., Lu E., Armstrong R. J. (2025). A Reductive
Mechanochemical Approach Enabling Direct Upcycling of Fluoride from
Polytetrafluoroethylene (PTFE) into Fine Chemicals. J. Am. Chem. Soc..

[24] Morita Y., Saito Y., Kumagai S., Kameda T., Shiratori T., Yoshioka T. (2024). Fluorine recovery through alkaline defluorination of
polyvinylidene fluoride. J. Mater. Cycles Waste
Manage..

[25] Leung S. C. E., Wanninayake D., Chen D., Nguyen N.-T., Li Q. (2023). Physicochemical
properties and interactions of perfluoroalkyl substances (PFAS) -
Challenges and opportunities in sensing and remediation. Sci. Total Environ..

[26] Gluge J., Scheringer M., Cousins I. T., DeWitt J. C., Goldenman G., Herzke D., Lohmann R., Ng C. A., Trier X., Wang Z. (2020). An overview of the uses of per- and polyfluoroalkyl substances (PFAS). Environ. Sci.: Processes Impacts.

[27] Hoskins T. D., Flynn R. W., Coogan G. S. M., Catlin A. C., de Perre C., Modiri Gharehveran M., Choi Y. J., Lee L. S., Hoverman J. T., Sepulveda M. S. (2023). Chronic Exposure to a PFAS Mixture Resembling AFFF-Impacted
Surface Water Decreases Body Size in Northern Leopard Frogs (Rana
pipiens). Environ. Sci. Technol..

[28] Mikkonen A. T., Martin J., Upton R. N., Barker A. O., Brumley C. M., Taylor M. P., Mackenzie L., Roberts M. S. (2023). Spatio-temporal
trends in livestock exposure to per- and polyfluoroalkyl substances
(PFAS) inform risk assessment and management measures. Environ. Res..

[29] Khan Q., Sayed M., Khan J. A., Rehman F., Noreen S., Sohni S., Gul I. (2024). Advanced oxidation/reduction
processes
(AO/RPs) for wastewater treatment, current challenges, and future
perspectives: a review. Environ. Sci. Pollut.
Res..

[30] United States Environmental Protection Agency . Per- and Polyfluoroalkyl Substances (PFAS): Incineration to Manage PFAS Waste Streams. 2020, https://www.epa.gov/sites/default/files/2019-09/documents/technical_brief_pfas_incineration_ioaa_approved_final_july_2019.pdf (accessed April 8, 2026).

[31] Zhang K., Huang J., Yu G., Zhang Q., Deng S., Wang B. (2013). Destruction of Perfluorooctane
Sulfonate (PFOS) and Perfluorooctanoic
Acid (PFOA) by Ball Milling. Environ. Sci. Technol..

[32] Zhang Q., Lu J., Saito F., Baron M. (2001). Mechanochemical solid-phase reaction
between polyvinylidene fluoride and sodium hydroxide. J. Appl. Polym. Sci..

[33] Ateia M., Skala L. P., Yang A., Dichtel W. R. (2021). Product analysis
and insight into the mechanochemical destruction of anionic PFAS with
potassium hydroxide. J. Hazard. Mater. Adv..

[34] Yang N., Yang S., Ma Q., Beltran C., Guan Y., Morsey M., Brown E., Fernando S., Holsen T. M., Zhang W., Yang Y. (2023). Solvent-Free Nonthermal
Destruction
of PFAS Chemicals and PFAS in Sediment by Piezoelectric Ball Milling. Environ. Sci. Technol. Lett..

[35] Cagnetta G., Zhang Q., Huang J., Lu M., Wang B., Wang Y., Deng S., Yu G. (2017). Mechanochemical
destruction
of perfluorinated pollutants and mechanosynthesis of lanthanum oxyfluoride:
A Waste-to-Materials process. Chem. Eng. J..

[36] Turner L. P., Kueper B. H., Jaansalu K. M., Patch D. J., Battye N., El-Sharnouby O., Mumford K. G., Weber K. P. (2021). Mechanochemical
remediation of perfluorooctanesulfonic acid (PFOS) and perfluorooctanoic
acid (PFOA) amended sand and aqueous film-forming foam (AFFF) impacted
soil by planetary ball milling. Sci. Total Environ..

[37] Battye N. J., Patch D. J., Roberts D. M. D., O’Connor N. M., Turner L. P., Kueper B. H., Hulley M. E., Weber K. P. (2022). Use of
a horizontal ball mill to remediate per- and polyfluoroalkyl substances
in soil. Sci. Total Environ..

[38] Turner L. P., Kueper B. H., Patch D. J., Weber K. P. (2023). Elucidating the
relationship between PFOA and PFOS destruction, particle size and
electron generation in amended media commonly found in soils. Sci. Total Environ..

[39] Battye N., Patch D., Koch I., Monteith R., Roberts D., O’Connor N., Kueper B., Hulley M., Weber K. (2024). Mechanochemical
degradation of per- and polyfluoroalkyl substances in soil using an
industrial-scale horizontal ball mill with comparisons of key operational
metrics. Sci. Total Environ..

[40] Gobindlal K., Shields E., Whitehill A., Weber C. C., Sperry J. (2023). Mechanochemical
destruction of per- and polyfluoroalkyl substances in aqueous film-forming
foams and contaminated soil. Environ. Sci.:
Adv..

[41] Gobindlal K., Zujovic Z., Jaine J., Weber C. C., Sperry J. (2023). Solvent-Free,
Ambient Temperature and Pressure Destruction of Perfluorosulfonic
Acids under Mechanochemical Conditions: Degradation Intermediates
and Fluorine Fate. Environ. Sci. Technol..

[42] Fang J., Li S., Gu T., Liu A., Qiu R., Zhang W.-X. (2024). Treatment
of per- and polyfluoroalkyl substances (PFAS): A review of transformation
technologies and mechanisms. J. Environ. Chem.
Eng..

[43] Berhanu A., Mutanda I., Taolin J., Qaria M. A., Yang B., Zhu D. (2023). A review of microbial
degradation of per- and polyfluoroalkyl substances
(PFAS): Biotransformation routes and enzymes. Sci. Total Environ..

[44] Trang B., Li Y., Xue X.-S., Ateia M., Houk K. N., Dichtel W. R. (2022). Low-temperature
mineralization of perfluorocarboxylic acids. Science.

[45] Monsky R. J., Li Y., Houk K. N., Dichtel W. R. (2024). Low-Temperature Mineralization of
Fluorotelomers with Diverse Polar Head Groups. J. Am. Chem. Soc..

[46] Yanagihara N., Katoh T. (2022). Mineralization of poly­(tetrafluoroethylene)
and other fluoropolymers
using molten sodium hydroxide. Green Chem..

[47] Luo S., Xie Z., Xiong X., Bai L., Wang H., Wei Z. (2025). Activating
PFAS to Unlock Efficient Defluorination. Environ.
Sci. Technol..

[48] Liu X., Sau A., Green A. R., Popescu M. V., Pompetti N. F., Li Y., Zhao Y., Paton R. S., Damrauer N. H., Miyake G. M. (2025). Photocatalytic
C-F bond activation in small molecules and polyfluoroalkyl substances. Nature.

[49] Zhang H., Chen J.-X., Qu J.-P., Kang Y.-B. (2024). Photocatalytic low-temperature
defluorination of PFASs. Nature.

[50] Gao J., Liu Z., Chen Z., Rao D., Che S., Gu C., Men Y., Huang J., Liu J. (2023). Photochemical degradation pathways
and near-complete defluorination of chlorinated polyfluoroalkyl substances. Nat. Water.

[51] Guan Y., Liu Z., Yang N., Yang S., Quispe-Cardenas L. E., Liu J., Yang Y. (2024). Near-complete
destruction of PFAS in aqueous film-forming
foam by integrated photo-electrochemical processes. Nat. Water.

[52] 9 - Silicon. In Chemistry of the Elements (Second Edition), Greenwood, N. N ; Earnshaw, A , Eds.; Butterworth-Heinemann, 1997, pp 328−366.

[53] Gobindlal K., Zujovic Z., Yadav P., Sperry J., Weber C. C. (2021). The Mechanism
of Surface-Radical Generation and Amorphization of Crystalline Quartz
Sand upon Mechanochemical Grinding. J. Phys.
Chem. C.

[54] Saravanan M., Ganesan M., Ambalavanan S. (2014). An in situ
generated carbon as integrated
conductive additive for hierarchical negative plate of lead-acid battery. J. Power Sources.

[55] Grund A., Pizy M. (1952). Structure cristalline
du metasilicate de sodium anhydre, Na_2_SiO_3_. Acta Crystallogr..

[56] De
Armas R., Temprado M., Frutos L. M. (2025). Computational Model
to Predict Reactivity under Ball-Milling Conditions. J. Chem. Theory Comput..

[57] Wang X., Ge Q. (2018). Separation and recovery of NaF from
fluorine containing solution
by the common ion effect of Na^+^. Heliyon.

