# Supplementary material for: Silicate-Enabled Mechanochemical Mineralization of Polymeric and Nonpolymeric PFAS into Sodium Fluoride
Source: J Am Chem Soc. 2026 Apr 20;148(17):17977–82. doi: 10.1021/jacs.6c01470 (PMC13154186; doi:10.1021/jacs.6c01470)

Supporting Information

Silicate-Enabled Mechanochemical Mineralization of Polymeric and Nonpolymeric PFAS into Sodium Fluoride

Long Yang,[1] Columbus L. Layton,[1] Christopher A. Goult,[1] Zijun Chen,[1] Robert S. Paton,[2] Véronique Gouverneur*[1]

[1] Chemistry Research Laboratory, University of Oxford, Oxford OX1 3TA, U.K.

[2] Department of Chemistry, Colorado State University, Fort Collins, Colorado 80528, United States

Correspondence to: veronique.gouverneur@chem.ox.ac.uk

Contents

1. Materials and methods 3
2. Optimisation studies on the degradation of PTFE 4
2.1 Activator screening 4
2.2 Optimisation of activator stoichiometry 4
2.3 Mechanochemical PTFE Activation Time-Course 5
2.4 Effect of Jar Loading on Fluoride Release 6
3. Composition of PTFE-mix^{Si} 7
3.1 Solution-Phase qNMR Spectroscopy 7
3.2 Solid-State Analysis of Carbon Black 9
3.3 Powder X-ray Diffraction of PTFE-mix^{Si} 10
3.4 FTIR Spectra of PTFE-mix^{Si} 12
3.5 Solid-state NMR spectroscopy of PTFE-mix^{Si} 12
4. Isolation of Alkali Metal Fluorides 13
4.1 Isolation of NaF from PFAS-mix^{Si} 13
4.2 Scaled-Up Isolation of NaF from PFAS-mix^{Si} 19
4.3. Elemental analysis of NaF^{PTFE} 21
4.4 Isolation of KF from PFAS-mix^{Si} 21
4.5 Synthesis of Tosyl Fluoride using NaF derived from PFAS-mix^{Si} 23
5. Photographic guide for experimental setup (ball milling) 25
6. Mechanochemical destruction of various PFAS 26
6.1. General procedures 26
6.2. PFAS scope 26
7. Thermochemistry 31
8. Computational Studies 31
9. References 36
10. PFAS-mix NMR characterization 38

1. Materials and methods

Unless otherwise stated, all reagents were purchased from commercial suppliers (Sigma-Aldrich, Alfa Aesar, Fluorochem, Apollo Scientific and Fisher Chemicals), used without further purification and stored under ambient conditions. Solvents were purchased from commercial suppliers and used as provided without further purification.

^{1}H NMR, ^{19}F NMR, and ^{13}C NMR spectra were recorded on either Bruker AVIIIHD 400 (Operating frequencies; ^{1}H 400.130 MHz, ^{19}F 376.498 MHz, ^{13}C 100.613 MHz), AVIIIHD 500 (Operating frequencies; ^{1}H 500.130 MHz, ^{19}F 470.592 MHz, ^{13}C 125.758 MHz), or AVIII 500 (Operating frequencies; ^{1}H 500.130 MHz, ^{19}F 470.592 MHz, ^{13}C 125.758 MHz) spectrometers. ^{1}H NMR, ^{13}C NMR, and ^{19}F NMR spectral data are reported as chemical shifts (δ) in parts per million (ppm) relative to the solvent peak using the Bruker internal referencing procedure. NMR spectra were processed with MestReNova 14.1.2 or Topspin 3.5 or 4.0. Quantitative ^{19}F NMR spectra were recorded using a 10 second recycle delay, using sodium triflate as an internal standard. Quantitative ^{13}C{^{1}H} NMR spectra were recorded on a Bruker AVIIIHD 500 MHz using a 40 second delay using potassium acetate as an internal standard. Quantitative ^{1}H NMR spectra were recorded on a Bruker AVIIIHD 500 MHz using a 30 second delay using potassium acetate as an internal standard.

Solid state NMR spectra were recorded on a Bruker AVIII 400 spectrometer (Operating frequencies; ^{1}H 400.130 MHz, ^{19}F 376.498 MHz, ^{13}C 100.613 MHz, and ^{29}Si 79.495) equipped with a HFXY 3.2 mm probe. Spectra were collected at 298 K and samples were acquired spinning at the magic angle θ = 54.7356 ˚. Values of magic-angle spinning, MAS, speeds, v_{rot}, range between 13 – 20 kHz. Powdered samples were loaded into 3.2 mm ZrO_2 rotors fitted with a Kel-F drive cap. Spectra are reported in chemical shift, δ, in parts per million (ppm) referenced against a secondary external standard ^{1}H adamantane (δ_H = 1.85),[1] ^{19}F PTFE (δ_F = −122.7),[2] ^{29}Si Kaolinite (δ_{Si} = −91.7).[3] Solid state NMR spectra were deconvoluted in OriginPro 2025b version 10.2.5.234 using the peak deconvolution app available in OriginLab. Peaks were modelled as Gaussian Lorentzian Cross Products (parameter definitions: y_0 = baseline, x_c = peak center, A = amplitude, w = width, s = shape):

$$y = y_0 + \frac{A}{1 + e^{0.5(1-s)\left(\frac{x-x_c}{w}\right)^2} s\left(\frac{x - x_c}{w}\right)^2}$$

Ball milling experiments were carried out using either a Retsch MM 400 mixer mill (30 Hz experiments), or a Retsch MM 500 Vario mixer mill (35 Hz experiments). Mechanochemical reactions were carried out in 15 mL FormTech Scientific (FTS) stainless steel jars with hardened chrome grade 100 steel ball bearings (4 g (10 mm), 7 g (12 mm), 16 g (15 mm)), 15 mL FormTech Scientific (FTS) zirconia jar with zirconia balls (12 mm, 15 mm), or 12 mL zirconia jars with zirconia balls (3.2 g). No precaution was taken to exclude air or moisture.

Powder X-ray diffraction (PXRD) data were collected using a Bruker D8 Advance X-ray diffractometer (Bragg−Brentano geometry); the radiations Cu Kα1,2 were used (λ = 1.54056, 1.54439 Å, $K\alpha_1$:$K\alpha_2$ 2:1). The Bruker D8 Advance was equipped with a LYNXEYE XE-T detector with energy threshold filtering of Cu Kβ radiation. The diffractometer was operated with an accelerating voltage of 40 kV and a current of 25 mA. Diffractograms were collected between 5–70° 2θ (6309 steps of 0.0103°), taking a total acquisition time of 583 s (0.0925 s·step^{-1}). Powder samples were mounted onto PMMA specimen sample holders, diffractograms were collected with the specimen rotating at a rate of 2 rpm. Diffractograms were analysed in Malvern Panalytical Xpert Highscore Plus (version: 5.0.0.28712), using the search and match protocol. Raman spectra were recorded using a Thermo Fisher Scientific DXR3 Smart Raman Spectrometer. Spectra were recorded using a 532 nm excitation laser and a 10 μm aperture over a 2 minute acquisition time. Thermogravimetric analysis (TGA) was performed on a TA Discovery TGA 5500 instrument. Powder samples were loaded on a platinum high temperature pan. The samples were heated from 30 to 200 °C at a rate of 20 °C·min^{-1} under N_2 flow (25 mL·min^{-1}). X-Ray photoelectron spectroscopy (XPS) data was collected using a Thermo Scientific K-Alpha XPS equipped with a 180° double focusing hemispherical analyser-128-channel detector and a microfocused monochromated Al X-ray source. Elemental analyses were carried out by Mikroanalytisches Laboratorium Kolbe.

2. Optimisation studies on the degradation of PTFE

The degradation of PTFE was investigated with a range of activators under mechanochemical conditions (ball milling). The amount of activator was calculated based on the monomer unit (CF_2CF_2) of PTFE. The yields of fluoride were calculated with respect to the total fluorine content of PTFE.

2.1 Activator screening

To a 15 mL stainless-steel milling jar was added two hardened chrome steel bearings (2 × 7 g), PTFE (1.00 eq.) and pre-dried activator (1.25 eq. per C–F). The total loading of material in the jar (PTFE and activator) was kept constant at 500 mg, giving a ball-to-powder ratio of 28:1. The jar was closed and securely fitted to the mill which was set for 3 h at a frequency of 35 Hz. Upon completion, the jar was opened and the powder was collected. An aliquot of PTFE-mix (10-30 mg) and sodium triflate (10 mg, as internal standard) was extracted with D_2O (10 atom% D), centrifugated for 15-30 min and analysed by quantitative ^{19}F-NMR spectroscopy. Then, the NMR sample was transferred back to the sample preparation vial, KOH (0.1 mL, 10 M) was added and the mixture was kept still for 6 h and analysed by quantitative ^{19}F-NMR spectroscopy for the [Si-F] release analysis. [Si–F] was calculated by subtracting the qNMR-measured fluoride release before hydrolysis from the fluoride release measured after hydrolysis.

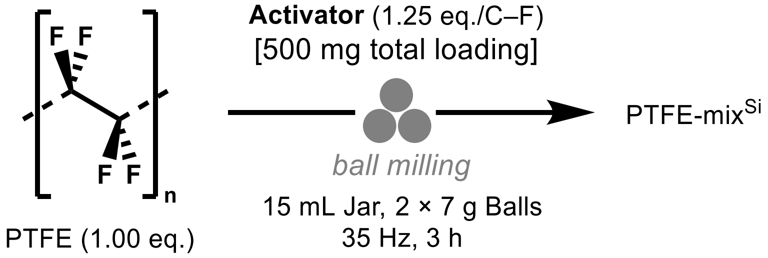

Table S1. Screening of activators for PTFE degradation.[a]

Entry	Activator	F^-
**1**	Na_2SiO_3	100±2%[b]
**2**	K_3PO_4	84% (+15% PO_3F^{2-})
**3**	Na_3PO_4	66%
**4**	SiO_2	71%[c]
**5**	none	n.d.
**6**[d]	Na_2SiO_3	quant.
**7**	Na_2CO_3	27%
**8**	K_2SiO_3	84% (+16% Si–F)
**9**	NaOH	4%
**10**	KOH	10%

[a] Yields were determined by quantitative ^{19}F-NMR spectroscopy in D_2O (10 atom% D) using sodium triflate as internal standard; n.d. = not detected. [b] reported as a mean value of triplicate values with one standard deviation. [c] the yield of Si–F was determined by addition of KOH (0.1 ml, 10 M) into an aliquot of PTFE-mix sample. [d] 15 ml zirconia jar and 2 × 6 g zirconia balls were used instead of 15 ml stainless-steel jar and 2 × 7 g chrome steel balls.

2.2 Optimisation of activator stoichiometry

To a 15 mL stainless-steel milling jar was added two hardened chrome steel bearings (2 × 7 g), PTFE (1.00 eq.) and Na_2SiO_3 (x eq. per C–F). The total loading of material in the jar (PTFE and Na_2SiO_3) was kept constant at 500 mg. The jar was closed and securely fitted to the mill which was set for 3 h at a frequency of 35 Hz. Upon completion, the jar was opened and the powder was collected. An aliquot of PTFE-mix^{Si} (10-30 mg) and sodium triflate (10 mg, as internal standard) was extracted with D_2O (10 atom% D), centrifugated for 15-30 min and analysed by quantitative ^{19}F-NMR spectroscopy.

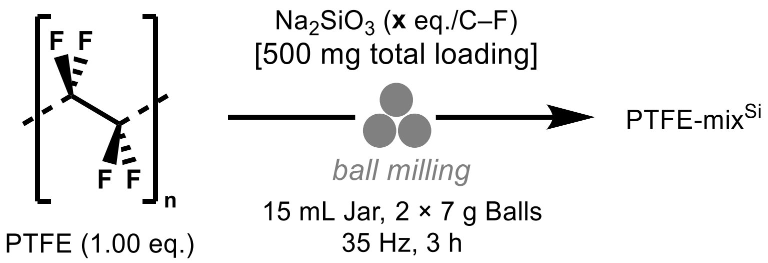

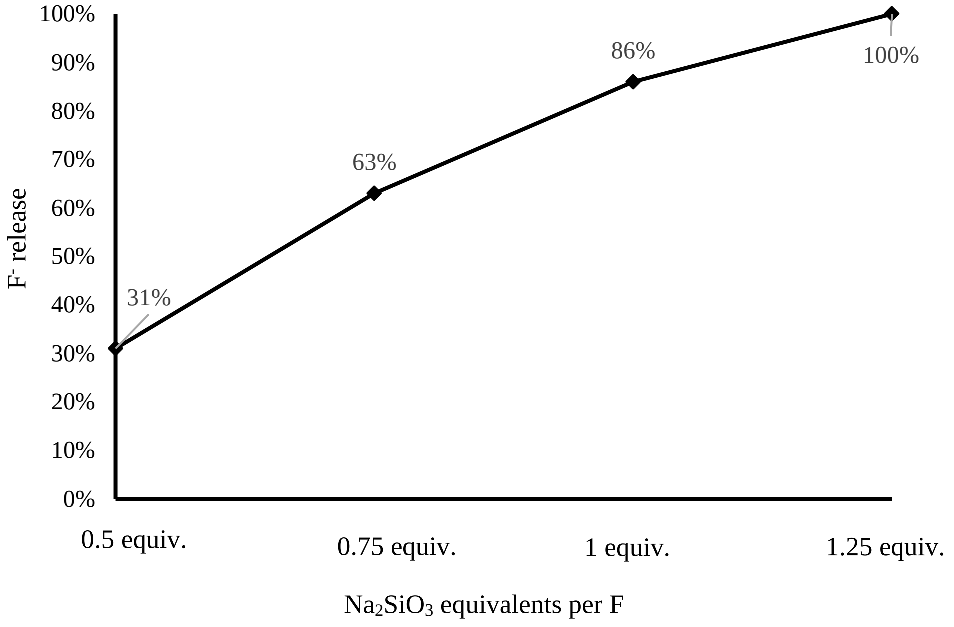

Figure S1. Optimisation of Na_2SiO_3 equivalents for PTFE-mix^{Si} formation. [a] Fluoride release quantified as water soluble F^- determined by quantitative ^{19}F NMR spectroscopy, expressed as percent of the total fluorine content of PTFE.

2.3 Mechanochemical PTFE Activation Time-Course

To a 15 mL stainless-steel milling jar was added two hardened chrome steel bearings (2 × 7 g), PTFE (1.00 eq.) and Na_2SiO_3 (1.25 eq. per C–F). The total loading of material in the jar (PTFE and Na_2SiO_3) was kept constant at 500 mg. The jar was closed and securely fitted to the mill which was set for *y* h at a frequency of 35 Hz. Upon completion, the jar was opened and the powder was collected. An aliquot of PTFE-mix^{Si} (10-30 mg) and sodium triflate (10 mg, as internal standard) was extracted with D_2O (10 atom% D), centrifugated for 15-30 min and analysed by quantitative ^{19}F-NMR spectroscopy.

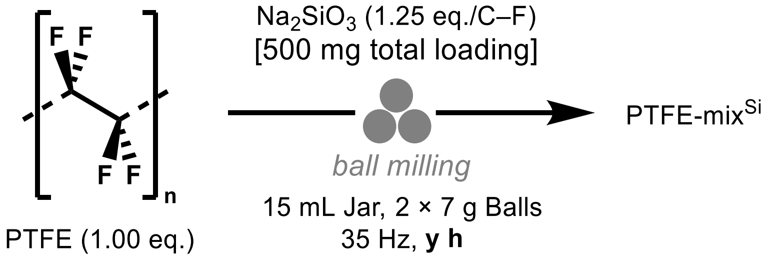

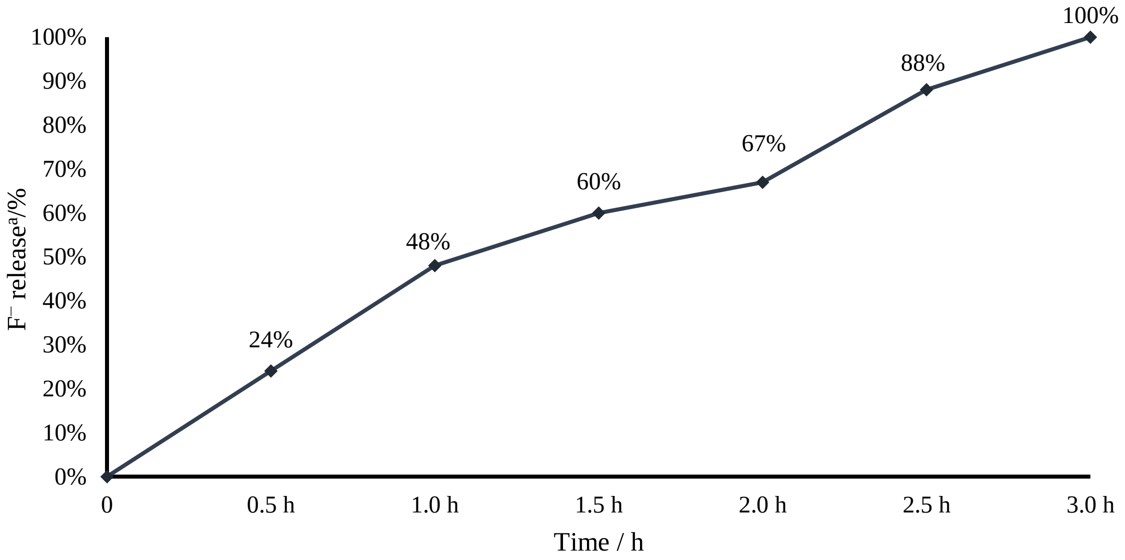

Figure S2. Mechanochemical activation of PTFE time-course using Na_2SiO_3 as activator. [a] Fluoride release quantified as water soluble F^- determined by quantitative ^{19}F NMR spectroscopy, expressed as percent of the total fluorine content of PTFE.

2.4 Effect of Jar Loading on Fluoride Release

To a 15 mL stainless-steel milling jar was added two hardened chrome steel bearings (2 × 7 g), PTFE (1.00 eq.) and Na_2SiO_3 (1.25 eq. per C–F). The total loading of material in the jar (PTFE and Na_2SiO_3) was varied from 500 mg to 2 g. The jar was closed and securely fitted to the mill which was set for 3 h at a frequency of 35 Hz. Upon completion, the jar was opened and the powder was collected. An aliquot of PTFE-mix^{Si} (10-30 mg) and sodium triflate (10 mg, as internal standard) was extracted with D_2O (10 atom% D), centrifugated for 15-30 min and analysed by quantitative ^{19}F-NMR spectroscopy.

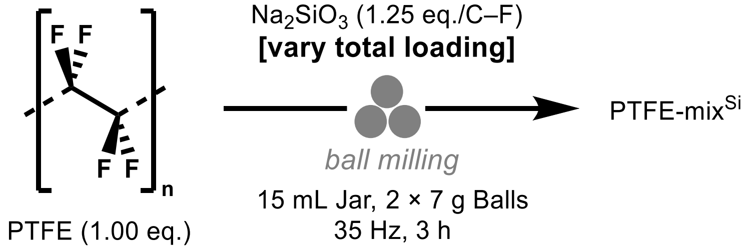

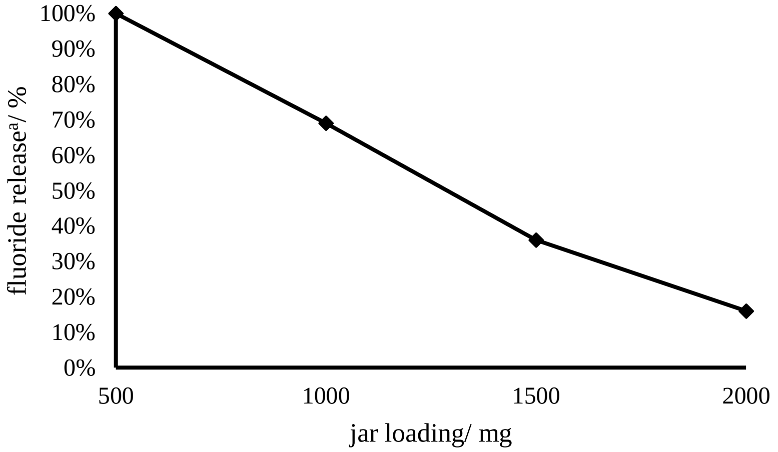

Figure S3. Effect of jar loading on fluoride release from PTFE using Na_2SiO_3 as activator. [a] Fluoride release quantified as water soluble F^- determined by quantitative ^{19}F NMR spectroscopy, expressed as percent of the total fluorine content of PTFE.

3. Composition of PTFE-mix^{Si}

3.1 Solution-Phase qNMR Spectroscopy

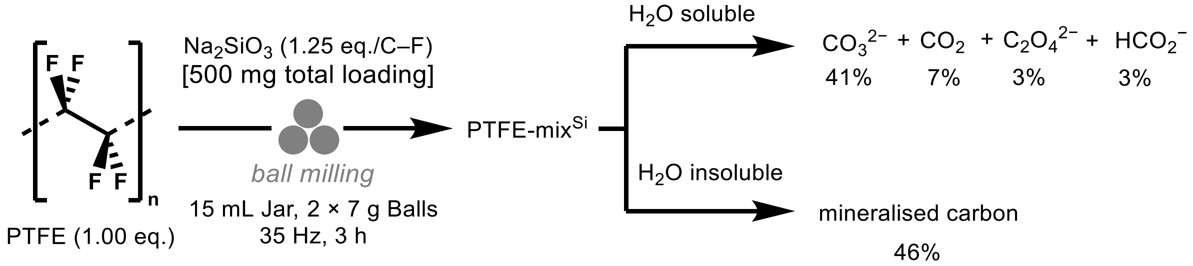

To a 15 mL stainless-steel milling jar was added two hardened chrome steel bearings (2 × 7 g), PTFE (1.00 eq., 70 mg) and Na_2SiO_3 (1.25 eq./F, 430 mg). The jar was closed and securely fitted to the mill which was set for 3 h at a frequency of 35 Hz. Upon completion, the jar was opened and the powder was collected and extracted with H_2O/D_2O = 9/1 (5 mL). Subsequently, KOAc (200 µL, 0.509 M solution in D_2O) was added as internal standard, the sample was concentrated to a volume of approx. 1.5 mL and centrifugated. The clear supernatant was analysed by NMR spectroscopy. Following the removal of the water-soluble fraction, a water-insoluble material (23.7 mg) was obtained and analysed by Raman spectroscopy (Section 3.2, Figure S5) and X-ray photoelectron spectroscopy.

For quantification of CO_2 formation, CO_2 was captured using NaOH (100 mg mL^{-1}, 1 mL), and quantified as Na_2CO_3. CO_2 accounts for 7% of the total carbon content.

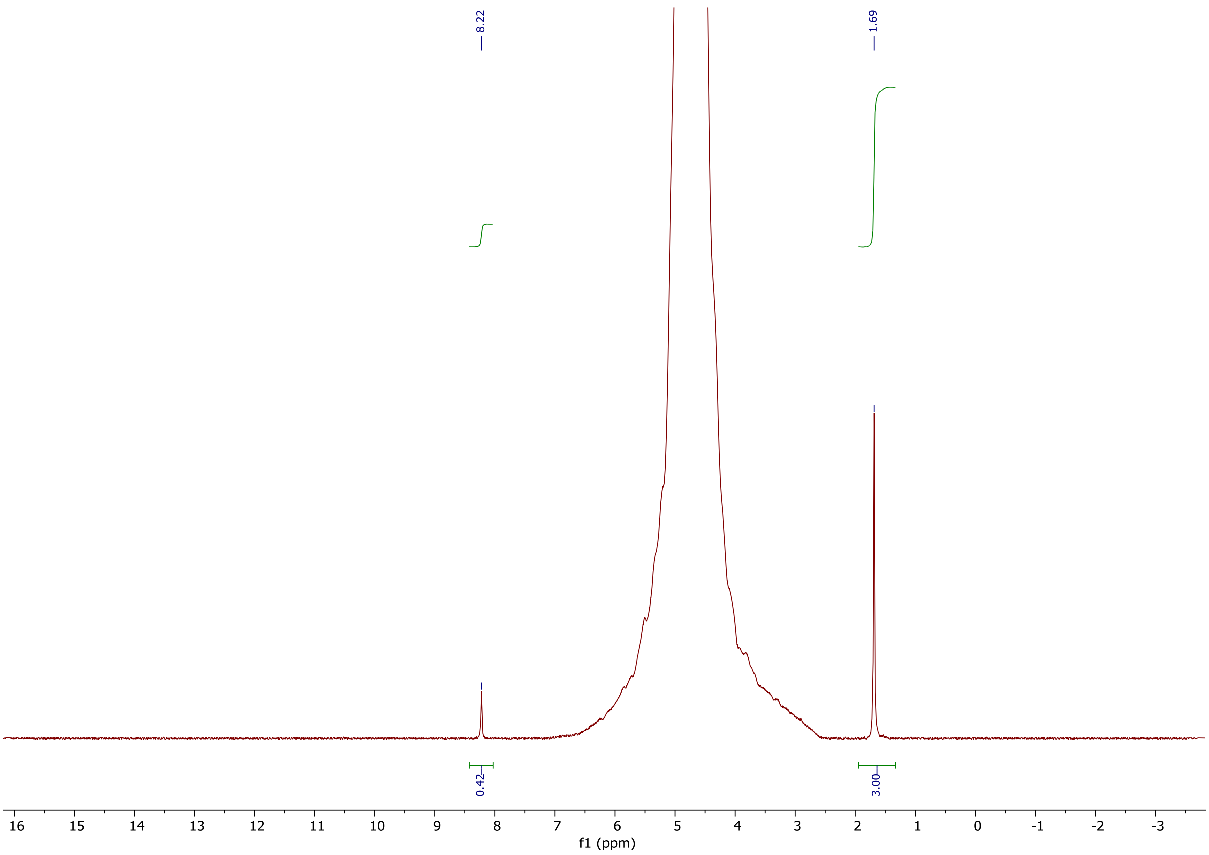

Figure S4. Quantitative ^1H NMR spectrum of an aliquot of PTFE-mix^{Si} in D_2O (10 atom% D) with KOAc as internal standard (δ_H = 1.70 ppm) indicates traces of formate (δ_H = 8.22 ppm, 3%).

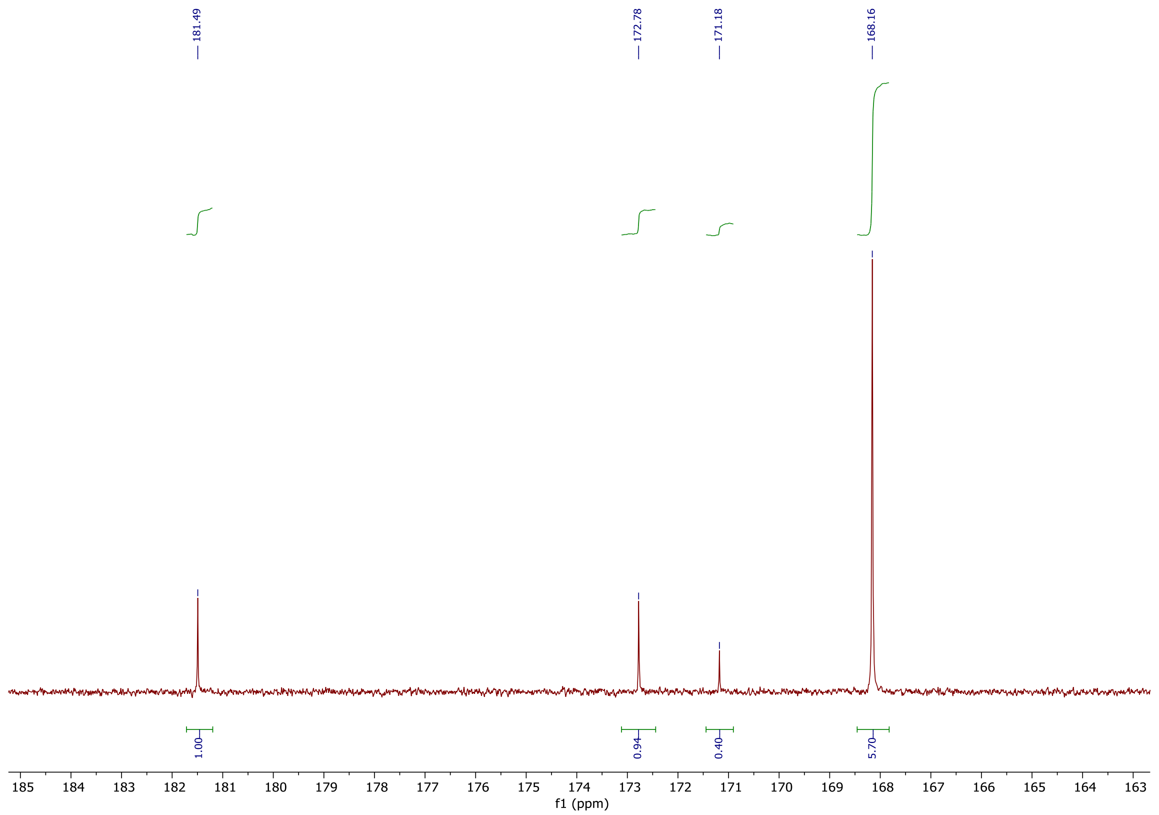

Figure S5. Quantitative $^{13}C\{^1H\}$ NMR spectrum of a whole jar of PTFE-mix^{Si} in D_2O (10 atom% D) with KOAc as internal standard (δ_C = 181.5 ppm), indicating the presence of oxalate (δ_C = 172.8 ppm, 3%), formate (δ_C = 171.2, 3%), and carbonate (δ_C = 168.2 ppm, 41%).

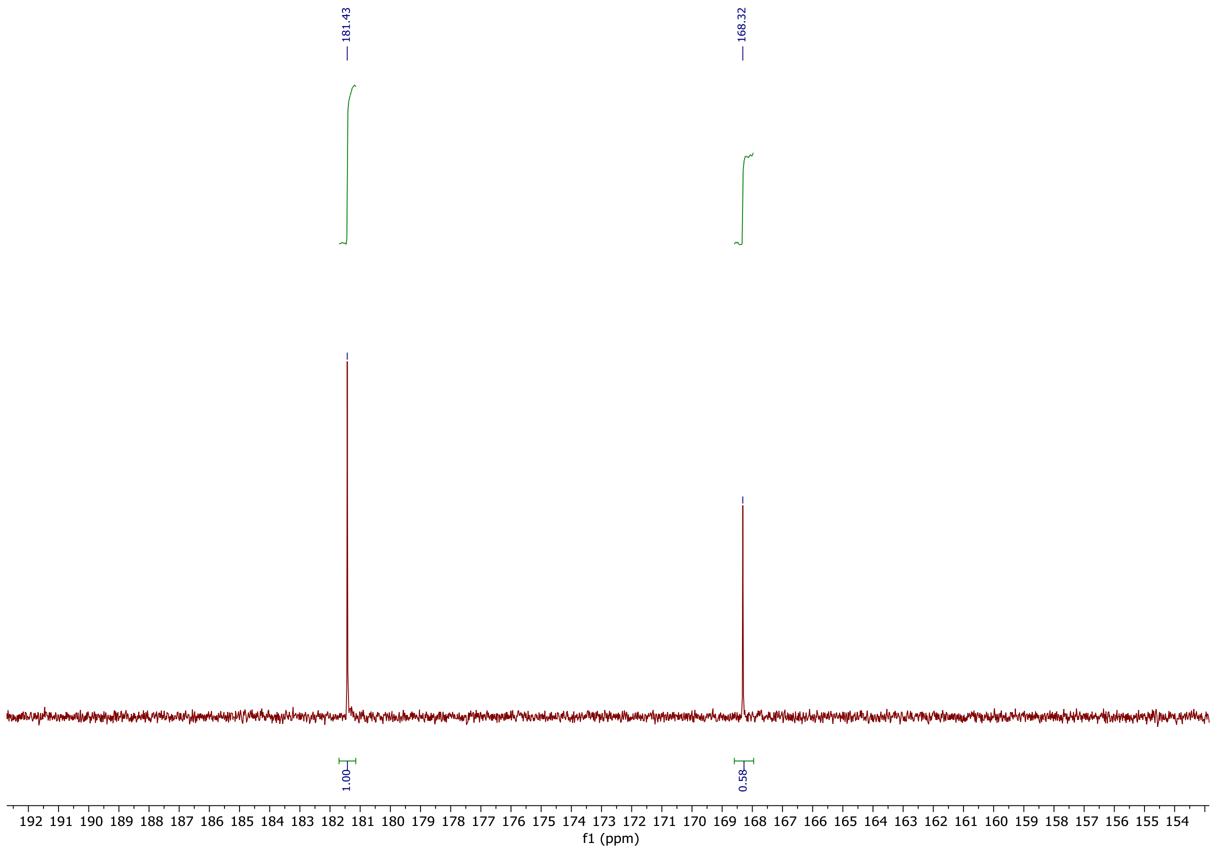

Figure S6: Quantitative $^{13}C\{^1H\}$ NMR spectrum of a 600 μL aliquot of the 1 mL NaOH solution (100 mg mL^{-1}) with 200 μL KOAc as internal standard in H_2O/D_2O = 9/1, showing CO_2 captured as carbonate (δ_C = 168.3 ppm, 7%)

3.2 Solid-State Analysis of Carbon Black

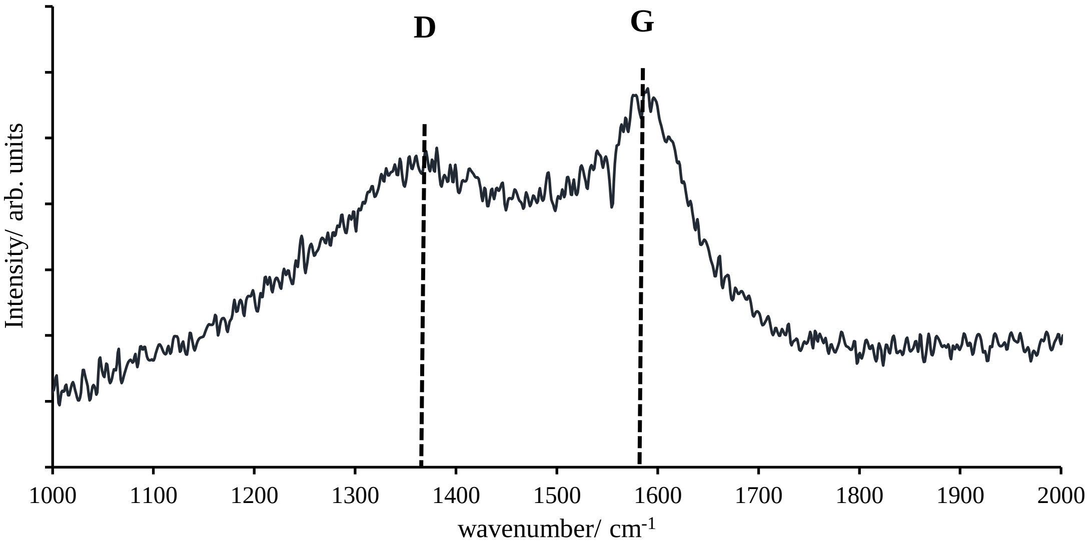

Figure S7. Raman spectrum of isolated water-insoluble black residue from the whole jar PTFE-mix^{Si}, indicating the presence of carbon, isolated yield 53%. Disordered (D) and graphitic (G) vibrational modes have been labelled.

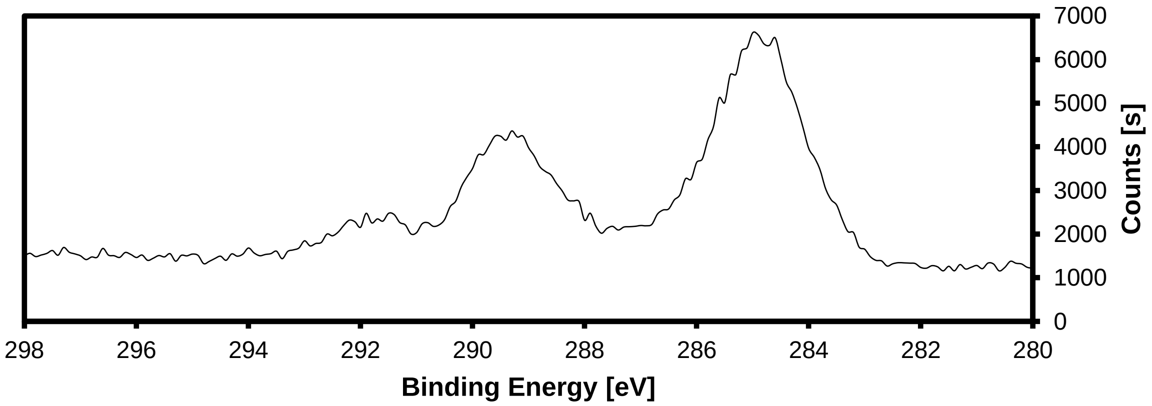

Figure S8: C(1s) XPS survey of the insoluble material, showing the presence of (C)-C (285 eV), (C)-O (289.5 eV), (C)-F (292 eV) species

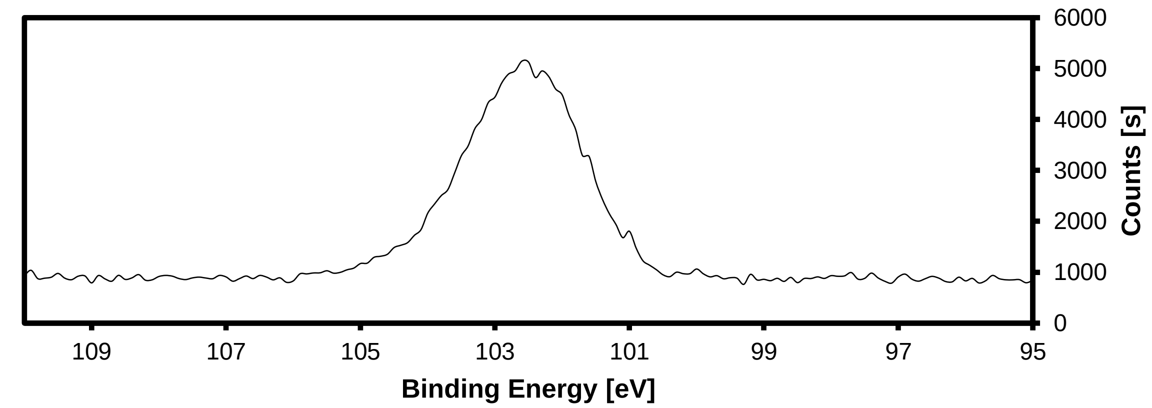

Figure S9: Si(2p) XPS survey of the insoluble material, showing the presence of (Si)-O (103 eV) species

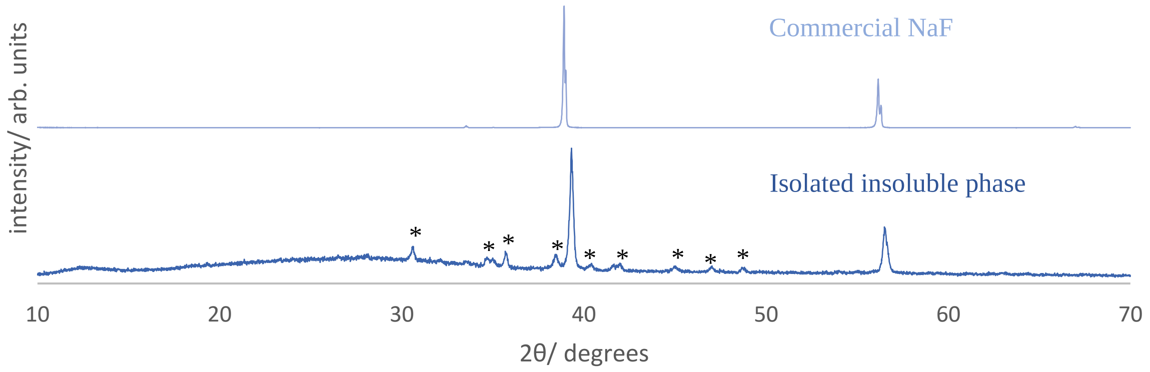

Figure S10: XRD of commercial NaF (top), water-insoluble phase (bottom), showing residual NaF. Minor peaks associated with Na_2CO_3 are marked with an asterisk (*)

Table S2: Elemental analyses of isolated insoluble phase (performed in duplicates)

Element	C (theoretical)	Isolated insoluble phase
C (wt%)	100	50.41 (50.47)
F (wt%)	n/a	21.01 (21.02)
Na (wt%)	n/a	25.45 (25.46)
Si (wt%)	n/a	1.57 (1.59)
Fe (wt%)	n/a	0.38 (0.39)

3.3 Powder X-ray Diffraction of PTFE-mix^{Si}

To a 15 mL stainless-steel milling jar was added two hardened chrome steel bearings (2 × 7 g), PTFE (1.00 eq.) and Na_2SiO_3 (1.25 eq./F). The total loading of material in the jar (PTFE and activator) was kept constant at 500 mg. The jar was closed and securely fitted to the mill which was set for 3 h at a frequency of 35 Hz. Upon completion, the jar was opened and the powder was collected and analysed by PXRD.

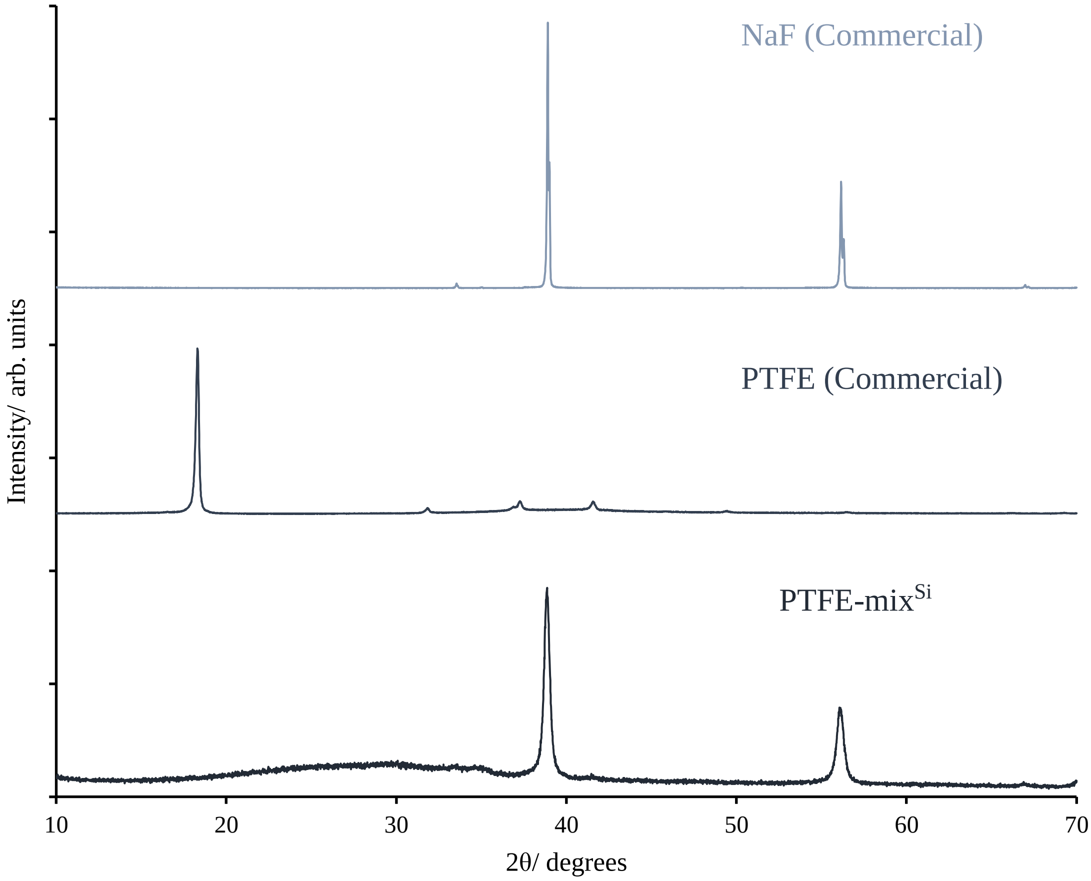

Figure S11. Powder X-ray diffractorgam of NaF (Commercial, Top), PTFE (Commercial, Middle), and PTFE-mix^{Si} (Bottom).

3.4 FTIR Spectra of PTFE-mix^{Si}

To a 15 mL stainless-steel milling jar was added two hardened chrome steel bearings (2 × 7 g), PTFE (1.00 eq.) and Na_2SiO_3 (1.25 eq./F). The total loading of material in the jar (PTFE and activator) was kept constant at 500 mg. The jar was closed and securely fitted to the mill which was set for 3 h at a frequency of 35 Hz. Upon completion, the jar was opened and the powder was collected and analysed by FTIR spectra.

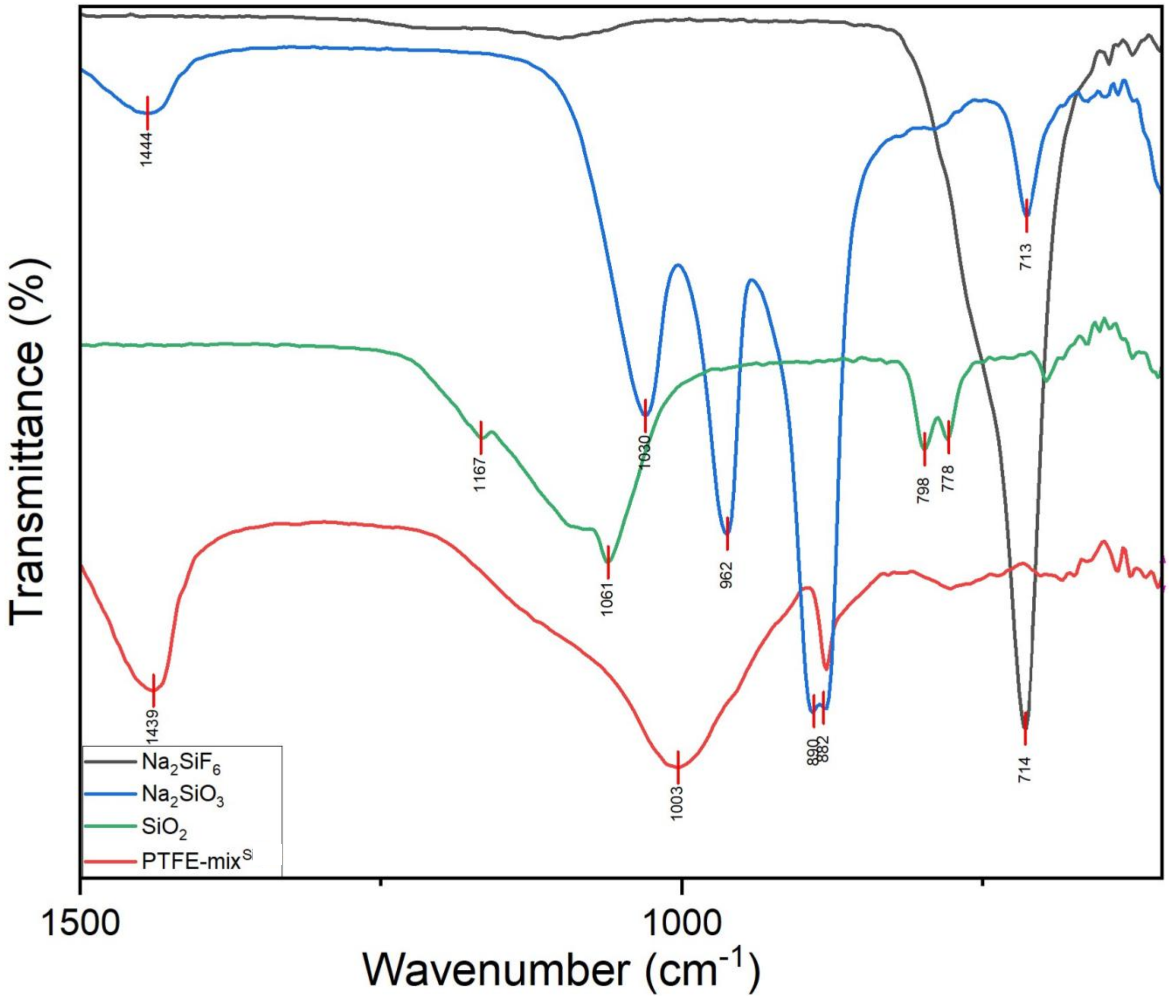

Figure S12. FTIR spectra of Na_2SiF_6, Na_2SiO_3, SiO_2 and PTFE-mix^{Si} (top to bottom) indicates the absence of Na_2SiF_6 formation and show a broad SiO_2-related peak (1000−1250 cm^{-1}) in the PTFE-mix^{Si} sample.

3.5 Solid-state NMR spectroscopy of PTFE-mix^{Si}

To a 15 mL stainless-steel milling jar was added two chrome steel bearings (2 × 7 g), PTFE (1.00 eq.) and silicate (1.25 eq./F). The total loading of material in the jar (PTFE and activator) was kept constant at 500 mg. The jar was closed and securely fitted to the mill which was set for 3 h at a frequency of 35 Hz. Upon completion, the jar was opened and the powder was collected and analysed by solid-state NMR spectroscopy.

SS NMR spectra were collected on powders obtained after mechanical activation of PTFE with 5 equivalents of activator: Na_2SiO_3. PTFE activated with sodium silicate showed the formation of the respective alkali metal fluoride, with no evidence of any Si–F bond formation.

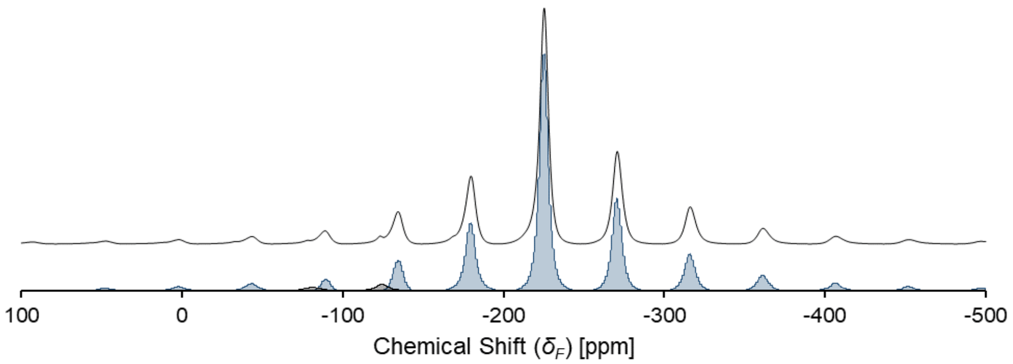

Figure S13. ^{19}F DPMAS SS NMR spectra of the powder obtained after mechanical activation of PTFE with 5 equivalents of Na_2SiO_3. Deconvolution of the spectra revealed 2 isotropic resonances NaF (Blue, $\delta_F = -225.4$, 97%), and traces of PTFE (Black, $\delta_F = -123.4$, 3%).[4]

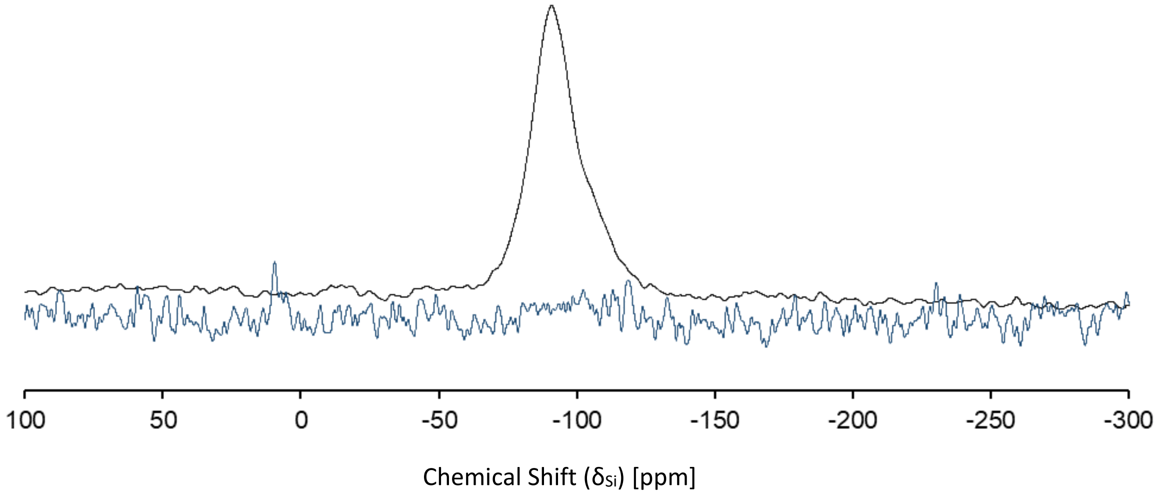

Figure S14. ^{29}Si{^{19}F} SS NMR spectra with high power decoupling (Black) or cross-polarisation from ^{19}F (Blue). The CP spectra shows no evidence of Si–F containing species, all signals can be assigned as Si–O silicate resonances.

4. Isolation of Alkali Metal Fluorides

4.1 Isolation of NaF from PFAS-mix^{Si}

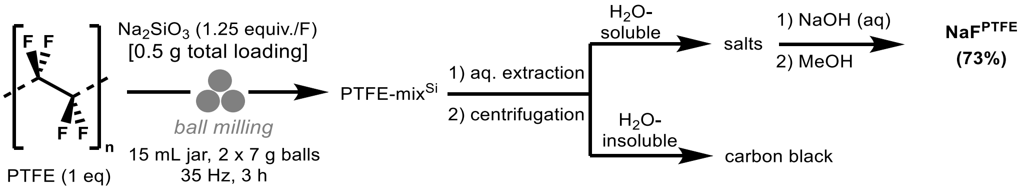

To three 15 mL stainless-steel milling jars was added two chrome steel balls (2 × 7 g), PTFE (1 eq., 70 mg) and Na_2SiO_3 (1.25 eq./F, 430 mg) each. The jars were closed and securely fitted to a mill which was set for 3 h at a frequency of 35 Hz. Upon completion, the jars were opened, the powder was collected and extracted with H_2O (20 mL). The resulting suspension was centrifuged for 30 min at room temperature to eliminate water-insoluble carbon black. The resulting clear supernatant was decanted and concentrated under reduced pressure to afford a solid residue. This residue was treated with aqueous NaOH (3.5 mL, 0.5 g/mL) and heated for 5 min using a heat gun to facilitate dissolution. The solution was then allowed to cool to room temperature and transferred to 3 centrifuge vials (1.5 mL) for centrifugation 10 min, resulting in the precipitation of solid. The isolated solid was resuspended in aqueous NaOH (1.5 mL, 0.5 g/mL) to form a slurry and centrifuged again for 10 min, yielding

crude sodium fluoride (NaF) as a precipitate. The supernatant was decanted, and the remaining solid was washed thoroughly with MeOH (5 × 1.5 mL) to remove residual NaOH. The resulting solid was dried under vacuum to yield pure NaF (277 mg) in 73% yield (93% purity determined by quantitative ^{19}F NMR spectroscopy). The contaminants of the isolated NaF are likely Na_2SiO_3 and silicon oxides.

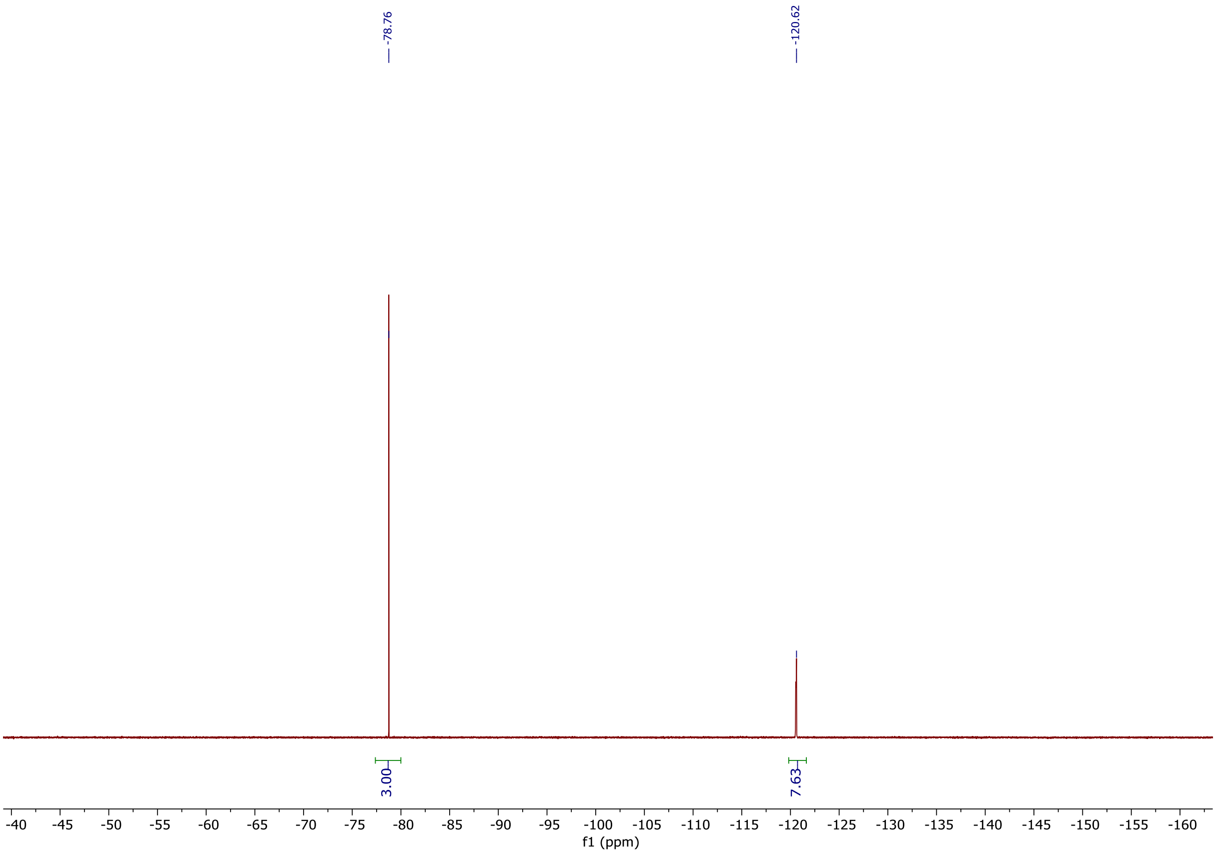

Figure S15. Quantitative ^{19}F NMR spectrum (377 MHz) of 14 mg NaF^{PTFE} and 10 mg NaOTf (as an internal standard) in D_2O (10 atom% D).

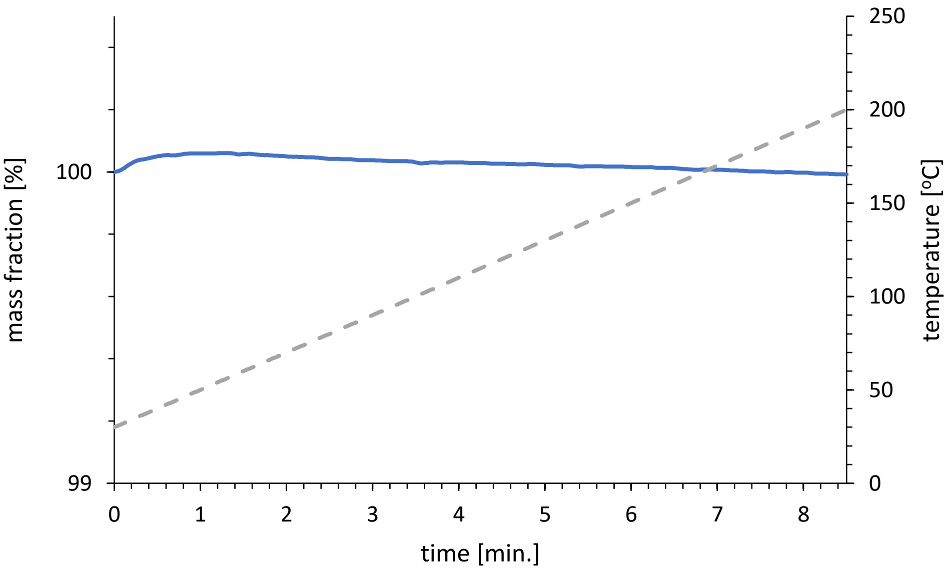

Figure S16: TGA analysis of approx.. 50 mg NaF^{PTFE}, indicating <0.1% mass lost. The solid line refers to the mass fraction of the sample; the dashed line indicates temperature gradient.

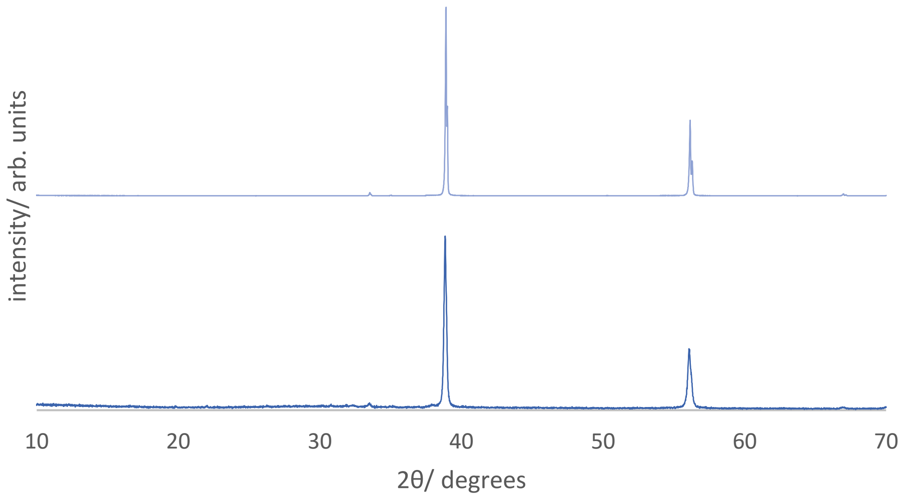

Figure S17: PXRD traces of commercial NaF (top), and NaF isolated from PTFE (bottom)

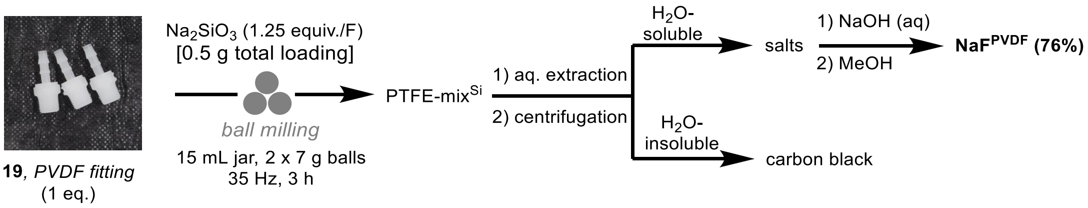

To three 15 mL stainless-steel milling jars was added two chrome steel balls (2 × 7 g), PVDF fitting (1 eq., 87 mg) and Na_2SiO_3 (1.25 eq./F, 413 mg) each. The jars were closed and securely fitted to a mill which was set for 3 h at a frequency of 35 Hz. Upon completion, the jars were opened, the powder was collected and extracted with H_2O (20 mL). The resulting suspension was centrifuged for 30 min at room temperature to eliminate water-insoluble carbon black. The resulting clear supernatant was decanted and concentrated under reduced pressure to afford a solid residue. This residue was treated with aqueous NaOH (3.5 mL, 0.5 g/mL) and heated for 5 min using a heat gun to facilitate dissolution. The solution was then allowed to cool to room temperature and transferred to 3 centrifuge vials (1.5 mL) for centrifugation 10 min, resulting in the precipitation of solid. The isolated solid was resuspended in aqueous NaOH (1.5 mL, 0.5 g/mL) to form a slurry and centrifuged again for 10 min, yielding crude sodium fluoride (NaF) as a precipitate. The supernatant was decanted, and the remaining solid was washed thoroughly with MeOH (5 × 1.5 mL) to remove residual NaOH. The resulting solid was dried under vacuum to yield pure NaF (270 mg) in 76% yield (96% purity determined by quantitative ^{19}F NMR spectroscopy).

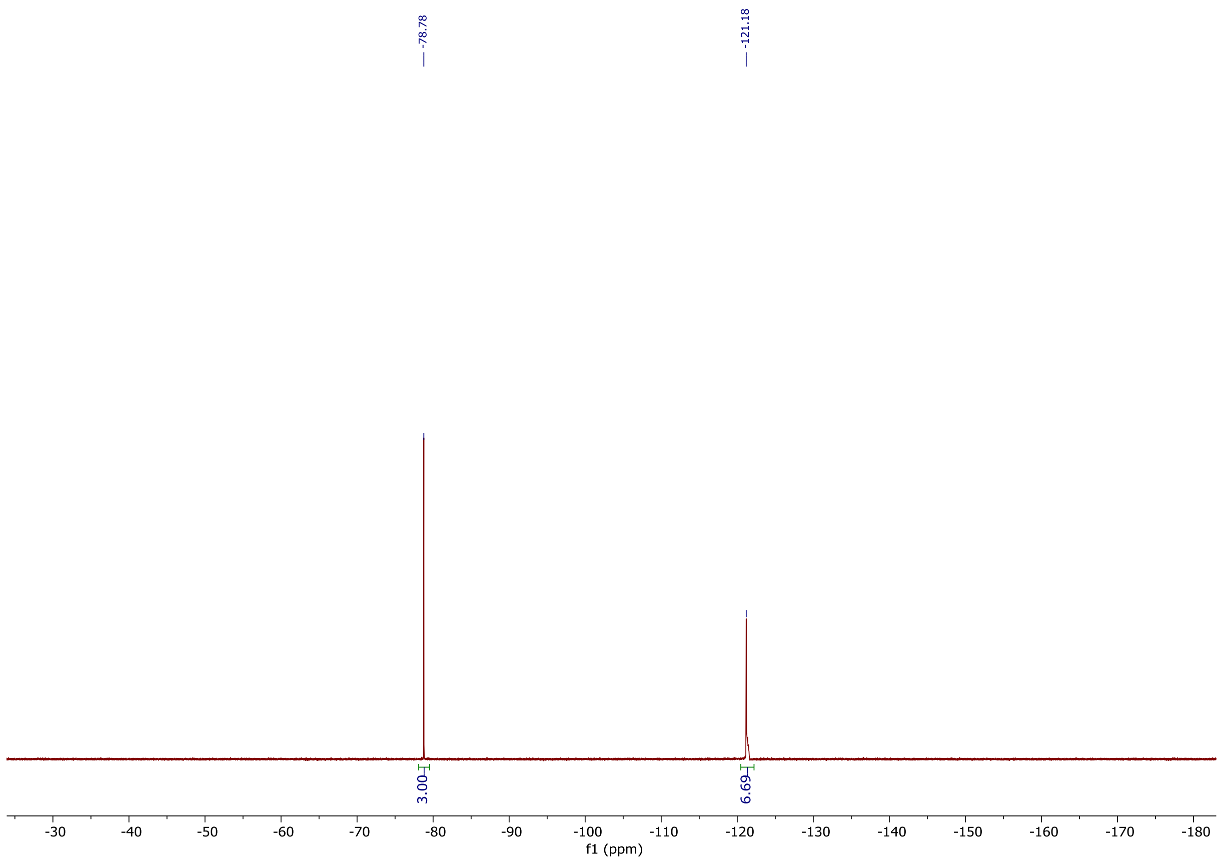

Figure S18. Quantitative ^{19}F NMR spectrum (377 MHz) of 17 mg NaF^{PVDF} and 10 mg NaOTf (as an internal standard) in D_2O (10 atom% D).

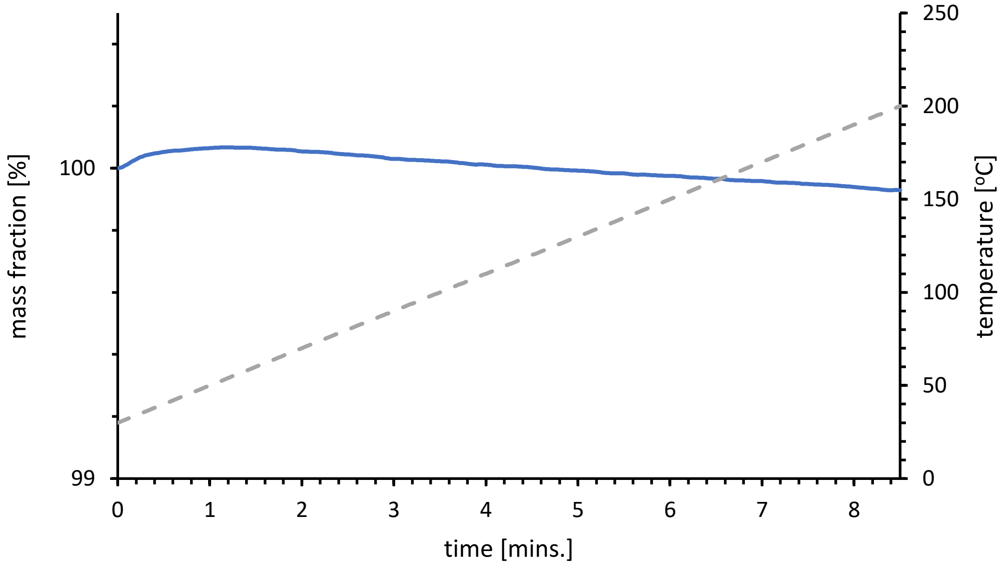

Figure S19: TGA analysis of approx. 50 mg NaF^{PVDF}, indicating <0.1% mass lost. The solid line refers to the mass fraction of the sample; the dashed line indicates temperature gradient.

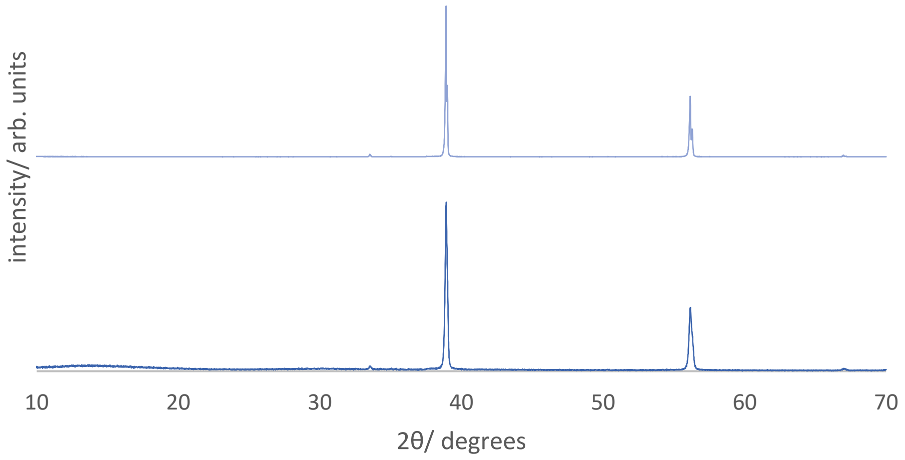

Figure S20: PXRD traces of commercial NaF (top), and NaF isolated from PVDF (bottom)

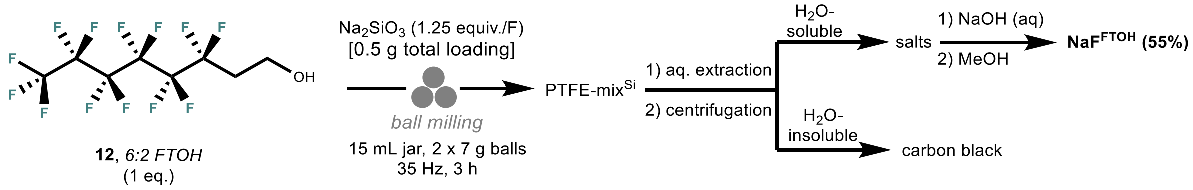

To three 15 mL stainless-steel milling jars was added two chrome steel balls (2 × 7 g), 6:2 FTOH (1 eq., 77.5 mg) and Na_2SiO_3 (1.25 eq./F, 422.5 mg) each. The jars were closed and securely fitted to a mill which was set for 3 h at a frequency of 35 Hz. Upon completion, the jars were opened, the powder was collected and extracted with H_2O (20 mL). The resulting suspension was centrifuged for 30 min at room temperature to eliminate water-insoluble carbon black. The resulting clear supernatant was decanted and concentrated under reduced pressure to afford a solid residue. This residue was treated with aqueous NaOH (3.5 mL, 0.5 g/mL) and heated for 5 min using a heat gun to facilitate dissolution. The solution was then allowed to cool to room temperature and transferred to 3 centrifuge vials (1.5 mL) for centrifugation 10 min, resulting in the precipitation of solid. The isolated solid was resuspended in aqueous NaOH (2.5 mL, 0.5 g/mL) to form a slurry and centrifuged again for 10 min, yielding crude sodium fluoride (NaF) as a precipitate. The supernatant was decanted, and the remaining solid was washed thoroughly with MeOH (5 × 1.5 mL) to remove residual NaOH. The resulting solid was dried under vacuum to yield pure NaF (210 mg) in 55% yield (92% purity determined by quantitative ^{19}F NMR spectroscopy).

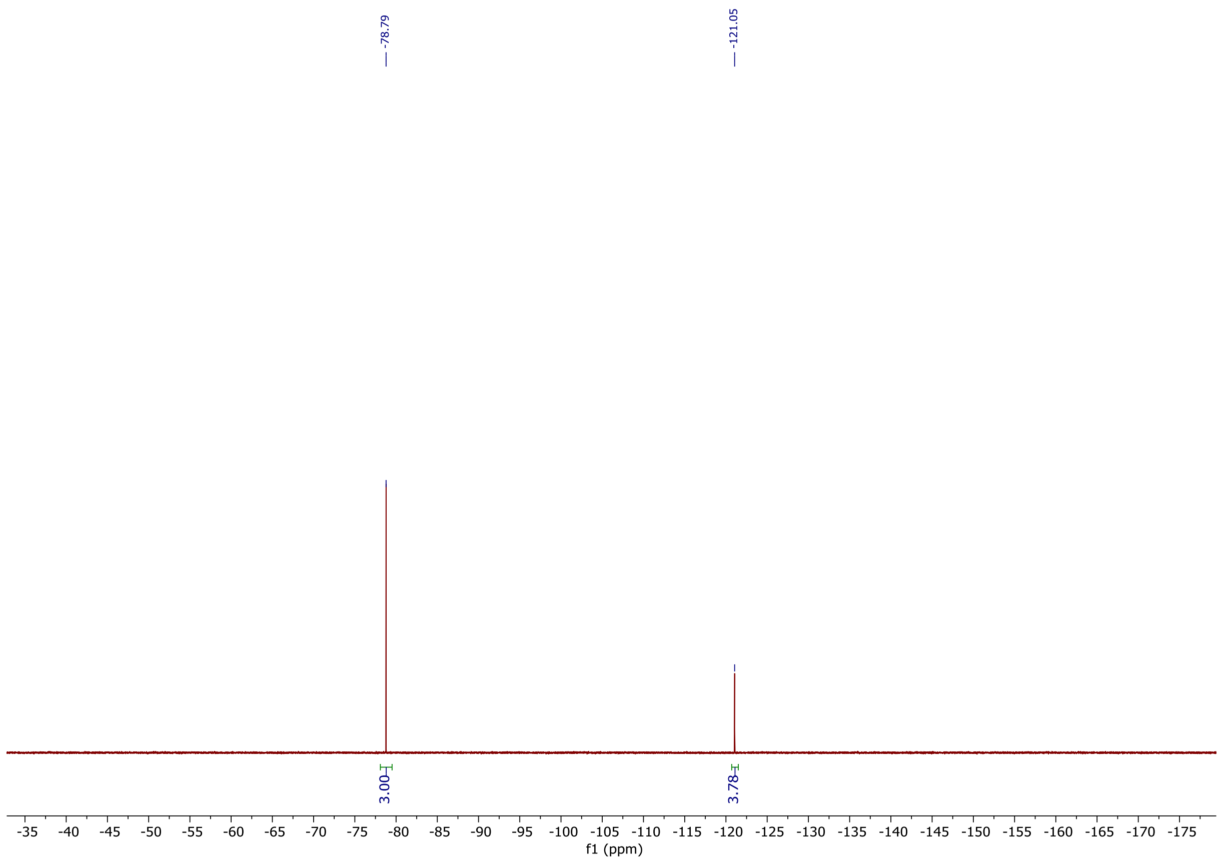

Figure S21. Quantitative ^{19}F NMR spectrum (377 MHz) of 10 mg NaF^{FTOH} and 10 mg NaOTf (as an internal standard) in D_2O (10 atom% D).

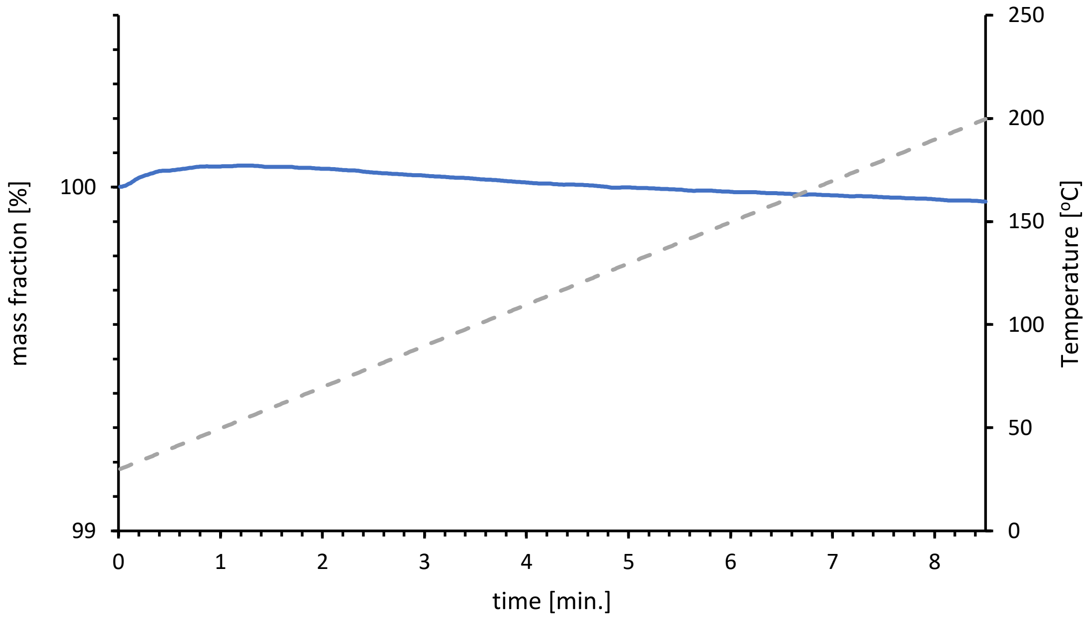

Figure S22: TGA analysis of approx. 50 mg NaF^{FTOH}, indicating <0.1% mass lost. The solid line refers to the mass fraction of the sample; the dashed line indicates temperature gradient.

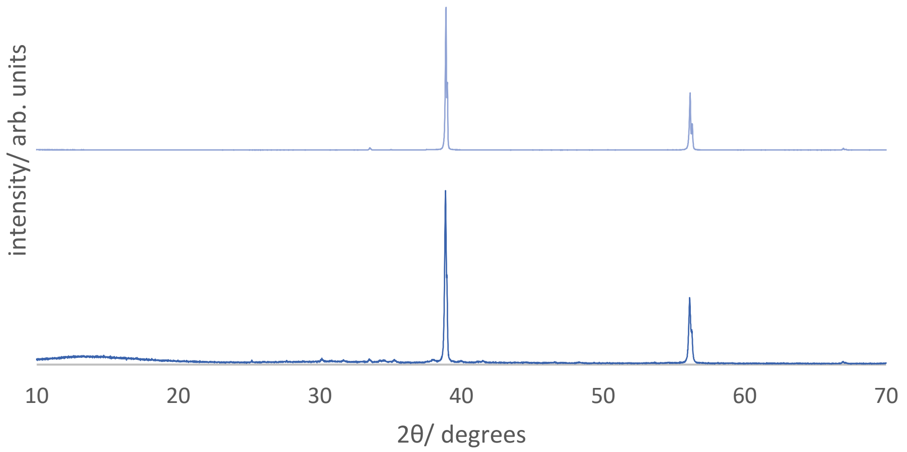

Figure S23: PXRD traces of commercial NaF (top), and NaF isolated from 6:2-FTOH (bottom)

4.2 Scaled-Up Isolation of NaF from PFAS-mix^{Si}

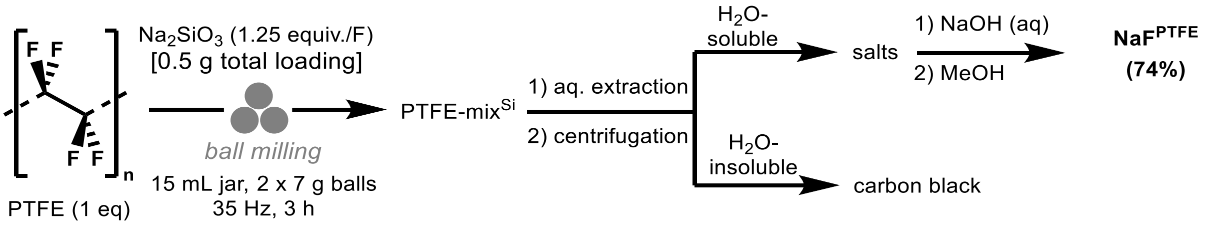

To eleven 15 mL stainless-steel milling jars was added two chrome steel balls (2 × 7 g), PTFE (1 eq., 70 mg) and Na_2SiO_3 (1.25 eq./F, 430 mg) each. The jars were closed and securely fitted to a mill which was set for 3 h at a frequency of 35 Hz. Upon completion, the jars were opened, the powder was collected and extracted with H_2O (80 mL). The resulting suspension was centrifuged for 30 min at room temperature to eliminate water-insoluble species. The resulting clear supernatant was decanted and concentrated under reduced pressure to afford a solid residue. This residue was treated with aqueous NaOH (14 mL, 0.5 g/mL) and heated for 5 min using a heat gun to facilitate dissolution. The solution was then allowed to cool to room temperature and transferred to a falcon tube (15 mL) and centrifuged for 10 mins. The isolated solid was resuspended in aqueous NaOH (6 mL, 0.5 g/mL) to form a slurry and centrifuged again for 10 min, yielding crude sodium fluoride (NaF) as a precipitate. The supernatant was decanted, and the remaining solid was washed thoroughly with MeOH (5 × 6 mL) to remove residual NaOH. The resulting solid was dried under vacuum to yield pure NaF (957 mg) in 74% yield (97% purity determined by quantitative ^{19}F NMR spectroscopy).

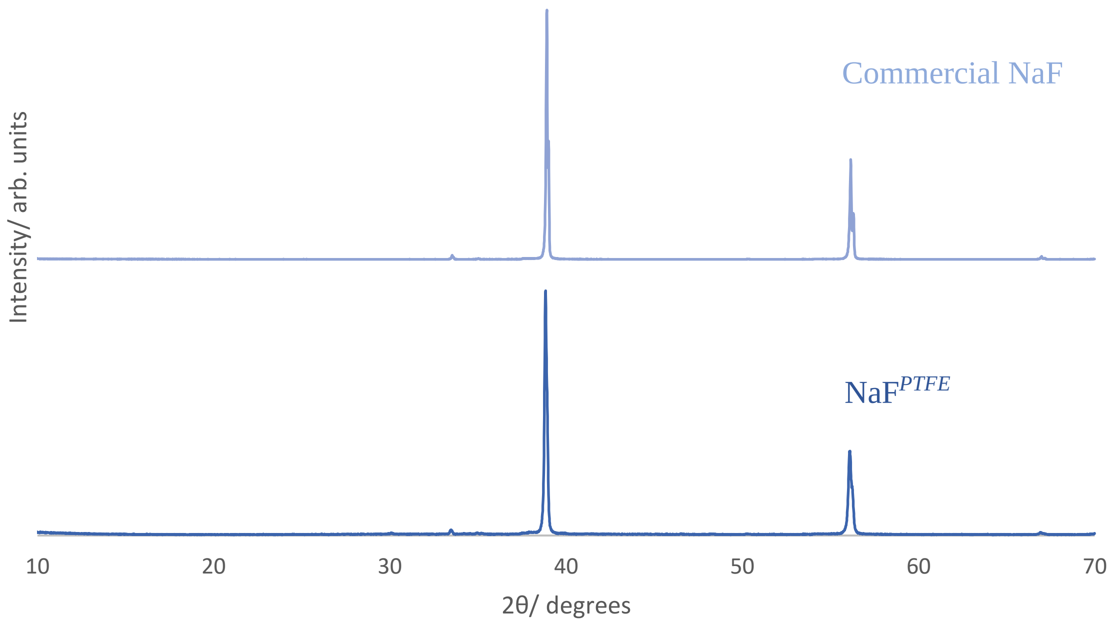

Figure S24: PXRD traces of commercial NaF (top), and NaF isolated from PTFE (bottom)

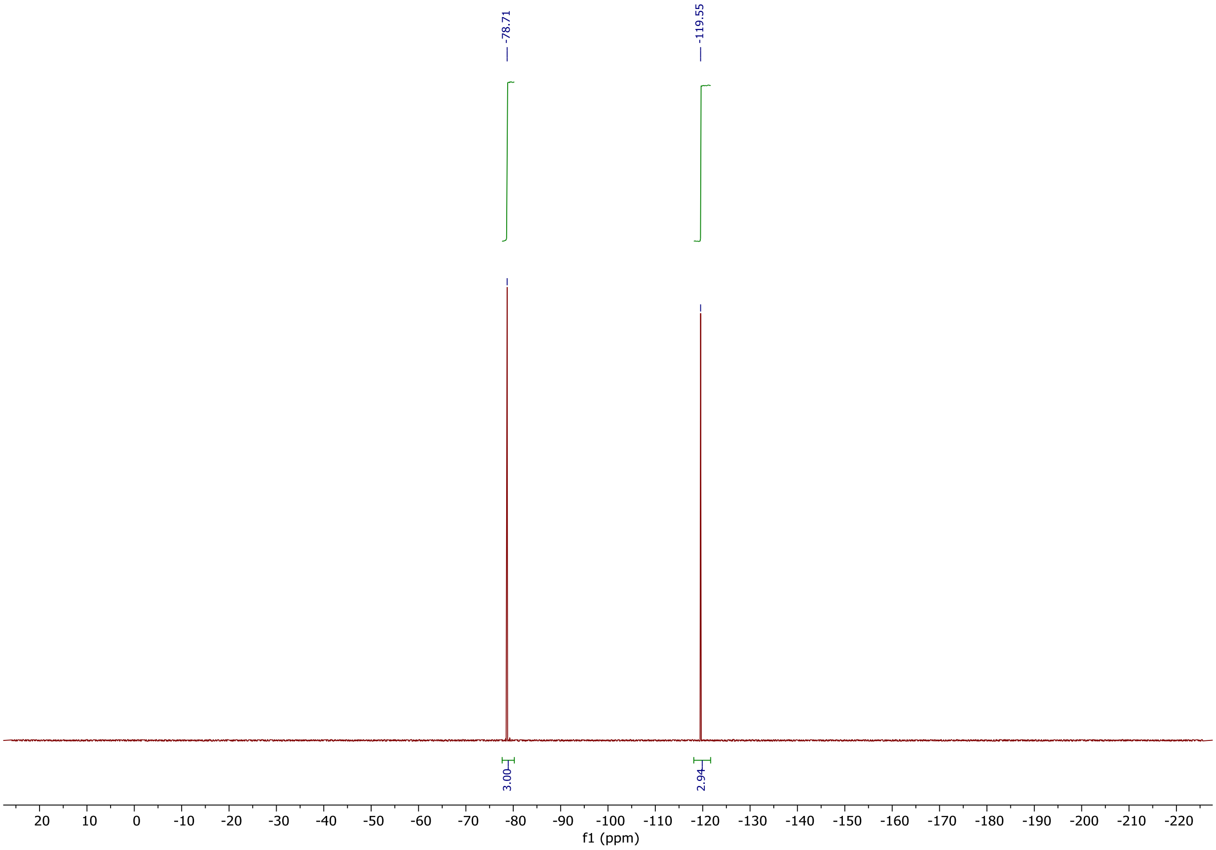

Figure S25: Quantitative ^{19}F NMR spectrum (377 MHz) of 16.6 mg NaF^{PTFE} and 22.4 mg NaOTf (as an internal standard) in D_2O (10 atom% D).

4.3. Elemental analysis of NaF^{PTFE}

Elemental analyses (F, Na) of commercial NaF sample, and NaF^{PTFE} was carried out by Mikroanalytisches Laboratorium Kolbe.

Table S3. Elemental analyses of NaF^{PTFE} sample performed in duplicates.

Element	NaF (theoretical)	NaF Merck 99% (metals basis)	NaF^{PTFE}
F (wt%)	45.25	45.20	43.99 (44.02)
Na (wt%)	54.75	54.67	53.18 (53.17)
C (wt%)	n/a	n/a	0.89 (0.87)
Si (wt%)	n/a	n/a	0.21 (0.20)
Fe (wt%)	n/a	n/a	0.09 (0.10)

4.4 Isolation of KF from $PFAS\text{-}mix^{Si}$

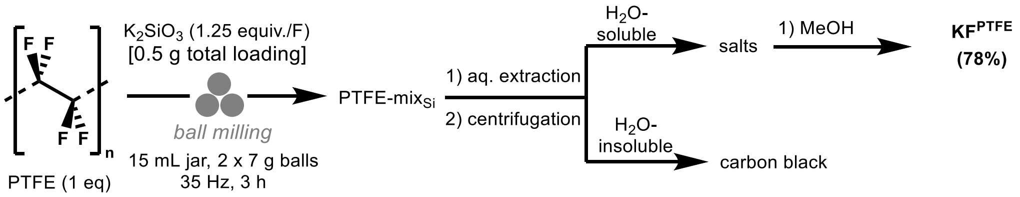

To a 15 mL stainless-steel milling jar was added two chrome steel balls (2 x 7 g), PTFE (1 equiv., 57 mg) and K_2SiO_3 (5.0 equiv., 443 mg). The jars were closed and securely fitted to a mill which was set for 3 h at a frequency of 35 Hz. Upon completion, the jars were opened, the powder was collected and extracted with H_2O (20 mL). The resulting suspension was centrifuged for 30 min to eliminate water-insoluble carbon black. The resulting clear supernatant was decanted and concentrated under reduced pressure to obtain a solid residue, which was sonicated with MeOH (6 mL) for 30 min and centrifugated for another 30 min. The clear supernatant was decanted and concentrated under reduced pressure to give KF (108 mg) in 78% yield (95% purity determined by quantitative ^{19}F NMR spectroscopy).

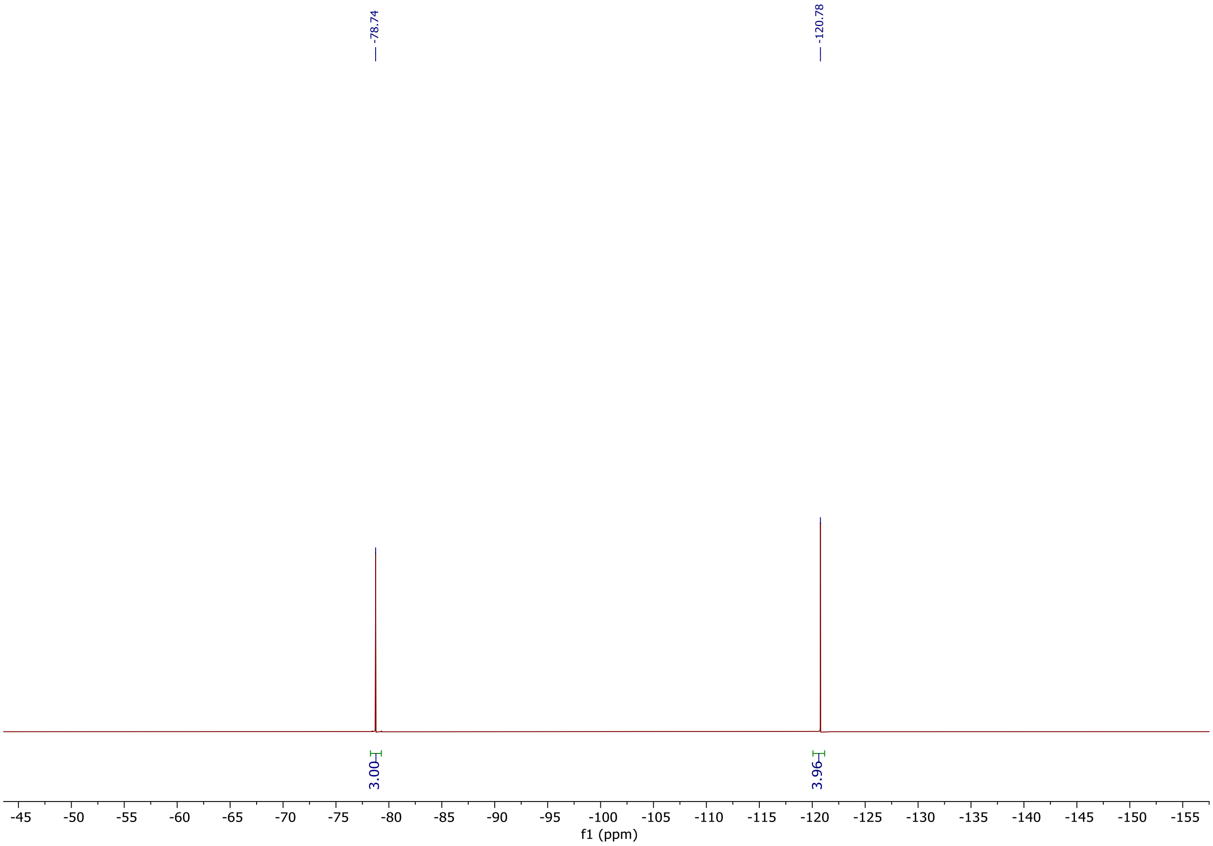

Figure S26: Quantitative ^{19}F NMR spectrum (377 MHz) of 14 mg KF^{PTFE} and 10 mg NaOTf (as an internal standard) in D_2O (10 atom% D).

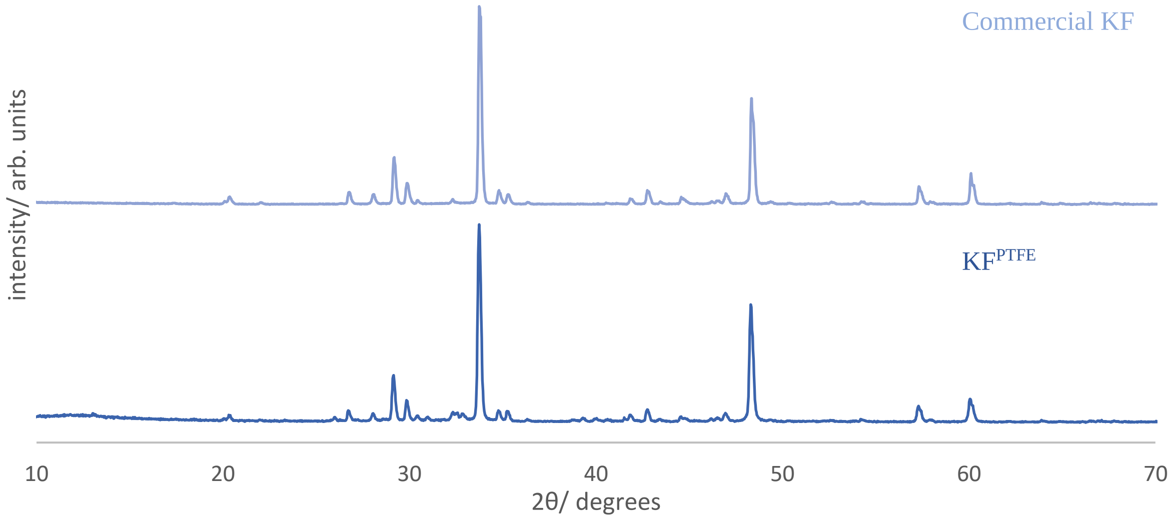

Figure S27: PXRD traces of commercial KF (top), and KF isolated from PTFE (bottom)

4.5 Synthesis of Tosyl Fluoride using NaF derived from PFAS-mix^{Si}

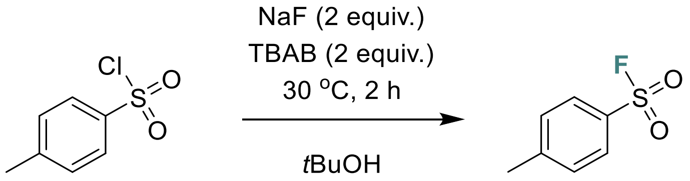

To a glass reaction vessel was added NaF^{PTFE} (1.0 mmol, 2.0 equiv.), tosyl chloride (0.5 mmol, 1.0 equiv), tetrabutylammonium bromide (1.0 mmol, 2.0 equiv.), and anhydrous *t*BuOH (2 mL). After stirring at 30 °C for 2 hours, the resulting suspension was cooled to room temperature, filtered through a short plug of silica gel (eluted with ~15 mL EtOAc) to remove insoluble by-products and the solvent was removed *in vacuo* to afford tosyl fluoride as a colourless oil that solidified immediately upon cooling below 40 °C (35 mg, 40% yield, *versus* 43% yield using commercial NaF).

^{1}H (400 MHz, $CDCl_3$) δ 7.91 (d, *J* = 8.6 Hz, 2H), 7.42 (d, *J* = 8.2 Hz, 2H), 2.49 (s, 3H)

^{13}C (101 MHz, $CDCl_3$) δ 146.9, 130.4, 130.3, 127.2, 22.0

^{19}F (377 MHz, $CDCl_3$) δ 62.7

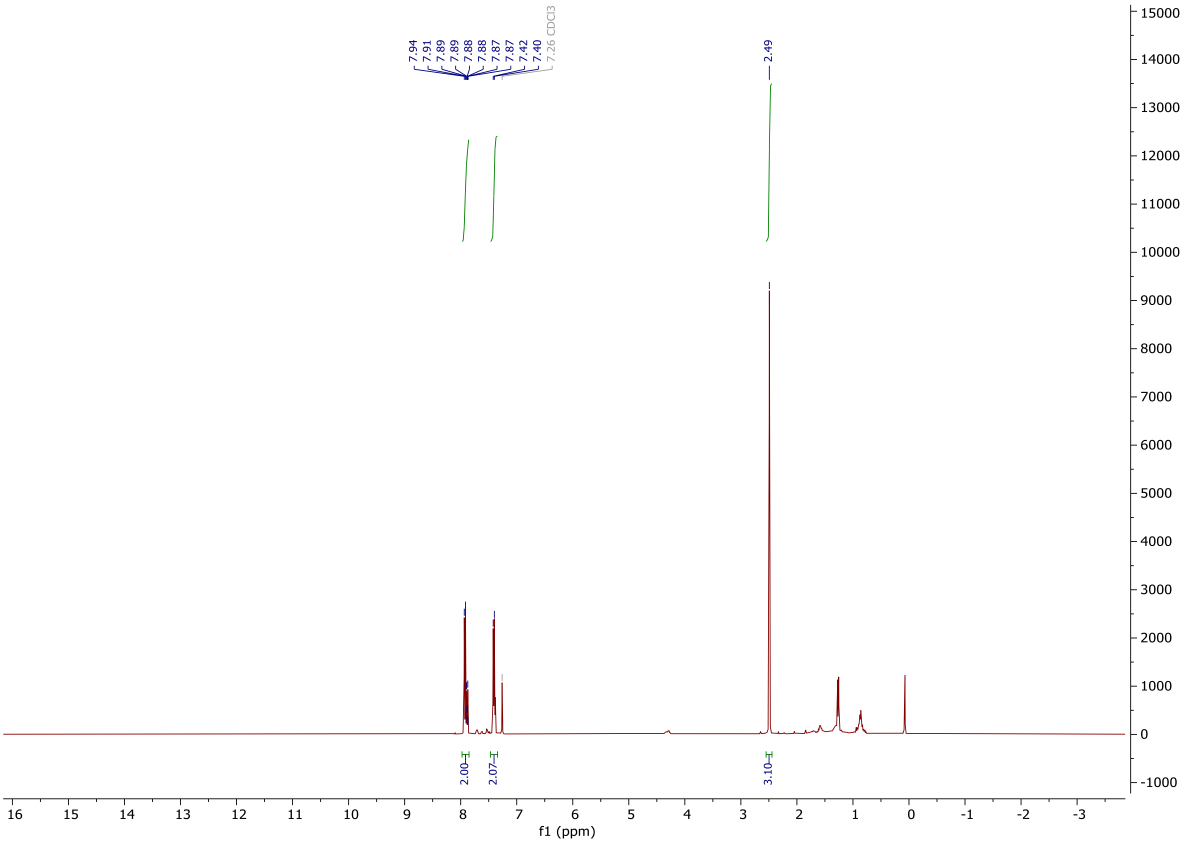

Figure S28: ^{1}H NMR spectrum (400 MHz, $CDCl_3$) of the tosyl fluoride prepared from NaF^{PTFE}.

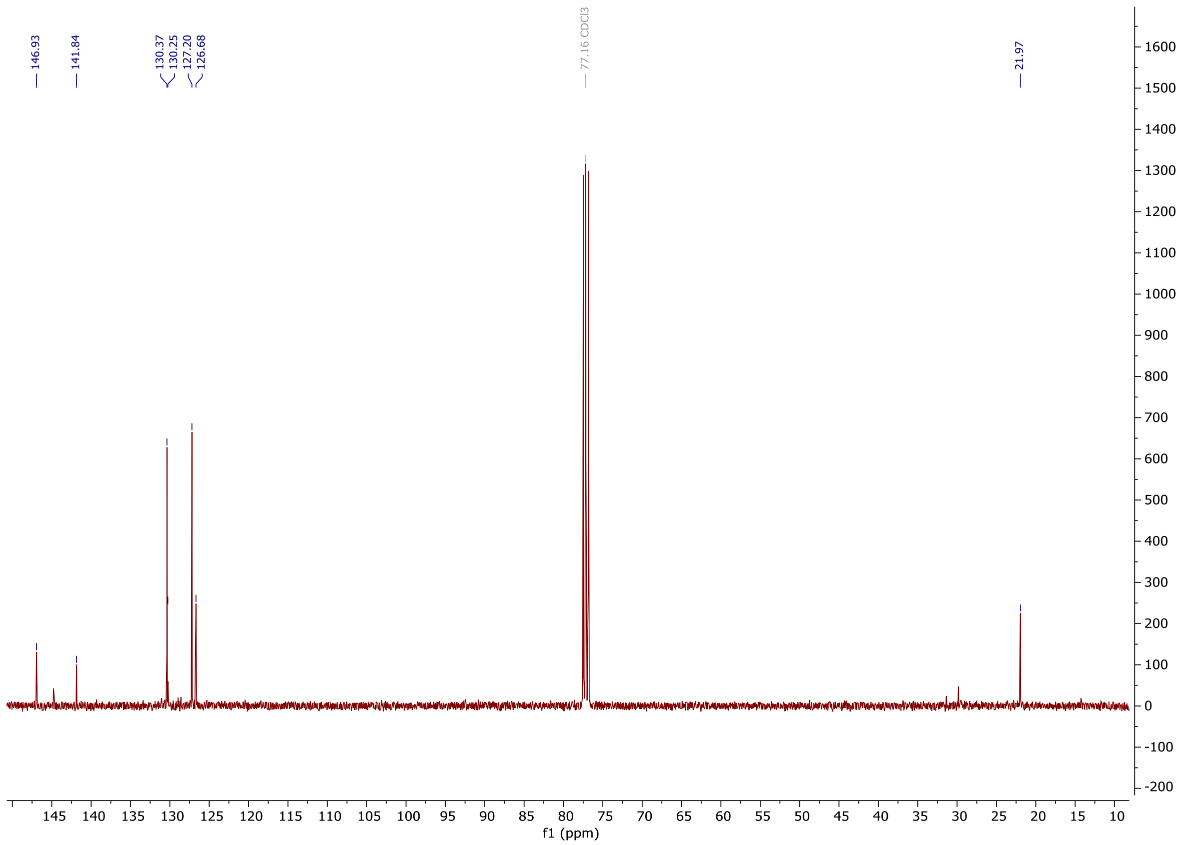

Figure S29: ^{13}C NMR spectrum (101 MHz, $CDCl_3$) of tosyl fluoride prepared from NaF^{PTFE}.

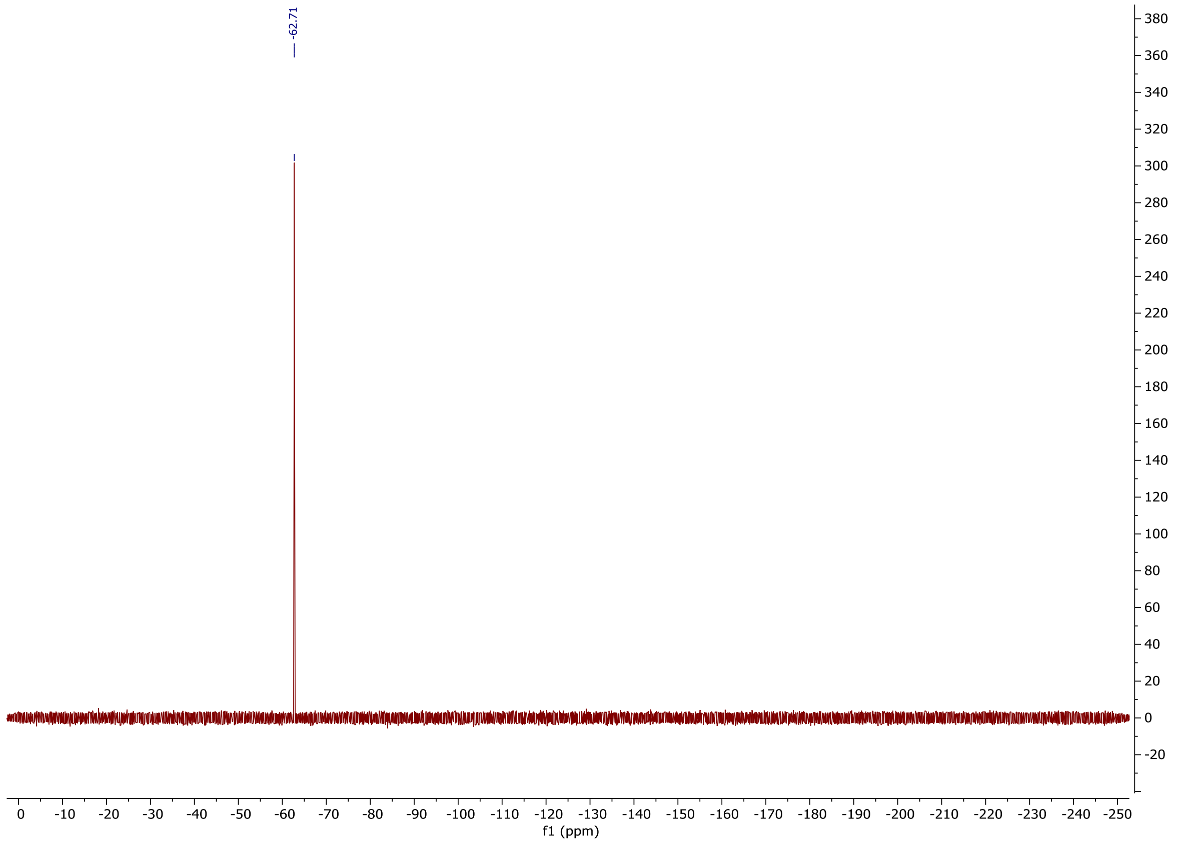

Figure S30: ^{19}F NMR spectrum (101 MHz, $CDCl_3$) of tosyl fluoride prepared from NaF^{PTFE}.

5. Photographic guide for experimental setup (ball milling)

Mechanochemical reactions were conducted using a Retsch MM400 mixer mill, or a Retsch MM500 Vario mixer mill.

Typical mechanochemistry setup:

1. PTFE and activator are added to stainless-steel 15 mL milling jar (equipped with rubber O-ring).
2. Two 7 g chrome steel balls are added.
3. Jar is closed, taped on the outside and securely fitted to the mixer mill.
4. Ball milling at 35 Hz for 3 h. *(In accordance with Verder's recommendations to extend the mill's lifetime, a milling program of 20 min milling followed by a 10 min pause was employed throughout the process, with a total effective milling time of 3 h. This intermittent milling protocol produced results identical to those obtained with continuous milling for 3 h.)*
5. Product material (solid powder) scraped from milling jar using a metal spatula.
6. Product material stored in borosilicate glass vial until further usage.

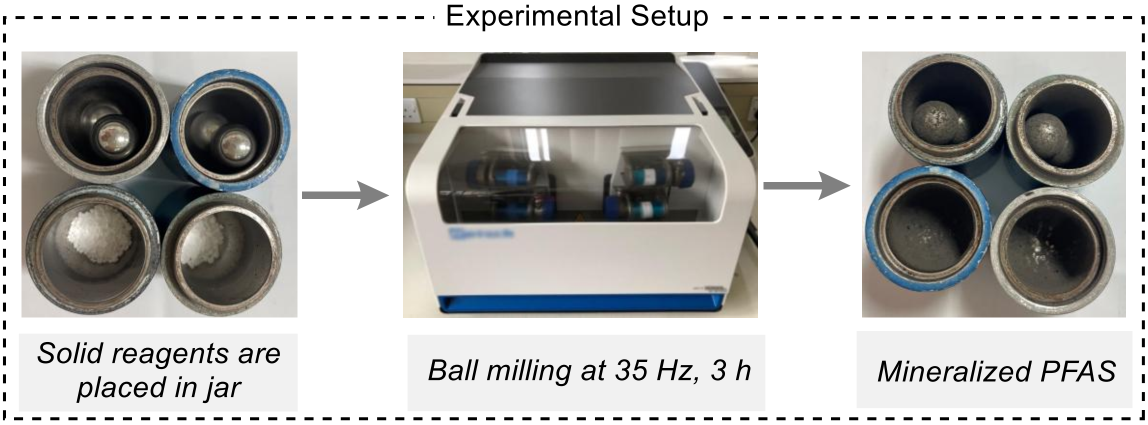

Figure S31. Photographic guide for ball milling of PTFE and Na_2SiO_3.

6. Mechanochemical destruction of various PFAS

6.1. General procedures

PFAS-mix^{Si} (GP): To a 15 mL stainless-steel milling jar was added two chrome steel balls (2 × 7 g), PFAS (1 eq.) and silicate (1.25 eq./F). The total material of the jar (PFAS and silicate) was kept constant at 500 mg. The jar was closed and securely fitted to the mill which was set for 3 h at a frequency of 35 Hz. Upon completion, the jar was opened and the powder was collected. An aliquot of PTFE-mix^{Si} (10-30 mg) and sodium triflate (10 mg, as internal standard) was extracted with D_2O (10 atom% D), centrifugated for 15-30 min and analysed by quantitative ^{19}F-NMR spectroscopy.

6.2. PFAS scope

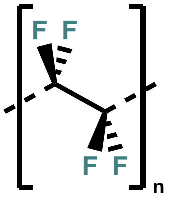

1, *PTFE (powder)*

PTFE-mix^{Si} was prepared according to GP using PTFE (1 eq., 70 mg) and Na_2SiO_3 (1.25 eq./F, 430 mg), and analysed by quantitative ^{19}F-NMR spectroscopy (quant. NaF, 98% NaF, quant. NaF).

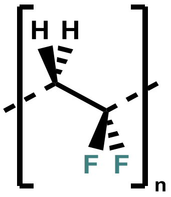

2, *PVDF*

PVDF-mix^{Si} was prepared according to GP using PVDF (1 eq., 87 mg) and Na_2SiO_3 (1.25 eq./F, 413 mg), and analysed by quantitative ^{19}F-NMR spectroscopy (91% NaF, 88% NaF, quant. NaF).

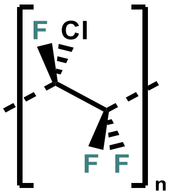

3, *PCTFE*

PCTFE-mix^{Si} was prepared according to GP using PCTFE (1 eq., 101 mg) and Na_2SiO_3 (1.25 eq./F, 399 mg), and analysed by quantitative ^{19}F-NMR spectroscopy (quant. NaF).

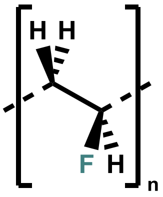

4, *PVF (film)*

PVF-mix^{Si} was prepared according to GP using PVF film (1 eq., 116 mg) and Na_2SiO_3 (1.25 eq./F, 384 mg), and analysed by quantitative ^{19}F-NMR spectroscopy (94% NaF).

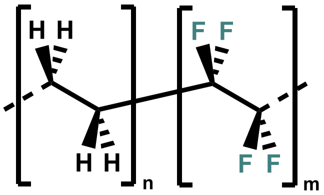

5, *ETFE*

ETFE-mix^{Si} was prepared according to GP using ETFE (1 eq., 87 mg) and Na_2SiO_3 (1.25 eq./F, 413 mg), and analysed by quantitative ^{19}F-NMR spectroscopy (85% NaF, 78% NaF, 85% NaF).

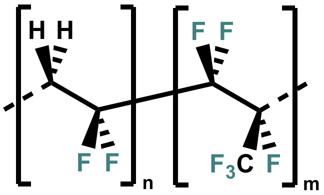

6, *PVDF-HFP*

PVDF-HFP-mix^{Si} was prepared according to GP using PVDF-HFP (1 eq., 75 mg) and Na_2SiO_3 (1.25 eq./F, 425 mg), and analysed by quantitative ^{19}F-NMR spectroscopy (87% NaF, 88% NaF, 84% NaF).

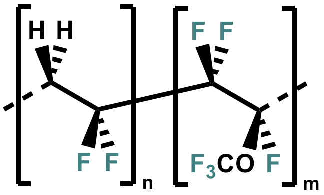

7, *PFA tubing*

PFA tubing-mix^{Si} was prepared according to GP using PFA tubing (1 eq., 74 mg) and Na_2SiO_3 (1.25 eq./F, 426 mg), and analysed by quantitative ^{19}F-NMR spectroscopy (quant. NaF).

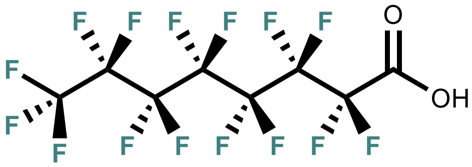

8, *PFOA*

PFOA-mix^{Si} was prepared according to GP using PFOA (1 eq., 77 mg) and Na_2SiO_3 (1.25 eq./F, 423 mg), and analysed by quantitative ^{19}F-NMR spectroscopy (88% NaF, 83% NaF, 94% NaF).

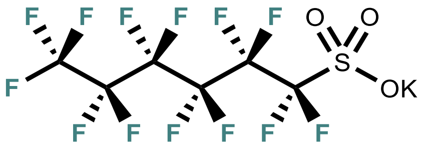

9, *KPFHxS*

KPFHxS-mix^{Si} was prepared according to GP using KPFHxS (1 eq., 91 mg) and Na_2SiO_3 (1.25 eq./F, 409 mg), and analysed by quantitative ^{19}F-NMR spectroscopy (75% NaF, 80% NaF, 74% NaF).

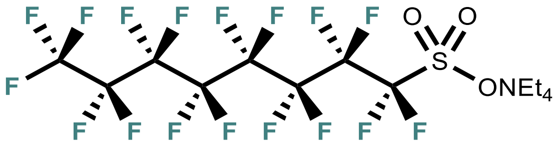

10, *PFOSNEt4*

PFOSNEt4-mix^{Si} was prepared according to GP using PFOSNEt4 (1 eq., 66 mg) and Na_2SiO_3 (2 eq./F, 434 mg) milling 6 h, and analysed by quantitative ^{19}F-NMR spectroscopy (quant. NaF).

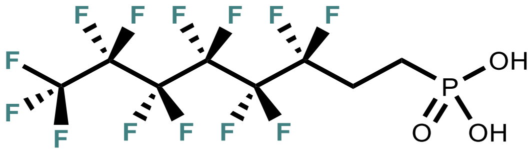

11, *6:2 FTPA*

6:2-FTPA-mix^{Si} was prepared according to GP using 6:2-FTPA (1 eq., 73 mg) and Na_2SiO_3 (1.25 eq./F, 427 mg), and analysed by quantitative ^{19}F-NMR spectroscopy (quant. NaF).

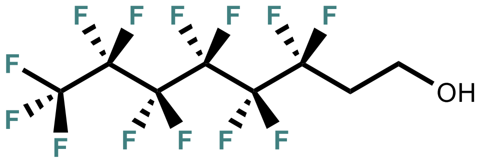

12, *6:2 FTOH*

6:2-FTOH-mix^{Si} was prepared according to GP using 6:2-FTOH (1 eq., 78 mg) and Na_2SiO_3 (1.25 eq./F, 422 mg), and analysed by quantitative ^{19}F-NMR spectroscopy (95% NaF, 99% NaF, quant. NaF).

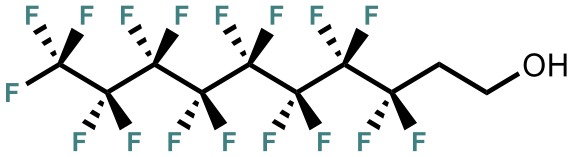

13, *8:2 FTOH*

8:2-FTOH-mix^{Si} was prepared according to GP using 8:2-FTOH (1 eq., 62 mg) and Na_2SiO_3 (1.25 eq./F, 438 mg), and analysed by quantitative ^{19}F-NMR spectroscopy (quant. NaF, quant. NaF, quant. NaF).

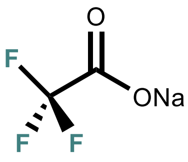

14, *CF_3CO_2Na*

TFA-Na-mix^{Si} was prepared according to GP using CF_3CO_2Na (1 eq., 115 mg) and Na_2SiO_3 (1.25 eq./F, 385 mg), and analysed by quantitative ^{19}F-NMR spectroscopy (99% NaF, 99% NaF, quant. NaF).

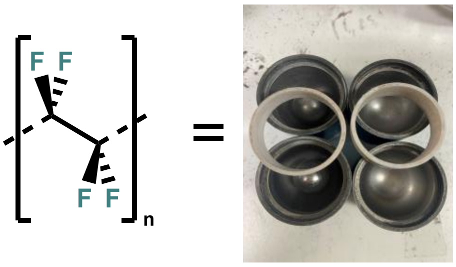

15, *PTFE (seal)*

PTFE seal-mix^{Si} was prepared according to GP using PTFE seal (1 eq., 70 mg) and Na_2SiO_3 (1.25 eq./F, 430 mg), and analysed by quantitative ^{19}F-NMR spectroscopy (quant. NaF).

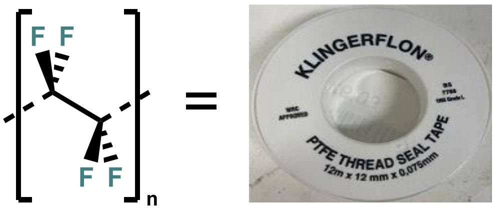

16, *PTFE (tape)*

PTFE tape-mix^{Si} was prepared according to GP using PTFE tape (1 eq., 70 mg) and Na_2SiO_3 (1.25 eq./F, 430 mg), and analysed by quantitative ^{19}F-NMR spectroscopy (quant. NaF).

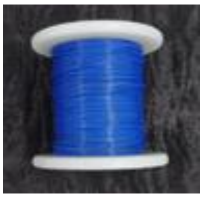

17, ETFE wire

ETFE wire-mix^{Si} was prepared according to GP using ETFE wire (1 eq., 87 mg) and Na_2SiO_3 (1.25 eq./F, 413 mg), and analysed by quantitative ^{19}F-NMR spectroscopy (quant. NaF).

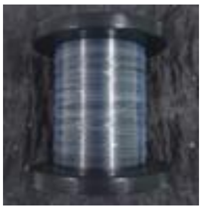

18, FEP tubing

FEP tubing-mix^{Si} was prepared according to GP using FEP tubing (1 eq., 70 mg) and Na_2SiO_3 (1.25 eq./F, 430 mg), and analysed by quantitative ^{19}F-NMR spectroscopy (quant. NaF).

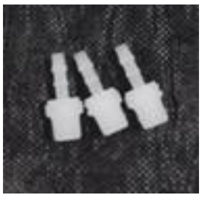

19, PVDF fitting

PVDF fitting-mix^{Si} was prepared according to GP using PVDF fitting (1 eq., 87 mg) and Na_2SiO_3 (1.25 eq./F, 413 mg), and analysed by quantitative ^{19}F-NMR spectroscopy (quant. NaF).

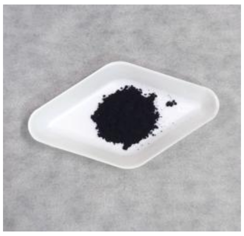

20, *PFOA (PAC-adsorbed)*

PFOA (200 mg) and Powered Activated Carbon (PAC) from Sigma Aldrich (1000 mg) were added to a round bottom flask and stirred in water (100 mL) at room temperature for 16 hours. The solid mixture was collected by vaccum filtration and dried under reduced pressure. The aqueous filtrate was concentrated and analyzed by ^{19}F NMR spectroscopy, which showed no residual PFOA, indicating that PFOA adsorption was quantitative.

PFOA PAC-adsorbed-mix^{Si} was prepared according to GP using PFOA PAC adsorbed (100 mg) and Na_2SiO_3 (400 mg), and analysed by quantitative ^{19}F-NMR spectroscopy (quant. NaF).

To determine the effect of water content on the efficiency of PFOA degradation, this protocol was repeated with varying amounts of water (as a mole percentage of the carbon sorbent) added to the reagents before milling.

Table S4. Effect of water content on milling efficacy.

Entry	Water content	F^{-} [a]
**1**	0.5 mol%	90%
**2**	5 mol%	81%
**3**	10 mol%	83%

[a] Fluoride release quantified as water soluble F^{-} determined by quantitative ^{19}F NMR spectroscopy, expressed as percent of the total fluorine content of PTFE.

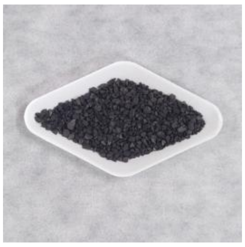

21, *8:2 FTOH (GAC-adsorbed)*

8:2 FTOH (200 mg) and Filtrasorb® 400 Granular Activated Carbon (GAC) from Calgon Carbon (1000 mg) were added to a round-bottom flask and stirred in water (100 mL) at room temperature for 16 hours. The solid mixture was collected by vaccum filtration and dried under reduced pressure. The aqueous filtrate was concentrated and analyzed by ^{19}F NMR spectroscopy, which showed no residual PFOA, indicating that PFOA adsorption was quantitative.

8:2 FTOH GAC-adsorbed-mix^{Si} was prepared according to GP using 8:2 FTOH GAC-adsorbed (100 mg) and Na_2SiO_3 (400 mg), and analysed by quantitative ^{19}F-NMR spectroscopy (quant. NaF).

7. Thermochemistry

Ideal reaction equations were deduced from the profile of product mixture assuming only one major pathway being present. Standard enthalpy change of reaction was calculated from the standard enthalpy of formation of the corresponding substances in the reaction equation, where cr = crystalline.[5]

	ΔH^o_f kcal/mol
PTFE (s)	-197.8 ± 0.4 kcal/(gfw C_2F_4)[5a]
Na_2SiO_3 (cr) [5c]	-371.6
NaF (cr) [5c]	-137.1
Na_2CO_3 (cr) [5c]	-270.2
SiO_2 (α quartz) (cr) [5c]	-217.7
SiO_2 (α cristobalite) (cr) [5c]	-217.4
SiO_2 (α tridymite) (cr) [5c]	-217.3

Reactants	Products	ΔH^o kcal/mol
$[C_2F_4]_n + 3n\ Na_2SiO_3$	$n\ C + n\ Na_2CO_3 + 4n\ NaF + 3n\ SiO_2$	-158.5

Table S5. Thermochemistry data.

8. Computational Studies

Computational Methods

Geometry optimisation, vibrational frequency and single point energy calculations were performed using *ORCA 6.1.1* software package.[6] Geometry optimization and frequency calculations were performed at the *ω*B97X-D4/def2-TZVP level of theory, in gas phase (g) or using an infinite dielectric constant (cr) under the conductor-like polarizable continuum model (CPCM).[7–10] Ground state geometries were identified by the absence of imaginary frequency vibrational modes. Thermochemical data were calculated at 298.15 K. Single point energy calculations were performed at the *ω*B97X-D4/ma-def2-TZVPP level of theory. [8–10] Conformational sampling was performed prior to geometry optimization of key structures using a Global Optimizer Algorithm (GOAT) featured in ORCA 6.1.1, based on the GFN2-XTB method.[11,12]

Assessment of the kinetic feasibility

A putative nucleophilic substitution reaction between various activators and a model PFAS perfluorobutane was examined in both gas phase and under a polar crystal environment (Figure S32). Nucleophiles include a sodium cyclic silicate trimer and a cluster containing three units of sodium phosphate. The computed activation barriers for silicate were 42.2 kcal/mol in gas phase and 47.1 kcal/mol under a polar crystal environment, respectively, demonstrating the kinetic feasibility of sodium silicate as the activator for PFAS degradation. The activation barriers are higher for sodium phosphate in both phases compared to sodium silicate ($\Delta\Delta G_g^‡$ = 6.5 kcal/mol and $\Delta\Delta G_{cr}^‡$ = 3.3 kcal/mol). The kinetic observation also correlates with the experimental data that sodium silicate is a superior activator than sodium phosphate. As a comparison, the weakest homolytic bond dissociation of perfluorobutane is 103 kcal/mol and the average C–C bond energy in PTFE is 90 kcal/mol.[13–15]

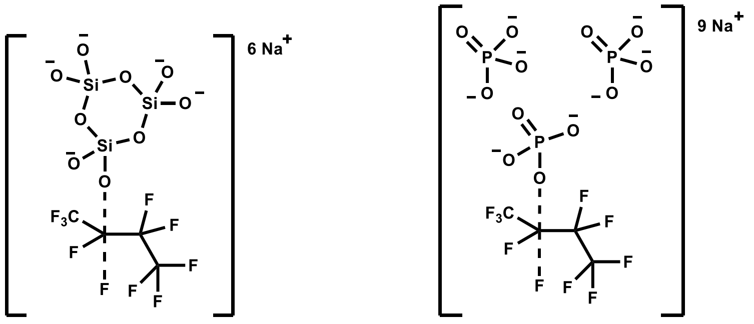

(i) $\Delta G_g^‡$ = 42.2 kcal/mol
$\Delta G_{cr}^‡$ = 47.1 kcal/mol
TS1

(ii) $\Delta G_g^‡$ = 48.7 kcal/mol
$\Delta G_{cr}^‡$ = 50.4 kcal/mol
TS2

Figure S32. Computed activation barriers of nucleophilic substitution between various activators, including (i) a sodium cyclic silicate trimer and (ii) a cluster containing three units of sodium phosphate, and perfluorobutane, modeled in gas phase (g) or in a polar crystal environment (cr).

Various modes of nucleophilic substitution between silicate trimer and perfluorobutane were compared in gas phase. Nucleophilic attacked at terminal carbon (**TS3**) resulted in higher action barrier than at internal carbon (**TS1**), $\Delta\Delta G^{\ddagger}$ = 11.3 kcal/mol). Internal nucleophilic substitution (S_Ni, **TS4**) is unfavored compared to S_N2 ($\Delta\Delta G^{\ddagger}$ = 12.6 kcal/mol). Introducing a second unit of sodium silicate trimer acting as the fluoride acceptor (TS5) does not lower the activation barrier ($\Delta\Delta G^{\ddagger}$ = 0.7 kcal/mol), likely due to the distortion of the accepting silicate trimer ($\Delta\Delta E_{dist}$ = 58.8 kcal/mol) outweighing the favored interaction between sodium and fluorine atom.

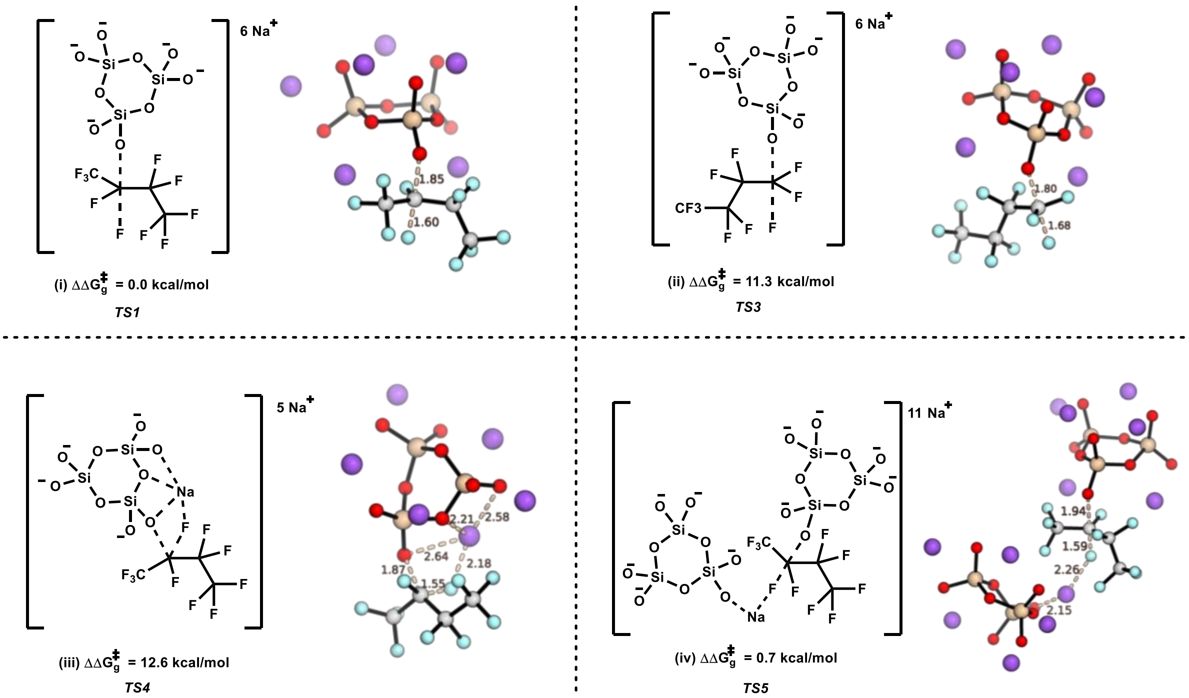

Figure S33. Computed relative activation barriers of nucleophilic substitution between silicate trimer and perfluorobutane in gas phase, including (i) S_N2 internal attack, (ii) S_N2 terminal attack, (iii) S_Ni internal attack assisted by Na^+, and (iv) S_N2 internal attack assisted by Na^+.

XYZ Coordinates

$(Na_2SiO_3)_3$ (g)

Si	-6.78623102	4.57086446	2.11601730
Si	-7.59825313	3.55085940	-0.54322414
Si	-7.72574005	1.69238138	1.82229975
O	-7.47429940	1.98258917	0.19756187
O	-6.61936779	4.46673483	0.46602008
O	-7.64283863	3.19177229	2.52781897
O	-9.12085433	4.04809125	-0.33585452
O	-6.95749802	3.33308123	-1.97074060
O	-6.56618296	0.74438391	2.45171561
O	-9.18413704	1.01537794	1.99134019
O	-7.58154948	5.93482091	2.47022334
O	-5.37322987	4.51249357	2.91177590
Na	-5.85899688	6.50042899	3.67035349
Na	-6.08021977	1.48769732	-1.44185785
Na	-8.82742829	6.01216832	0.65984215
Na	-5.24114498	2.35327322	3.20919732
Na	-10.15083842	2.25361724	0.43698925
Na	-8.20206878	-0.61446762	3.02289093

$(Na_2SiO_3)_3$ (cr)

Si	-6.76286549	4.59560290	2.11138684
Si	-7.58362340	3.55584831	-0.54803742
Si	-7.69552544	1.70546454	1.81717223
O	-7.47161199	2.01663898	0.18291723
O	-6.64696245	4.53062923	0.44269865
O	-7.61800957	3.21663810	2.51938207
O	-9.11322144	4.04946426	-0.43872557
O	-6.90592609	3.36218838	-1.97761462
O	-6.52629891	0.78106881	2.42593929
O	-9.15849356	1.06214300	2.01181678
O	-7.58976693	5.91682141	2.52182287
O	-5.31997155	4.52843661	2.82770193
Na	-5.83305246	6.49010173	3.78836449
Na	-6.28359992	1.30542138	-1.57963981
Na	-8.86884678	5.98058719	0.69204413
Na	-5.32544945	2.37745883	3.45511553
Na	-10.13831764	2.21518792	0.36977145
Na	-8.14933576	-0.65353377	3.04025296

$(Na_3PO_4)_3$ (g)

P	-2.14435508	1.61857680	-0.11251675
O	-3.35523685	0.60224238	-0.14048715
O	-1.31320150	1.42007298	-1.39509989
O	-1.29598571	1.30396195	1.14306509
O	-2.67599540	3.06273372	-0.02475033
Na	-4.69730157	2.36434056	-0.18247062
Na	2.39884876	-1.78043667	-0.22068594
Na	0.81692000	0.73366473	-1.54614792
P	3.05391684	1.00927393	0.14870873
O	1.76184799	1.88713213	0.18374782
O	4.33123469	1.83827494	0.24970749
O	3.06557865	0.28502795	-1.28175069
O	2.90015694	-0.10486814	1.19753705
Na	-1.06903852	-4.80913218	-0.20879285
Na	5.01732638	1.20363566	-1.65594363
Na	0.01127674	3.10289133	0.43777806
P	-0.32962474	-2.31185457	0.29421508
O	-0.42597669	-1.79040441	1.73162994
O	-1.77651808	-2.72089635	-0.18707891
O	0.62016294	-3.52417885	0.15341038
O	0.17161967	-1.18519241	-0.64630922
Na	-1.96393446	-0.81084126	-1.49205451
Na	-2.37017039	-0.73032174	1.52517267
Na	0.68459742	0.20306053	1.57058510

$(Na_3PO_4)_3$ (cr)

P	-2.19123658	1.71244389	-0.04349324
O	-3.19823029	0.51163932	-0.11125057
O	-1.21956755	1.64659215	-1.24940305
O	-1.36042113	1.57843202	1.26553453
O	-2.99993554	3.02043048	-0.05780177
Na	-4.92359153	1.92903556	-0.27095210
Na	2.37204699	-1.85249833	-0.27984620
Na	0.83894363	0.72948442	-1.61474088
P	3.08868174	0.90258396	0.14307335
O	1.85799670	1.85802898	0.16309833
O	4.41539256	1.67075168	0.25547209
O	3.07579776	0.16029471	-1.24693351
O	2.89535849	-0.15548356	1.25131004
Na	-0.84094641	-4.69864399	-0.74587239
Na	5.06696740	0.99059285	-1.76090649
Na	0.12340083	3.13585986	0.28318925
P	-0.36050576	-2.25854010	0.38975006
O	-0.52682516	-1.73866322	1.83300948
O	-1.72850606	-2.72560528	-0.18521309
O	0.66245060	-3.41838955	0.29591450
O	0.15171631	-1.08360683	-0.49586893
Na	-1.94861325	-0.79198203	-1.47650910
Na	-2.41598076	-0.54300055	1.73954963
Na	0.58175501	0.28700654	1.46035905

C_4H_{10} (g)

C	0.58391531	0.51599088	-0.09664554
F	0.57094261	1.17901820	-1.26489636
F	0.40576363	1.39474945	0.90701576
C	1.99837840	-0.11766992	0.07695406
F	2.15550888	-0.59844973	1.30726082
F	2.91647114	0.82435846	-0.13270651
F	2.18075477	-1.10147690	-0.80325074
C	-0.58392139	-0.51600408	-0.09669413
F	-0.57090405	-1.17903943	-1.26492896
F	-0.40582541	-1.39475786	0.90699447
C	-1.99837651	0.11767338	0.07693305
F	-2.18076454	1.10151969	-0.80322617
F	-2.15545142	0.59842283	1.30726379
F	-2.91649240	-0.82433296	-0.13270955

C_4H_{10} (cr)

C	0.58461326	0.51582290	-0.09222429
F	0.56757536	1.18321723	-1.25844423
F	0.41038516	1.39210735	0.91398162
C	2.00060409	-0.11854364	0.07389917
F	2.15840076	-0.61385472	1.29865260
F	2.91762241	0.82734686	-0.12453457
F	2.18517722	-1.09130729	-0.81776024
C	-0.58463091	-0.51587957	-0.09215859
F	-0.56761060	-1.18332924	-1.25836487
F	-0.41040804	-1.39211826	0.91407212
C	-2.00061021	0.11854634	0.07383246
F	-2.18502823	1.09149687	-0.81762353
F	-2.15840421	0.61374187	1.29868144
F	-2.91768705	-0.82724469	-0.12464509

TS1 (g)

C	-4.33845639	-1.06469884	-0.56330442
F	-5.27340045	-0.25266383	-0.09352428
F	-4.77993040	-2.32537712	-0.39729208
C	-2.44936486	1.45229905	1.38933060
F	-2.01750590	1.17890965	2.60185133
F	-3.74043274	1.75005107	1.52387861
F	-1.79778152	2.60075830	0.99678388

C	-2.97790951	-0.95499066	0.20886132
F	-2.20178444	-1.89173242	-0.41783180
F	-3.25287625	-1.35318498	1.45423734
C	-2.21160041	0.40510803	0.22573323
F	-4.18265706	-0.88088893	-1.87817678
F	-3.23506765	1.19681227	-0.71690464
F	-1.22669575	0.60631937	-0.74535135
Si	0.65377664	-0.58985197	0.97442263
Si	2.14606976	-1.33090081	-1.61826736
Si	2.56752753	1.39733848	-0.32214047
O	2.62120475	0.30697839	-1.54105310
O	0.88977748	-1.41604445	-0.44718988
O	1.30134552	0.95774199	0.68193555
O	3.38410439	-2.13392829	-0.97024364
O	1.42983485	-1.67042132	-2.97126658
O	2.26168714	2.90795779	-0.77174712
O	3.97571385	1.30981220	0.51973287
O	1.59425291	-1.25712918	2.08523982
O	-0.91345151	-0.43081361	1.24611583
Na	0.07860830	2.68190983	-0.44895005
Na	4.19873081	3.49354724	-0.00834664
Na	-0.57740857	-1.46816454	-2.18346687
Na	2.31528068	-2.92511863	0.78620248
Na	2.96657493	0.40232216	2.36892229
Na	4.73265086	-0.45745927	-0.77019676

TS1 (cr)

C	-4.87473114	-0.60681938	-0.40204735
F	-5.19764834	-0.19060078	-1.62397218
F	-5.37136003	0.22383056	0.51010255
C	-1.92096442	1.18516465	0.89877025
F	-0.99502631	0.71149518	1.74368772
F	-2.97717380	1.45284185	1.65683752
F	-1.40195321	2.34980062	0.43540457
C	-3.33624932	-0.86941239	-0.23207809
F	-3.07034833	-1.79307359	-1.17487567
F	-3.23839363	-1.41724367	0.99931071
C	-2.31392244	0.31122437	-0.35386941
F	-5.48363625	-1.79628511	-0.22088599
F	-3.46574156	1.45189189	-0.70486388
F	-1.72042904	0.55630306	-1.54097822
Si	0.58794417	-1.01183574	-0.33287712
Si	2.55863556	1.17557684	-1.11512991
Si	2.93709313	-0.38523644	1.50551947
O	3.25631659	0.79527809	0.35918050
O	1.04513580	0.40706336	-1.09281954
O	1.39880403	-0.99242754	1.13959588
O	3.46533711	0.44540579	-2.21440944
O	2.21318939	2.73080080	-1.08324260
O	2.73385340	0.41405074	2.87598808
O	3.99018413	-1.55537466	1.22864818
O	1.17702143	-2.24568244	-1.16572624
O	-0.98401754	-0.97157008	-0.01819620
Na	0.62428599	-0.28315631	3.14586481
Na	2.75701032	2.53077881	1.63449906
Na	0.03497045	2.42904960	-1.49323515
Na	2.07347099	-1.16558424	-2.90421756
Na	2.55131025	-3.09606716	0.40662759
Na	4.88784962	-0.54568970	-0.66861835

TS2 (g)

C	3.09762298	0.28458191	0.13624303
F	3.44986103	0.75773153	-1.04749402
F	3.21567009	-1.03256083	0.17992199
C	-0.31847763	-0.96205262	0.18924001
F	-1.10667018	-1.30765398	-0.81400192
F	-1.10630008	-0.99938822	1.31742122
F	0.58232958	-1.91145562	0.42742156
C	1.69876104	0.81415109	0.60780856
F	1.82910969	2.14727989	0.61727607
F	1.61807159	0.37532576	1.92256893
C	0.34454427	0.44446432	-0.08830140
F	3.98475255	0.78258988	1.03427232
F	1.23384217	-0.44547523	-1.34141215
F	-0.08684622	1.23353055	-1.11105855
P	-2.02408086	1.86820571	1.06438881
O	-2.49339667	1.52581288	2.51471796
O	-2.05722631	3.36634476	0.83132748
O	-2.84149184	1.07234508	0.04582279
O	-0.50627556	1.35440265	1.06136819
Na	-0.54145652	0.61223439	3.13635124
Na	-6.90107111	4.30993847	-0.64730810
Na	-3.62014239	4.48187554	-0.43923345
P	-5.06356729	3.91707545	-2.81259770
O	-3.76808429	3.19718238	-2.30801148
O	-4.99597403	4.27827955	-4.28991713
O	-5.15478809	5.28312625	-1.98280623
O	-6.26178454	3.05446938	-2.38681824
Na	-7.95164988	3.12398352	3.52767326
Na	-5.14368185	6.36207835	-3.90285800
Na	-2.07567148	2.01759472	-1.91878735
P	-6.22529378	2.65594748	1.55543769
O	-6.16402511	1.24337107	0.95402405
O	-5.85665206	2.59499599	3.07956592
O	-7.61489992	3.31717039	1.40069869
O	-5.16773261	3.54928378	0.84802978
Na	-3.76022472	3.46349459	2.64780124
Na	-4.35943655	0.37177831	1.80237492
Na	-5.07506038	1.71553751	-0.99169411

TS2 (cr)

C	3.11406143	0.15431868	0.15340160
F	3.45275113	0.46300607	-1.09348260
F	3.21290602	-1.15408593	0.35080198
C	-0.36106573	-1.01147881	0.39361273
F	-1.13666057	-1.48134739	-0.57654129
F	-1.14993577	-0.86768557	1.48171396
F	0.52380384	-1.94558919	0.75687343
C	1.72305687	0.75119048	0.56611798
F	1.87248935	2.07381883	0.38430672
F	1.64463435	0.48498616	1.90607115
C	0.38040835	0.29387928	-0.10413709
F	4.02152638	0.74165340	0.96130015
F	1.20964464	-0.74197741	-1.21962307
F	-0.07234921	0.98272261	-1.17179679
P	-1.94957657	1.98748477	0.88832857
O	-2.48480532	1.86819281	2.33841953
O	-1.88021747	3.42459173	0.38034411
O	-2.76840420	1.11023890	-0.06611550
O	-0.46692035	1.41969896	1.00773399
Na	-0.56139091	1.05396344	3.21080589
Na	-6.94188163	4.29981987	-0.55306442
Na	-3.68739274	4.60992792	-0.36397874
P	-5.13479226	4.04911633	-2.76986270
O	-3.77569492	3.38953807	-2.38868302
O	-5.15927186	4.51108488	-4.23493882
O	-5.30493166	5.32448237	-1.85887994
O	-6.26244405	3.05678294	-2.41221470
Na	-7.93352635	3.14472125	3.47849658
Na	-5.41950434	6.64042816	-3.63995307
Na	-1.89639901	2.49224228	-1.85719709
P	-6.22292515	2.42325940	1.45888146
O	-6.06646981	1.04385187	0.78928480
O	-5.90804903	2.31710913	2.98121908

O	-7.64108622	3.01196272	1.27285511
O	-5.18574103	3.40292289	0.82398885
Na	-3.96151721	3.47261139	2.81701956
Na	-4.16293837	0.35968204	1.75265296
Na	-4.84678755	1.76047128	-1.02430544

TS3 (g)

C	2.01799030	-0.24985185	-0.03555679
F	3.06730329	0.48365792	0.35191814
F	2.06020986	-0.50201535	-1.35085709
C	2.23195354	-1.65458937	0.64643058
F	1.10808695	-2.41256216	0.58192402
F	2.57606871	-1.55554392	1.92907622
F	3.19386612	-2.31249213	0.01067340
C	0.65243020	0.41082554	0.38666728
F	0.54643985	0.04505948	1.69696747
F	-0.31783792	-0.26747884	-0.30197546
C	0.37595408	1.94450277	0.16751271
F	-0.50341885	2.41554489	-0.75308510
F	1.52716809	1.91632569	-1.05622313
F	1.04048644	2.81216155	0.89219597
Si	-2.51350499	1.51570132	1.28707562
Si	-4.19381104	0.31999163	-0.99961045
Si	-3.85061449	-1.19990486	1.61686877
O	-4.21922726	-1.04256311	0.03149104
O	-2.98027501	1.30640735	-0.29053973
O	-2.70477657	-0.03065336	1.98359271
O	-5.58701933	1.07213010	-0.69463554
O	-3.64039897	-0.01142882	-2.42825321
O	-3.17816818	-2.59497786	2.04379580
O	-5.21226208	-0.92980225	2.49746009
O	-3.59743023	2.45211505	2.00388869
O	-0.95925479	1.88424137	1.37082289
Na	-1.14099768	-1.76249581	1.96082538
Na	-4.89701193	-3.02337252	3.27866095
Na	-1.68006328	0.84614168	-2.14363811
Na	-4.74871610	2.91651830	0.15306228
Na	-4.44450801	1.02570344	3.38706894
Na	-6.43830731	-0.41507684	0.61338469

TS4 (g)

C	1.68256613	0.35772284	0.71618746
F	2.70077739	1.13956854	1.13603757
F	2.22813424	-0.58591648	-0.09064179
C	1.21162530	-0.44275838	1.98068944
F	0.40242095	-1.46292614	1.67233372
F	0.57464920	0.31482136	2.89419116
F	2.28546663	-0.96155910	2.58901999
C	0.51295022	1.18103863	-0.04693657
F	0.55382989	2.22807857	1.08954919
F	-0.12841518	-0.03764144	-0.46185333
C	1.06504400	1.91467360	-1.31355996
F	2.38434084	1.65591791	-1.47704215
F	0.95198815	3.23410517	-1.22474685
F	0.45816457	1.49428177	-2.41737045
Si	-3.25887943	0.21997886	2.75781562
Si	-2.49491379	1.12166831	0.00710594
Si	-5.11046735	1.94877266	1.17137737
O	-3.69053953	2.23660744	0.33906646
O	-2.15250864	0.50016327	1.53861254
O	-4.73439716	0.63040120	2.14268870
O	-1.16139141	1.96566519	-0.33047563
O	-3.06657941	-0.10456234	-0.83703933
O	-6.27268679	1.62234614	0.10583348
O	-5.53620422	3.17232428	2.13972173
O	-2.84620579	1.24219965	3.96738181
O	-3.08298199	-1.32650746	3.11962731
Na	-4.72853332	2.55546220	4.07214845
Na	-5.06299563	0.55874769	-1.35896630
Na	-2.00553753	-0.67371299	4.89657356
Na	-1.27009877	2.39245437	2.27503496
Na	-7.31981484	3.37289746	0.84666899
Na	-2.47582234	-1.64853126	0.81394369

TS5 (g)

C	-4.45232142	-1.81920219	-0.48575893
F	-5.56390865	-1.13147044	-0.25202382
F	-4.64106596	-3.06362225	-0.02060540
C	-3.16334397	1.46313709	0.87981333
F	-3.30866407	1.15624811	2.16037256
F	-4.30225800	2.04885925	0.51702687
F	-2.21081335	2.41940461	0.75683182
C	-3.19406302	-1.23528527	0.25609644
F	-2.19058953	-2.06768843	-0.15804682
F	-3.44079290	-1.43393415	1.54540040
C	-2.76017155	0.24340723	-0.04742709
F	-4.25375154	-1.92451175	-1.80595998
F	-3.83866094	0.51260214	-1.18206131
F	-1.75156145	0.43032426	-0.95992428
Si	0.21816330	-0.02423203	1.12808102
Si	2.06481920	-1.07700727	-1.10078033
Si	1.98755571	1.89484479	-0.46442731
O	2.30761619	0.58504816	-1.39195456
O	0.69109119	-1.08588535	-0.06426734
O	0.69855958	1.49607330	0.52908870
O	3.31239480	-1.50672274	-0.17659710
O	1.58001410	-1.83767179	-2.38470300
O	1.52248569	3.20797545	-1.26043379
O	3.30352533	2.20839165	0.46779441
O	1.13382367	-0.29029705	2.41602691
O	-1.37106898	-0.03970878	1.27007213
Na	-0.62055921	2.75584623	-0.92994811
Na	3.25784710	4.24792180	-0.51537309
Na	-0.50221175	-1.81886354	-1.85378401
Na	2.18607555	-2.07020516	1.61480571
Na	2.24620467	1.57824192	2.40072721
Na	4.42027863	0.34689663	-0.31035686
Si	-6.58067435	4.45192016	2.18989352
Si	-7.31566663	3.66910006	-0.58295700
Si	-7.75257943	1.68318959	1.64676821
O	-7.38815286	2.06724302	0.06659560
O	-6.34163432	4.44923385	0.54966012
O	-7.52871069	3.10255044	2.48367754
O	-8.80323036	4.29106282	-0.44172621
O	-6.58833137	3.48836823	-1.97158222
O	-6.74127344	0.57983422	2.26829582
O	-9.28271300	1.15635885	1.69014631
O	-7.31751763	5.83553914	2.60577778
O	-5.22811000	4.24896103	3.06448193
Na	-5.72162302	6.12534454	4.04166391
Na	-5.83176183	1.53603339	-1.47200350
Na	-8.40655845	6.14209510	0.71914907
Na	-5.42606115	2.05916545	3.27687447
Na	-10.01964738	2.51279673	0.10332942
Na	-8.49607725	-0.67215135	2.57576045

9. References

1. Brouwer, D. H.; Horvath, M. Minimizing the effects of RF inhomogeneity and phase transients allows resolution of two peaks in the ^{1}H CRAMPS NMR spectrum of adamantane. *Solid State Nuclear Magnetic Resonance* **2015**, *71*, 30–40.
2. Griffin, J. M.; Yates, J. R.; Berry, A. J.; Wimperis, S.; Ashbrook, S. E. High-Resolution ^{19}F MAS NMR Spectroscopy: Structural Disorder and Unusual J Couplings in a Fluorinated Hydroxy-Silicate. *J. Am. Chem. Soc.* **2010**, *132*, 15651–15660.
3. Sherriff, B. L.; Grundy, H. D.; Hartman J. S. The relationship between ^{29}Si MAS NMR chemical shift and silicate mineral structure. *Eur. J. Mineral.* **1991**, *3*, 751–768.
4. Miller, J. M. Fluorine-19 magic-angle spinning NMR, *Progress in Nuclear Magnetic Resonance Spectroscopy* **1996**, *28*, 255–281.
5. (a) The Heats of Combustion of Polytetrafluoroethylene (Teflon) and Graphite in Elemental Fluorine. J. Res. Natl. Bur. Stand. A. Phys. Chem. 71a, 105–118 (1967). (b) (i) A. Jain, S. P. Ong, G. Hautier, W. Chen, W. D. Richards, S. Dacek, S. Cholia, D. Gunter, D. Skinner, G. Ceder, K. A. Persson, Commentary: The Materials Project: A materials genome approach to accelerating aterials innovation. APL Materials 1, 011002 (2013). (b) A. Jain, G. Hautier, S. P. Ong, C. J. Moore, C. C. Fischer, K. A. Persson, G. Ceder, Formation enthalpies by mixing GGA and GGA + U calculations. Phys. Rev. B 84, 045115 (2011). (c) The NBS Tables of Chemical Thermodynamic Properties: Selected Values for Inorganic and C1 and C2 Organic Substances in SI Units, National Institute of Standards and Technology, https://doi.org/10.18434/M32124
6. (a) Neese, F. The ORCA program system. *WIREs Comput. Mol. Sci.* **2012**, *2* (1), 73–78; (b) Neese, F. Software update: The ORCA program system – Version 5.0. *WIREs Comput. Mol. Sci.* **2022**, *12* (5), e1606.
7. Chai, J.-D.; Head-Gordon, M. Long-range corrected hybrid density functionals with damped atom–atom dispersion corrections. *Phys. Chem. Chem. Phys.* **2008**, *10* (44), 6615–6620.
8. (a) Grimme, S.; Antony, J.; Ehrlich, S.; Krieg, H. A consistent and accurate *ab initio* parametrization of density functional dispersion correction (DFT-D) for the 94 elements H-Pu. *J. Chem. Phys.* **2010**, *132* (15), 154104; (b) Caldeweyher, E.; Ehlert, S.; Hansen, A.; Neugebauer, H.; Spicher, S.; Bannwarth, C.; Grimme, S. A generally applicable atomic-charge dependent London dispersion correction. *J. Chem. Phys.* **2019**, *150* (15).
9. Weigend, F.; Ahlrichs, R. Balanced basis sets of split valence, triple zeta valence and quadruple zeta valence quality for H to Rn: Design and assessment of accuracy. *Phys. Chem. Chem. Phys.* **2005**, *7* (18), 3297–3305.
10. Cossi, M.; Rega, N.; Scalmani, G.; Barone, V. Energies, structures, and electronic properties of molecules in solution with the C-PCM solvation model. *J. Comput. Chem.* **2003**, *24* (6), 669–681.
11. de Souza, B. GOAT: A global optimization algorithm for molecules and atomic clusters. *Angew. Chem. Int. Ed.* **2025**, *64* (18), e202500393.
12. Bannwarth, C.; Ehlert, S.; Grimme, S. GFN2-xTB—An accurate and broadly parametrized self-consistent tight-binding quantum chemical method with multipole electrostatics and density-dependent dispersion contributions. *J. Chem. Theory Comput.* **2019**, *15* (3), 1652–1671.
13. Dixon, D. A.; Smart, B. E.; Krusic, P. J.; Matsuzawa, N. Bond energies in organofluorine systems: applications to Teflon® and fullerenes. *J. Fluorine Chem.* **1995**, *72* (2), 209–214.

14. Giannetti, E. Thermal stability and bond dissociation energy of fluorinated polymers: A critical evaluation. *J. Fluorine Chem.* **2005**, *126* (4), 623–630.
15. Wu, E. C.; Rodgers, A. S. Kinetics of the gas phase reaction of pentafluoroethyl iodide with hydrogen iodide. Enthalpy of formation of the pentafluoroethyl radical and the .pi. bond dissociation energy in tetrafluoroethylene. *J. Am. Chem. Soc.* **1976**, *98* (20), 6112–6115.

10. PFAS-mix NMR characterization

n(OTf⁻)	m (sample)	Yield of F⁻ %
0.058 mmol	14 mg	quant.

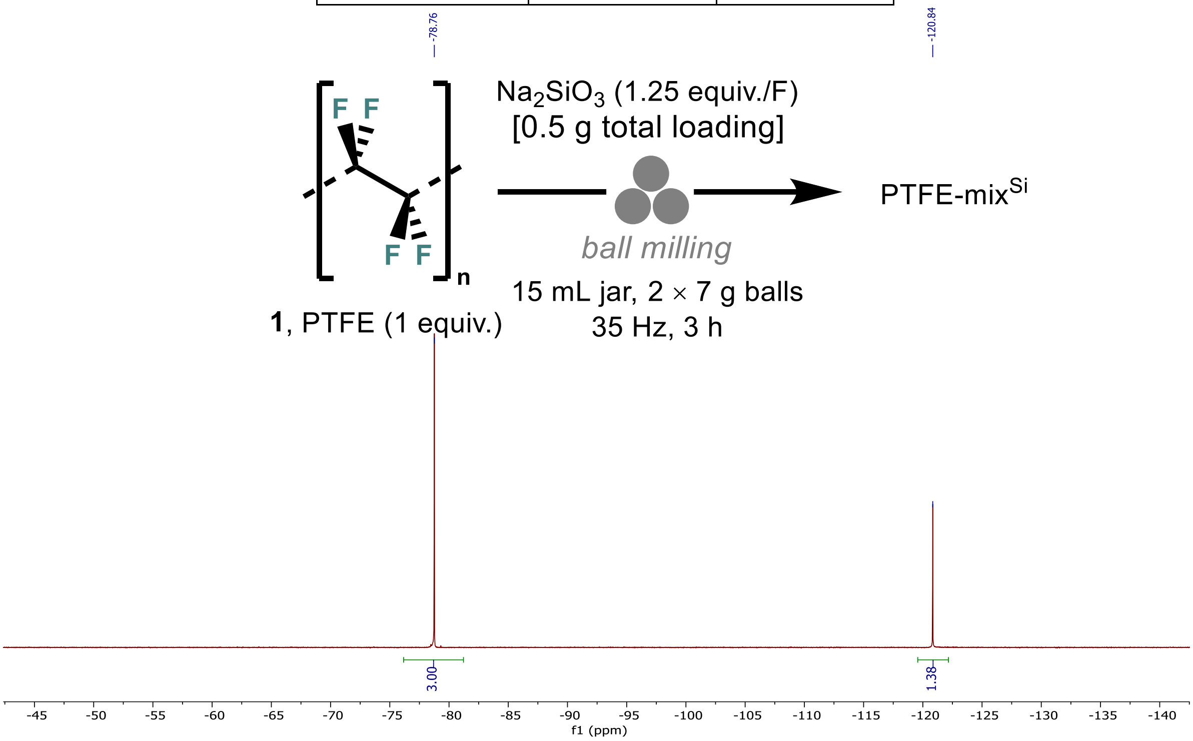

n(OTf⁻)	m (sample)	Yield of F⁻ %
0.044 mmol	15 mg	98%

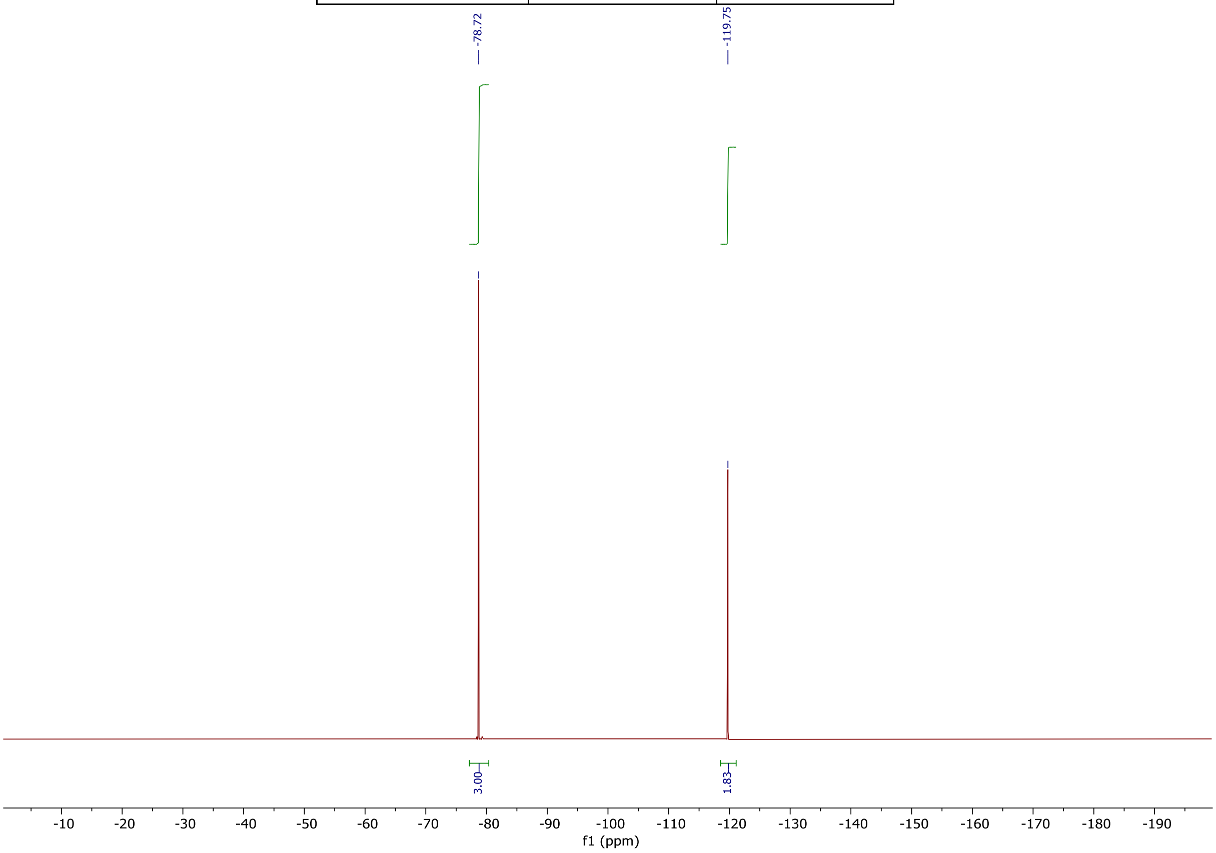

n(OTf⁻)	m (sample)	Yield of F⁻ %
0.099 mmol	22 mg	quant.

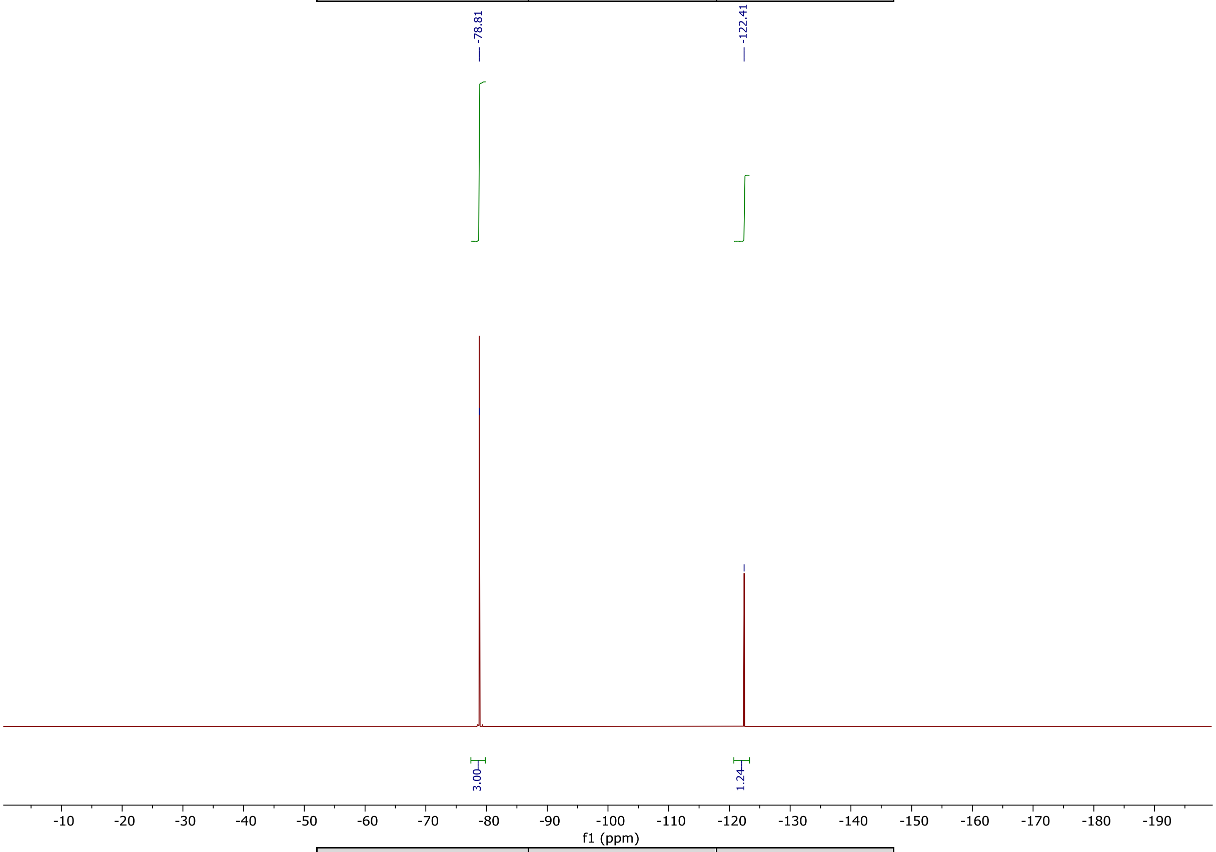

n(OTf⁻)	m (sample)	Yield of F⁻ %
0.058 mmol	10.5 mg	quant.

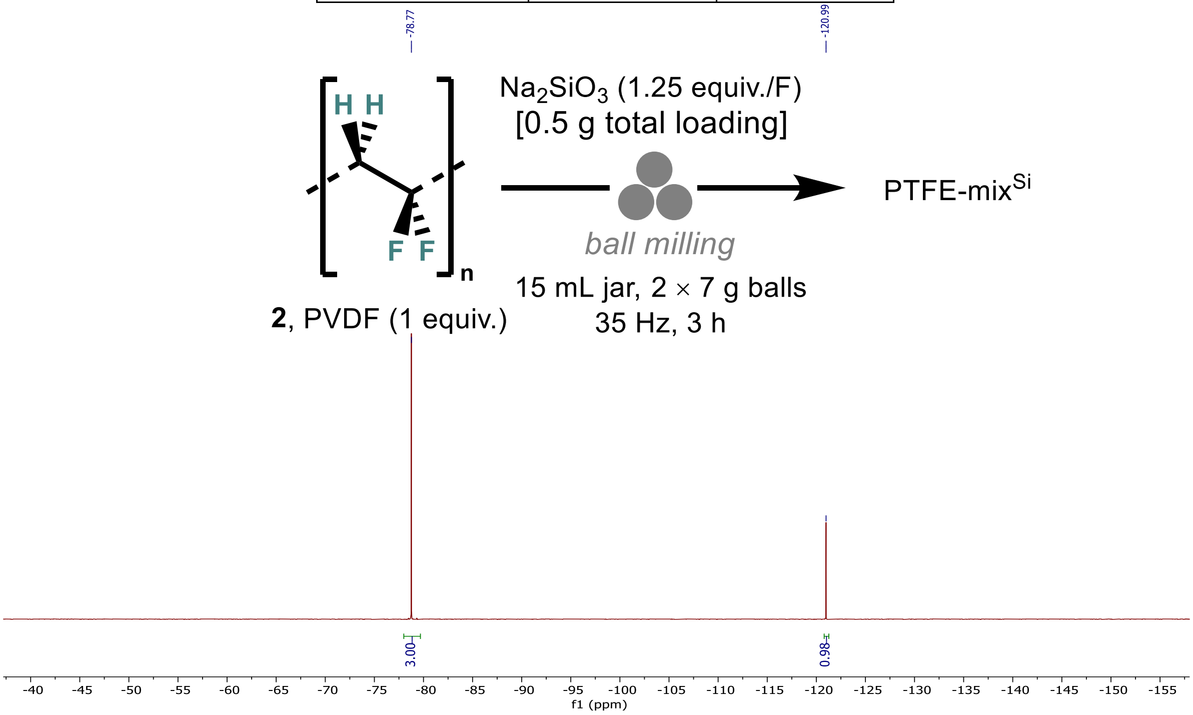

n(OTf⁻)	m (sample)	Yield of F⁻ %
0.081 mmol	26.0 mg	88%

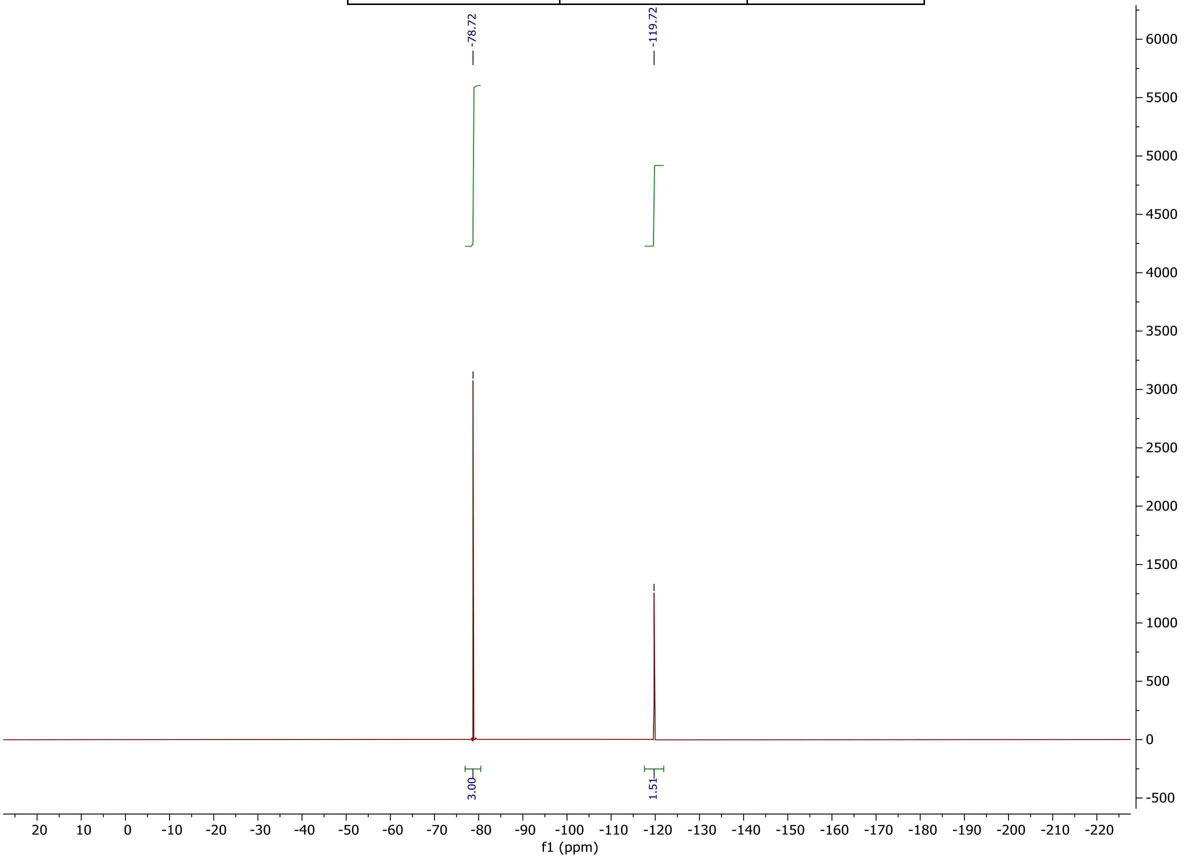

n(OTf⁻)	m (sample)	Yield of F⁻ %
0.069 mmol	29.5 mg	91%

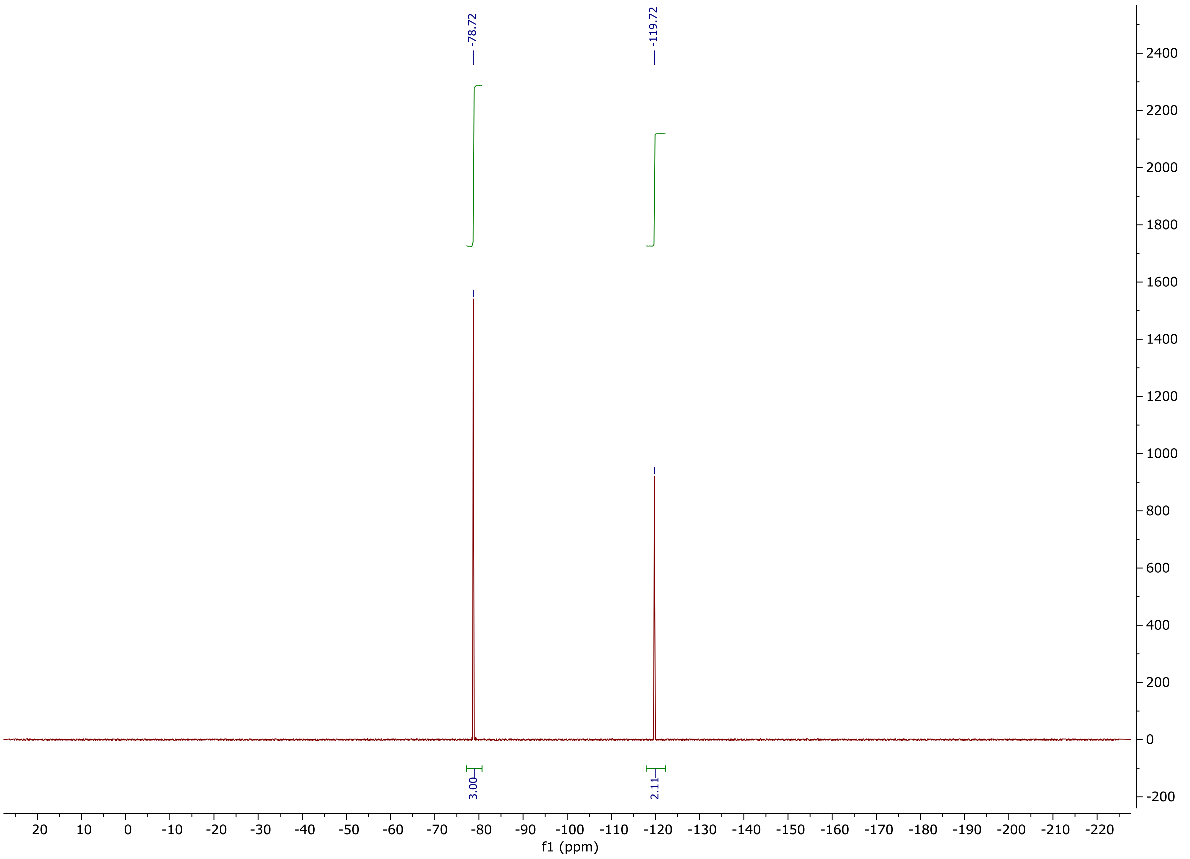

n(OTf⁻)	m (sample)	Yield of F⁻ %
0.058 mmol	19 mg	quant.

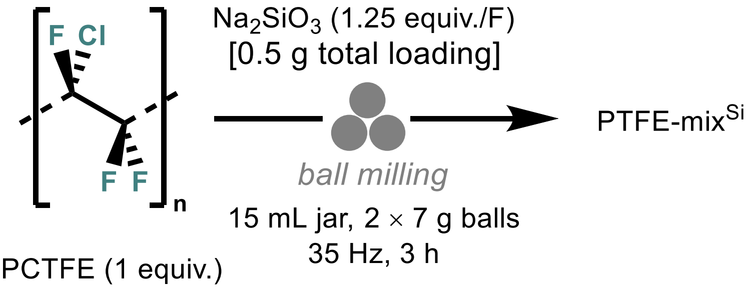

3, PCTFE (1 equiv.)

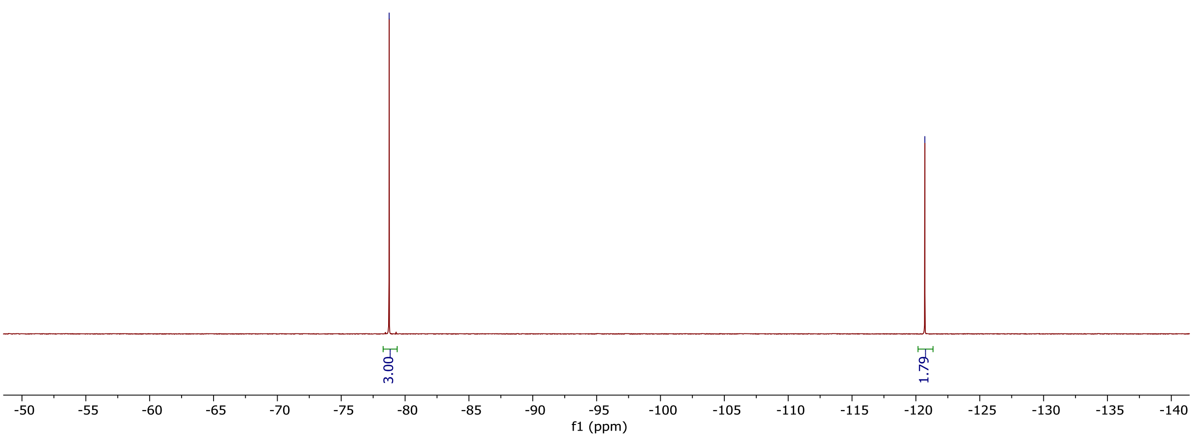

n(OTf⁻)	m (sample)	Yield of F⁻ %
0.058 mmol	31 mg	94%

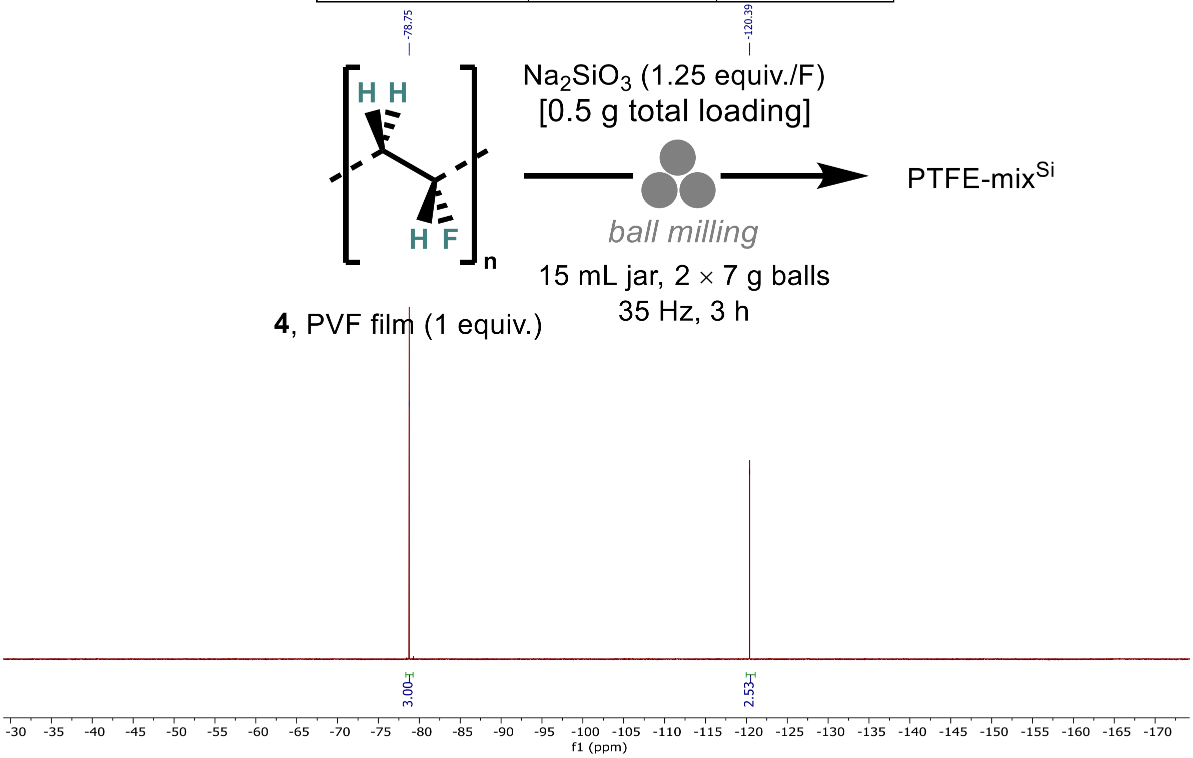

n(OTf⁻)	m (sample)	Yield of F⁻ %
0.060 mmol	20 mg	85%

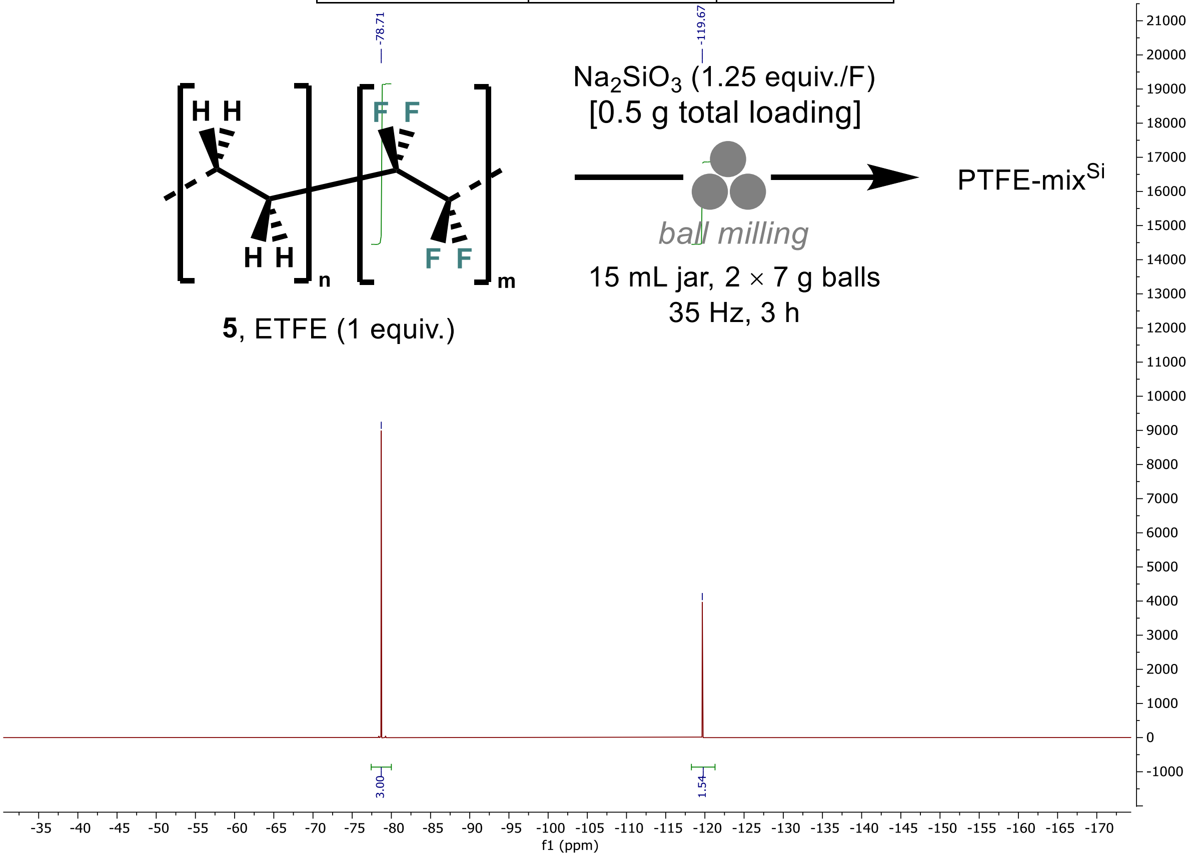

n(OTf⁻)	m (sample)	Yield of F⁻ %
0.067 mmol	23 mg	78%

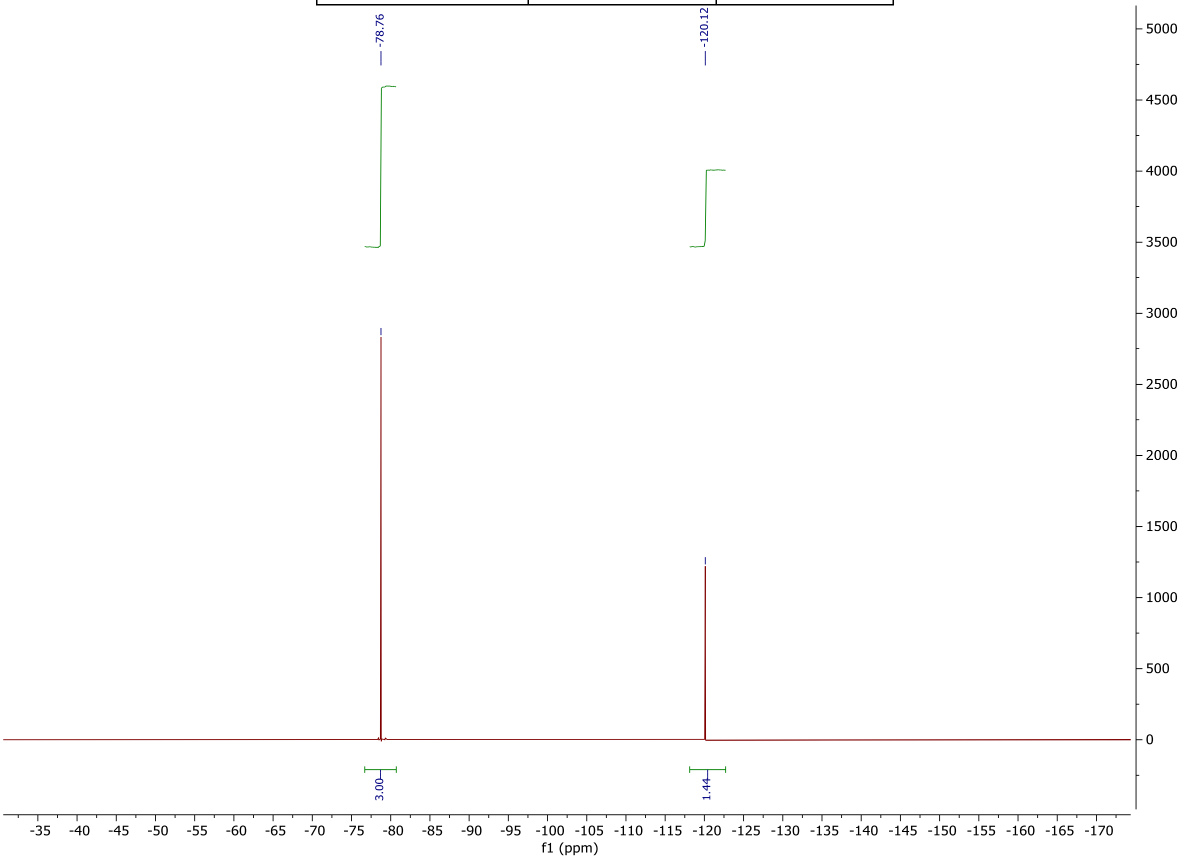

n(OTf⁻)	m (sample)	Yield of F⁻ %
0.13 mmol	26 mg	83%

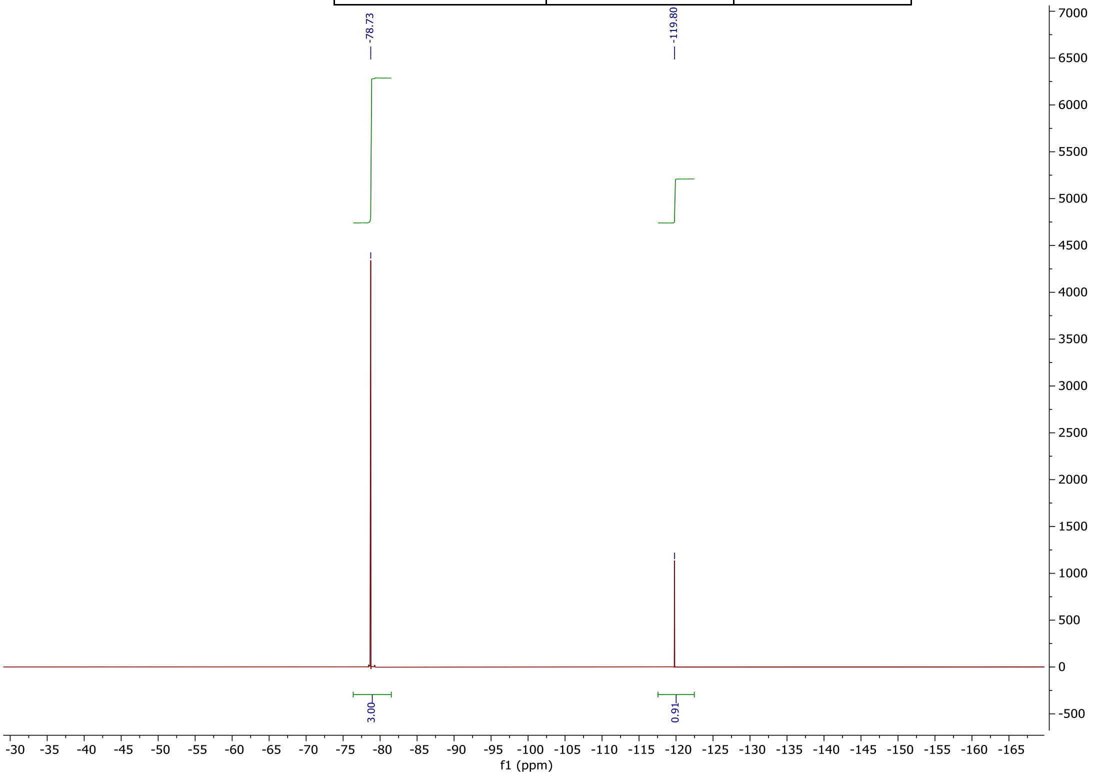

n(OTf⁻)	m (sample)	Yield of F⁻ %
0.084 mmol	23 mg	88%

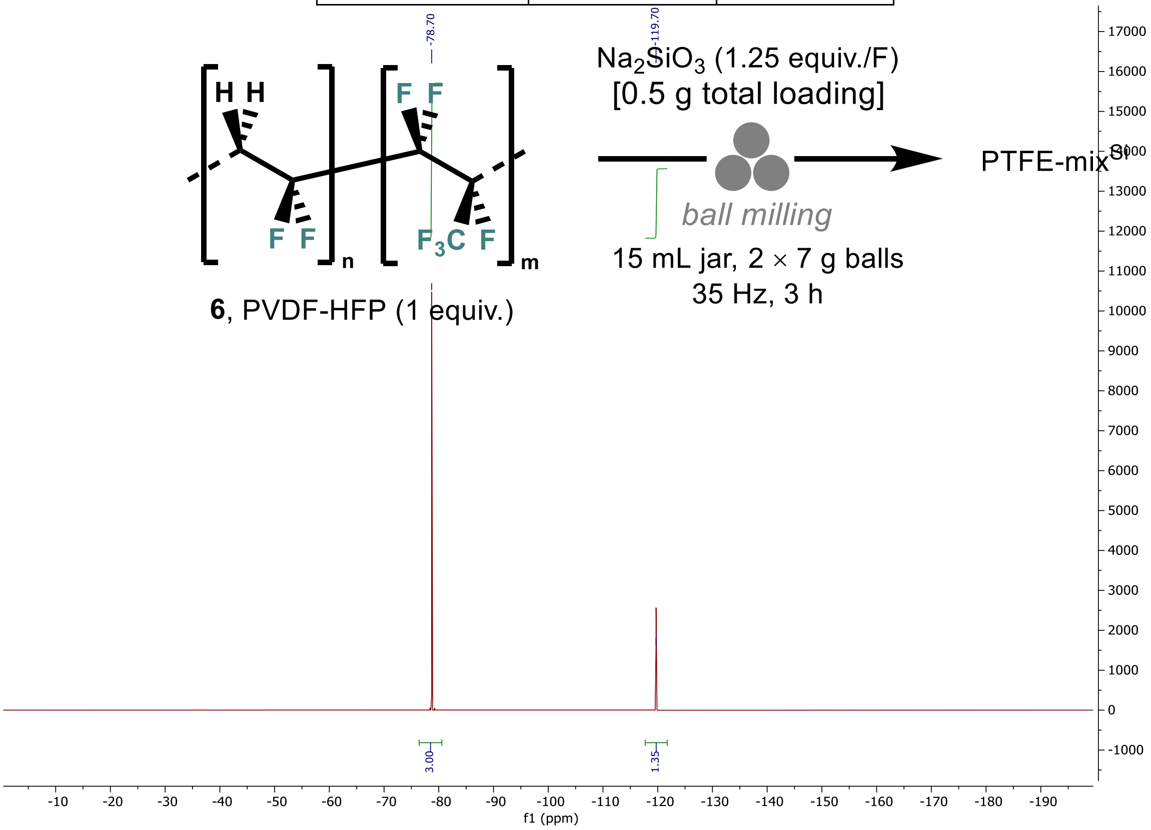

n(OTf⁻)	m (sample)	Yield of F⁻ %
0.060 mmol	22.0 mg	87%

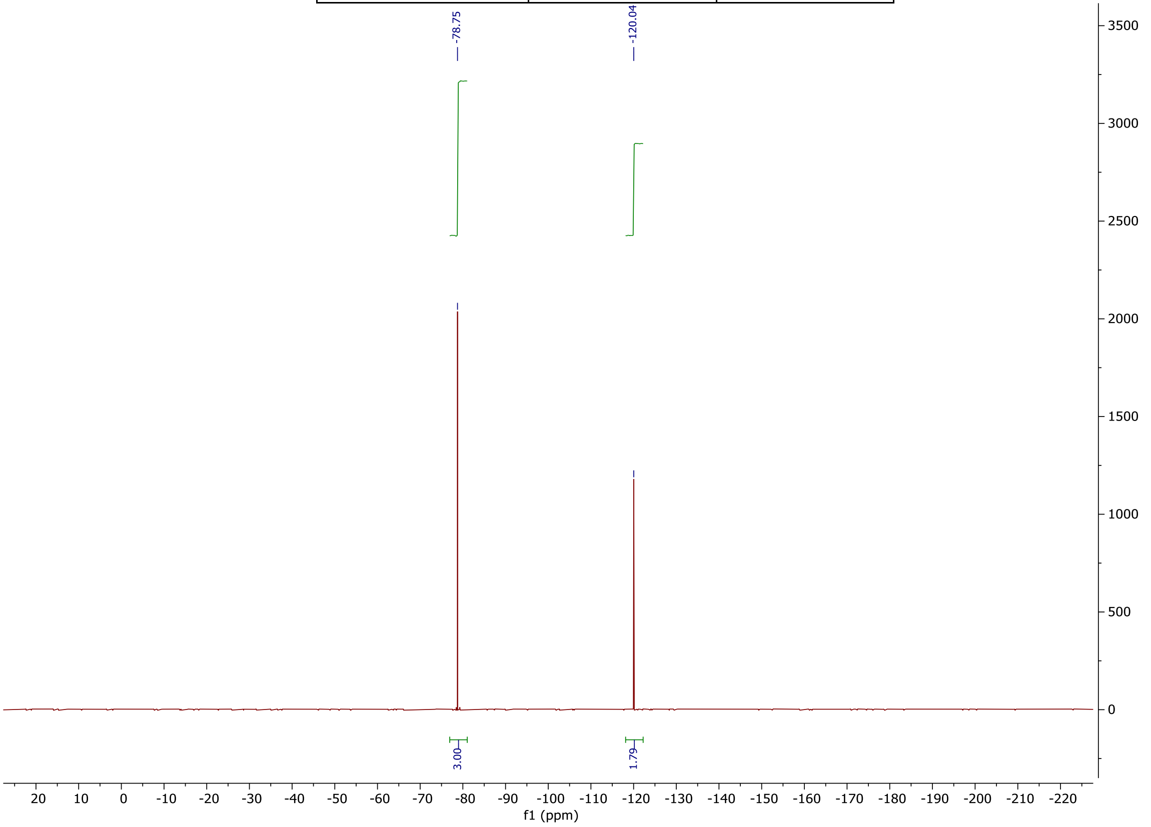

n(OTf⁻)	m (sample)	Yield of F⁻ %
0.058 mmol	25.6 mg	84%

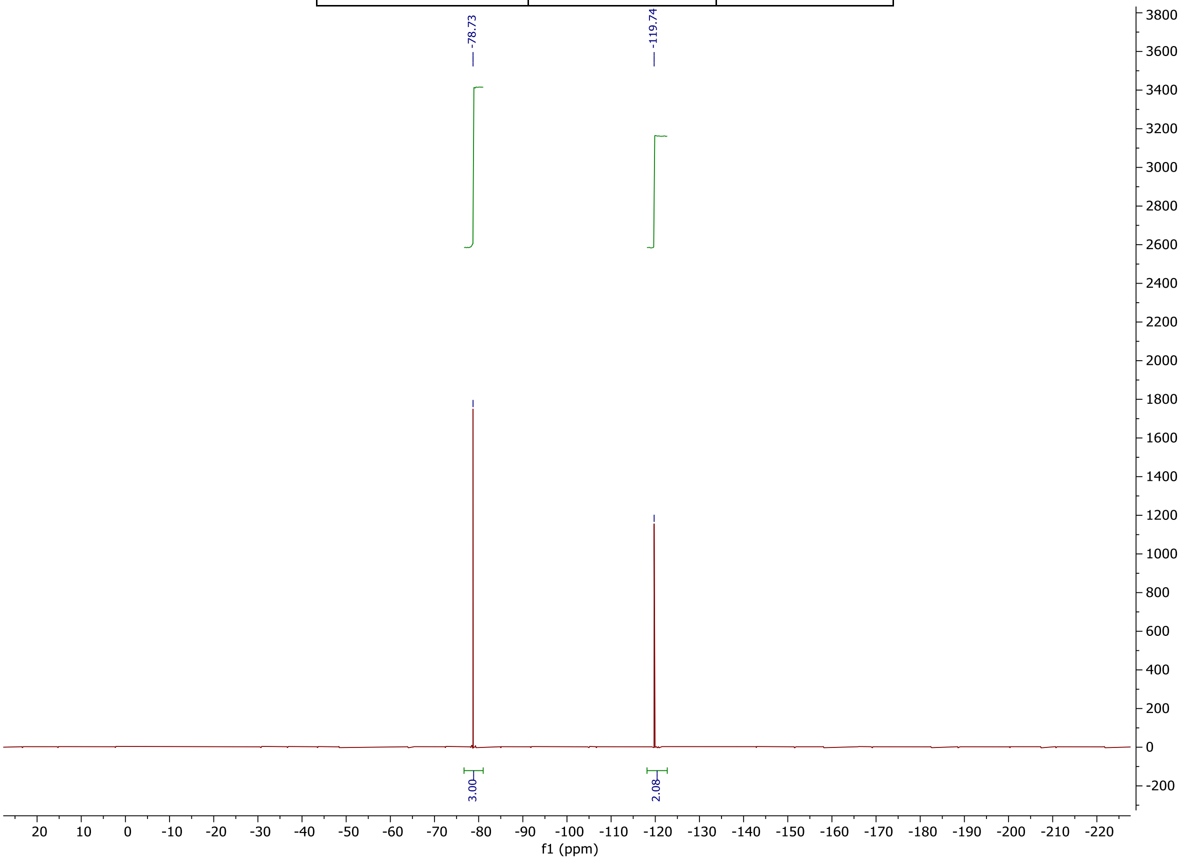

n(OTf⁻)	m (sample)	Yield of F⁻ %
0.058 mmol	19.5 mg	quant.

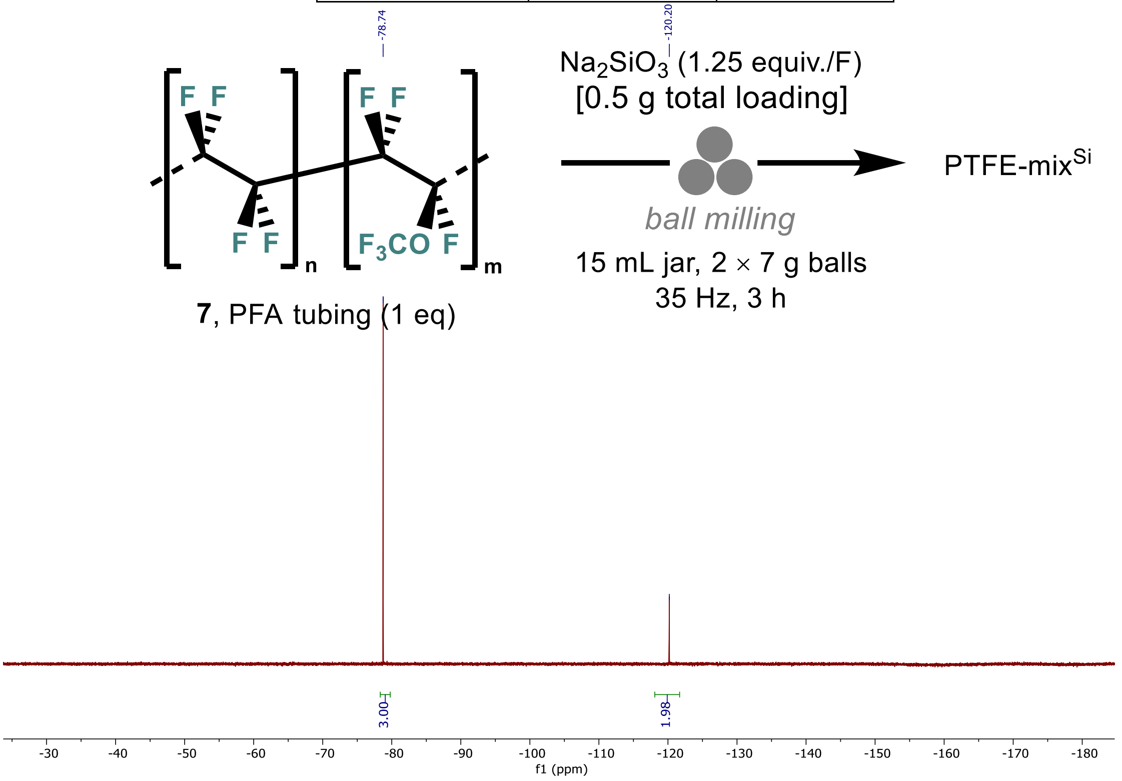

n(OTf⁻)	m (sample)	Yield of F⁻ %
0.069 mmol	21 mg	88%

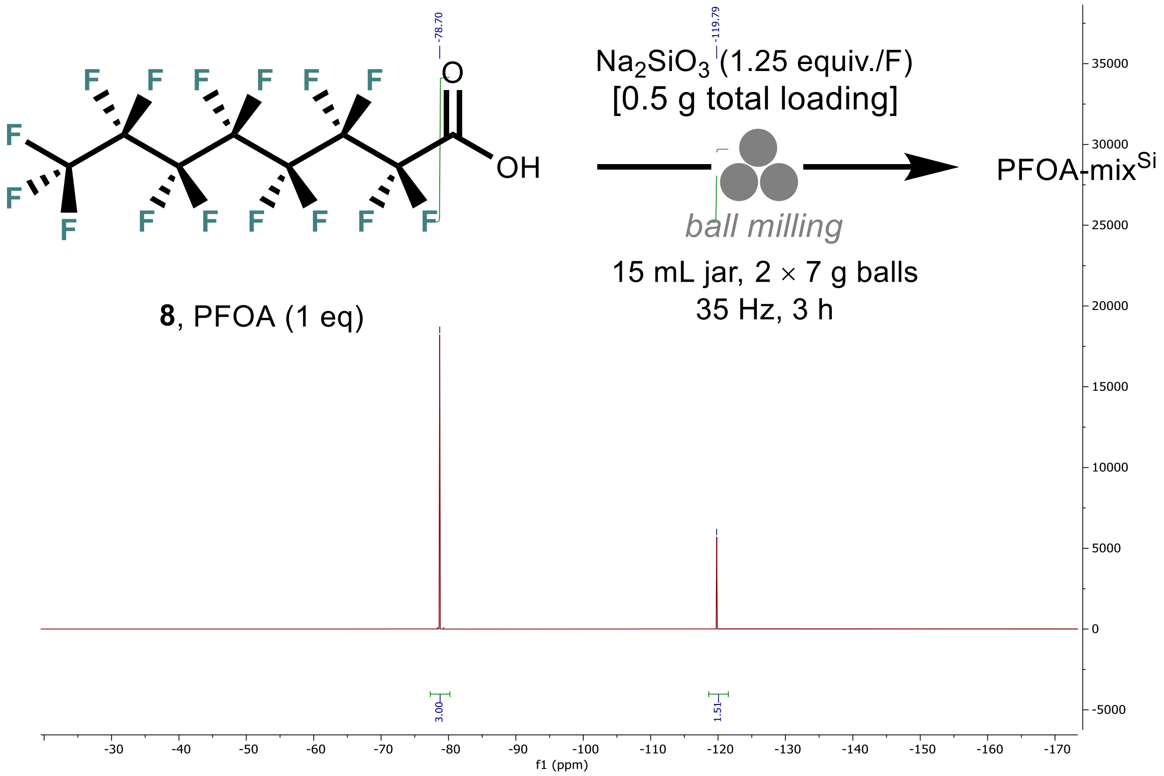

n(OTf⁻)	m (sample)	Yield of F⁻ %
0.037 mmol	25 mg	83%

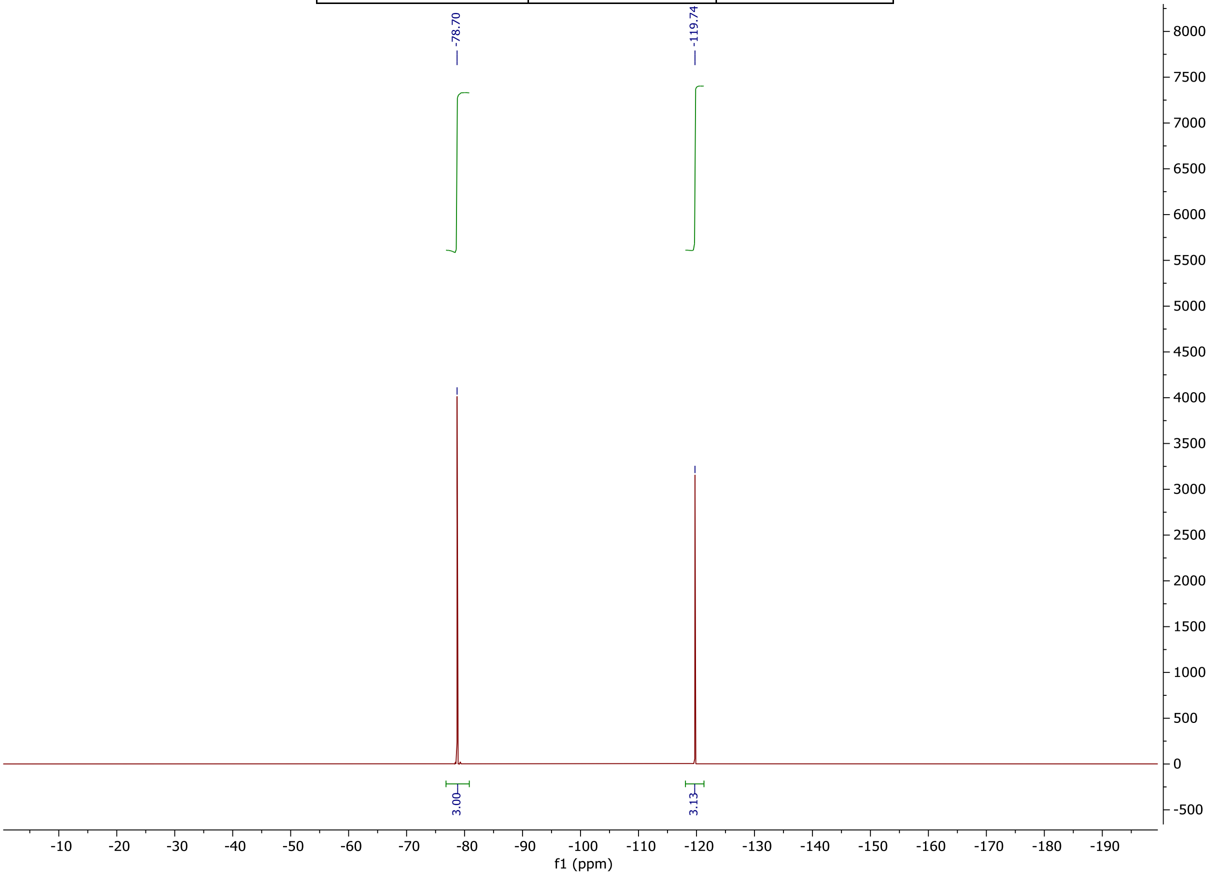

n(OTf⁻)	m (sample)	Yield of F⁻ %
0.089 mmol	28 mg	91%

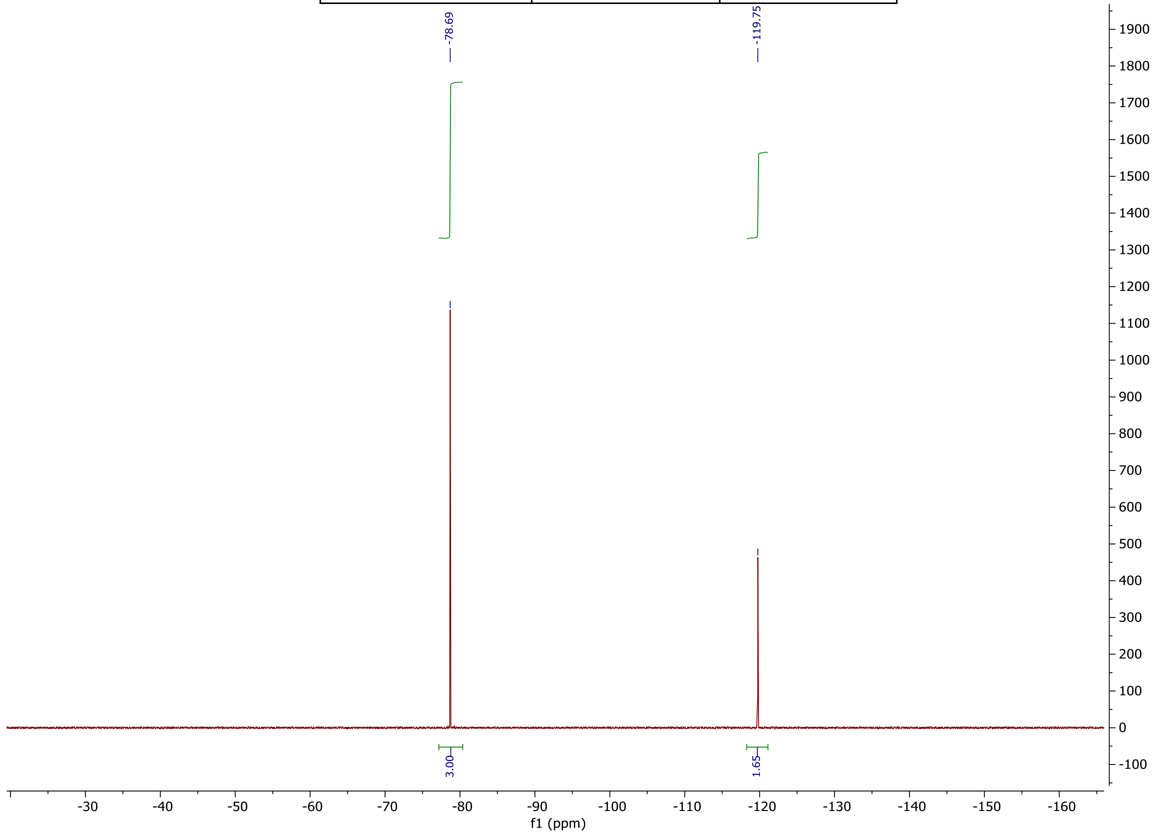

n(OTf⁻)	m (sample)	Yield of F⁻ %
0.069 mmol	27 mg	80%

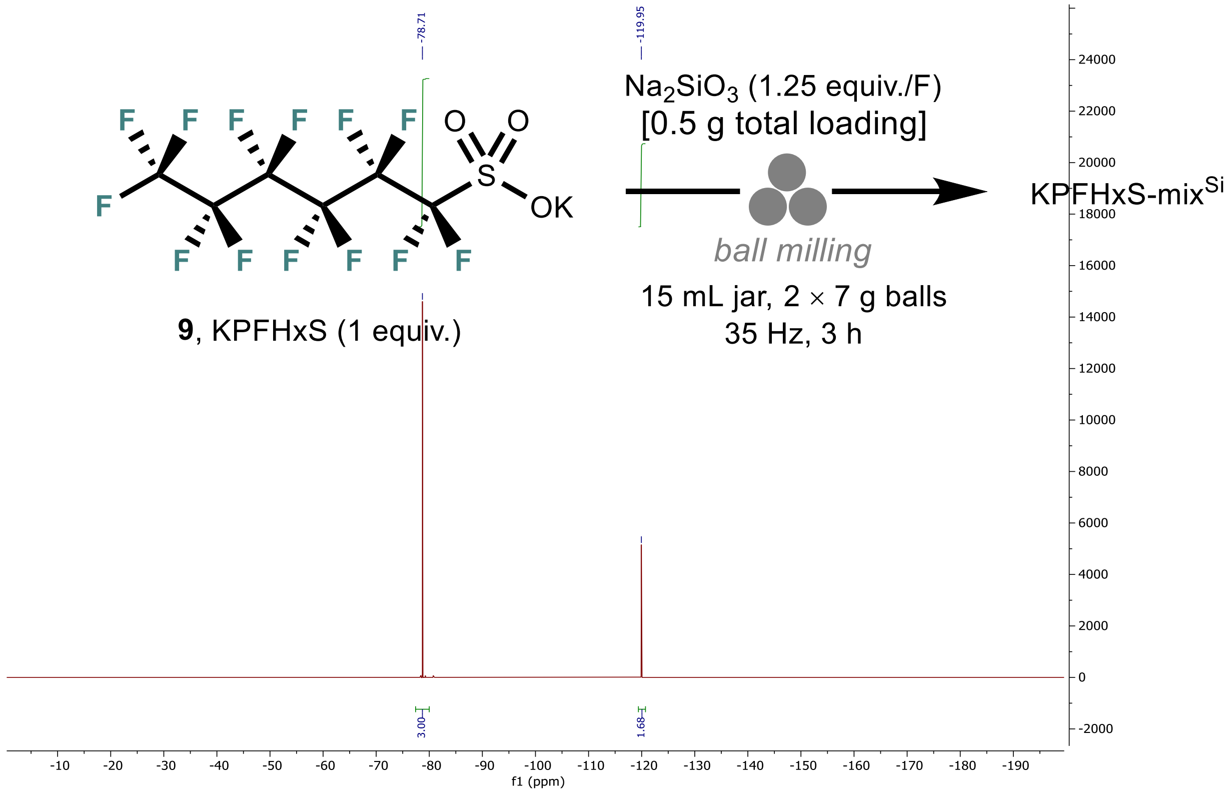

n(OTf⁻)	m (sample)	Yield of F⁻ %
0.056 mmol	21 mg	74%

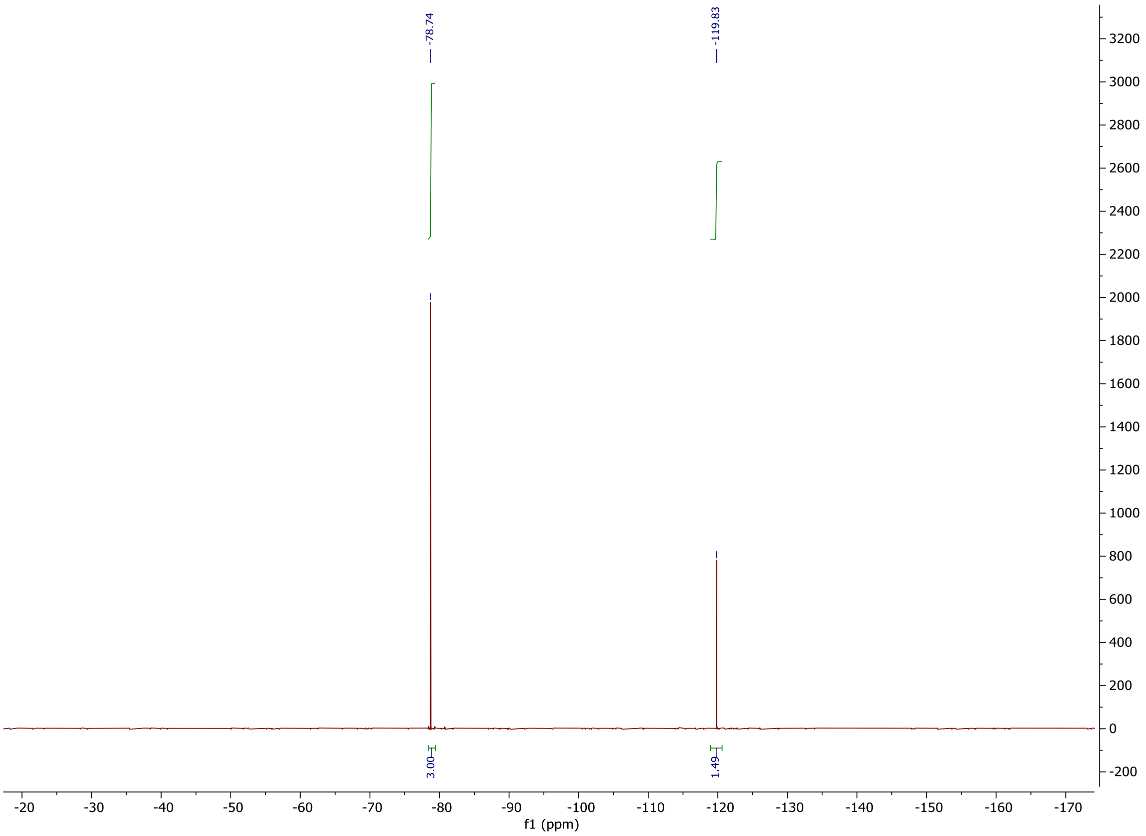

n(OTf⁻)	m (sample)	Yield of F⁻ %
0.085 mmol	22.6 mg	88%

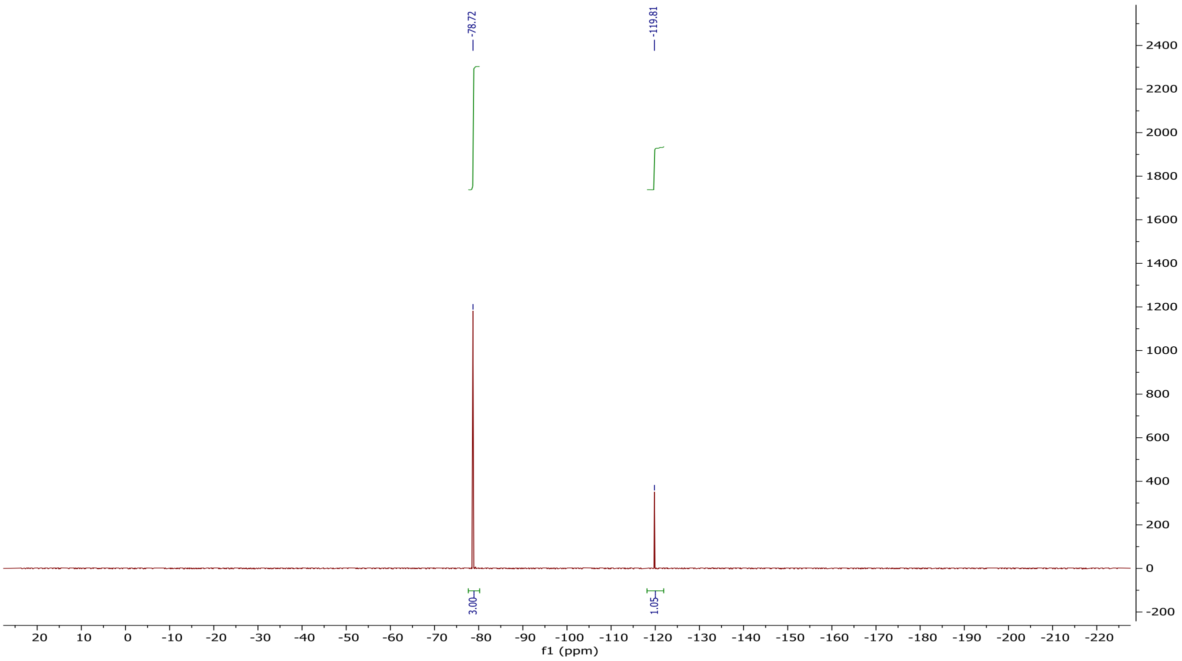

n(OTf⁻)	m (sample)	Yield of F⁻ %
0.058 mmol	38 mg	quant.

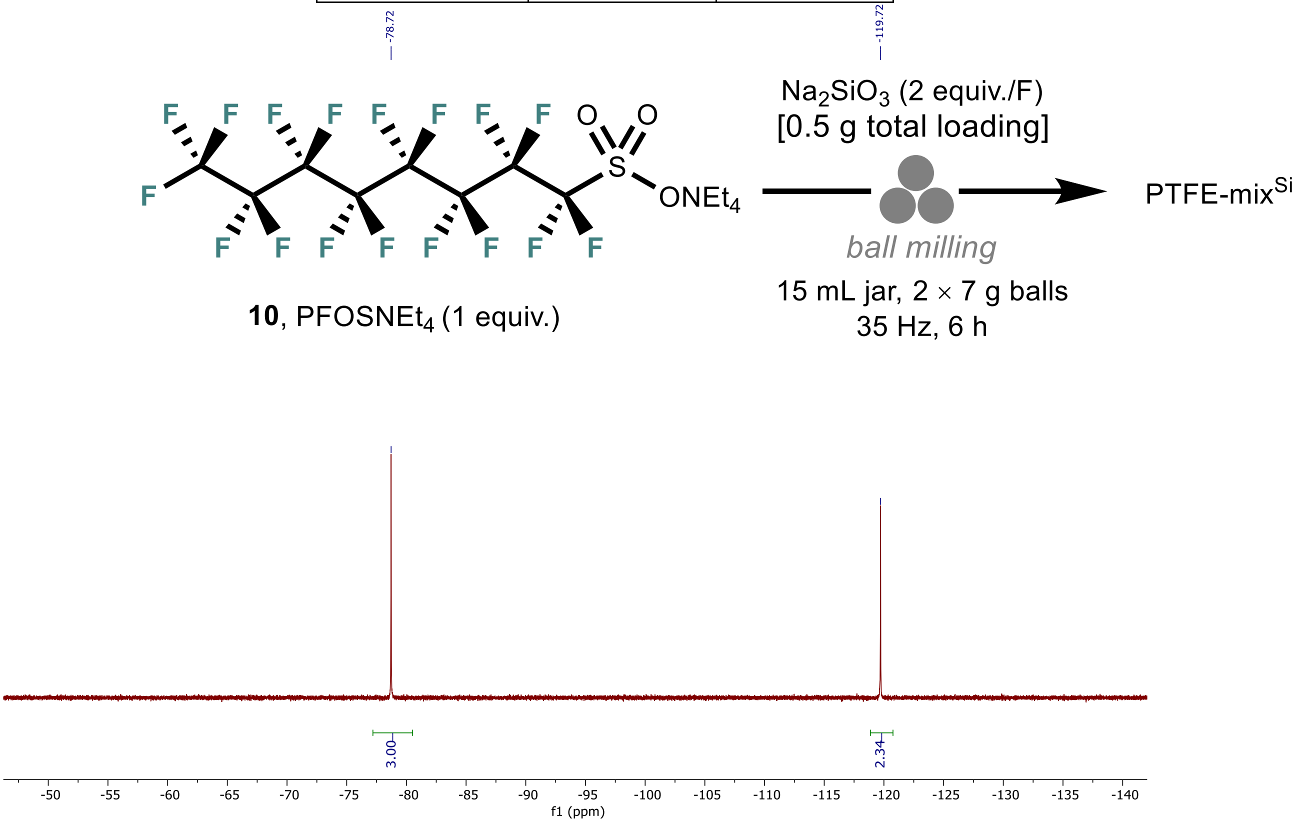

n(OTf⁻)	m (sample)	Yield of F⁻ %
0.053 mmol	26.1 mg	quant.

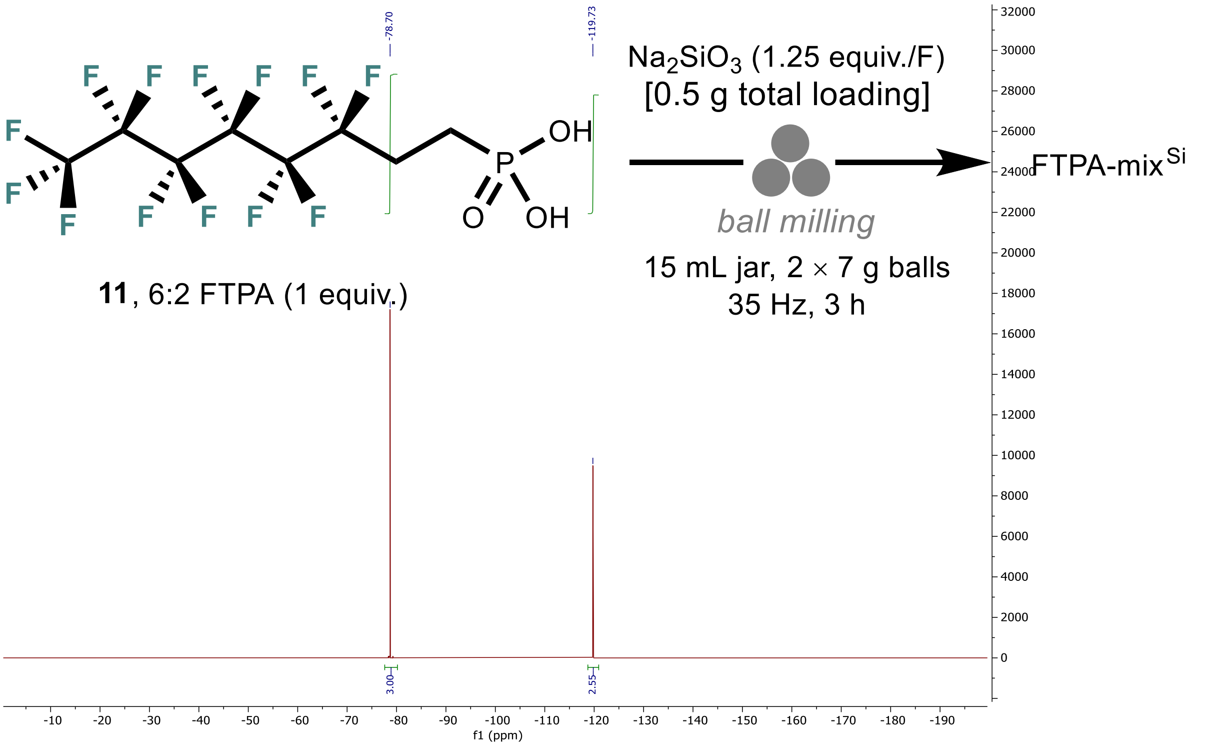

n(OTf⁻)	m (sample)	Yield of F⁻ %
0.19 mmol	21 mg	95%

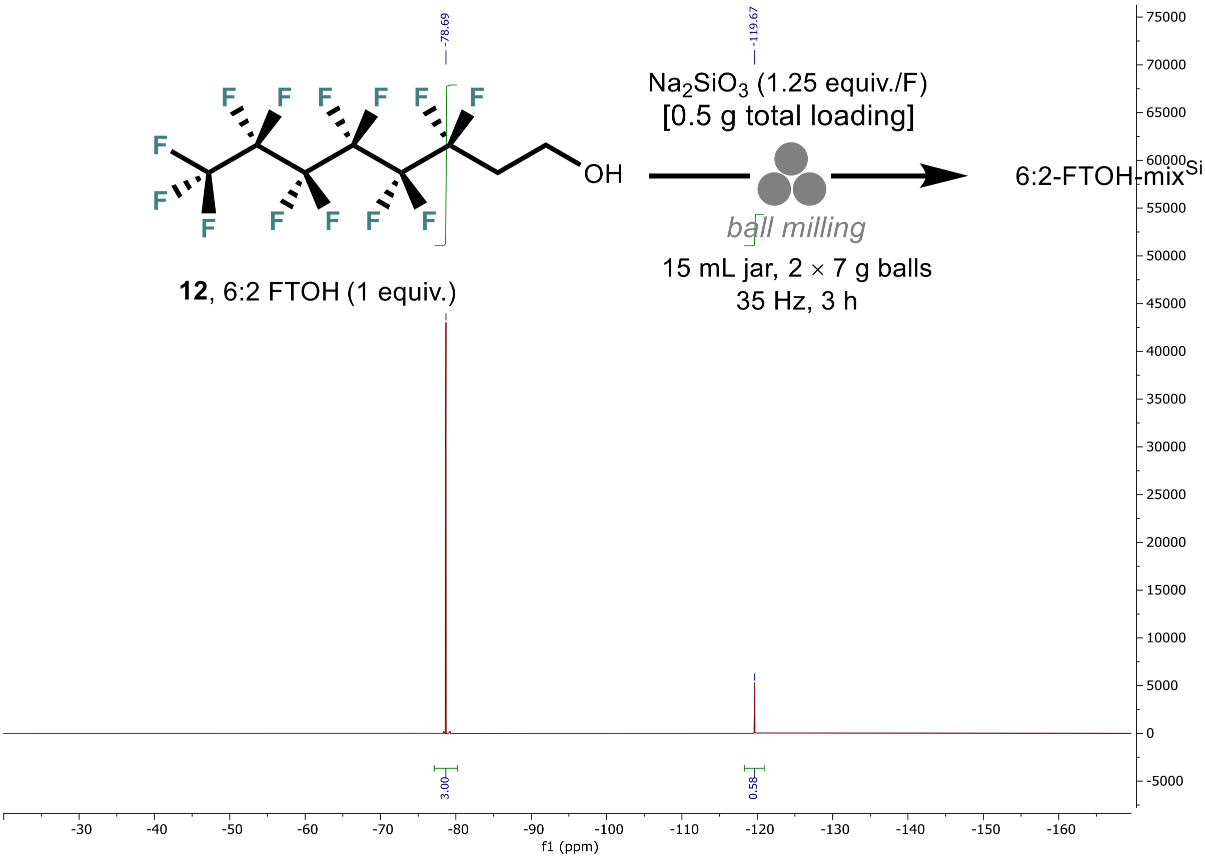

n(OTf⁻)	m (sample)	Yield of F⁻ %
0.042 mmol	29 mg	99%

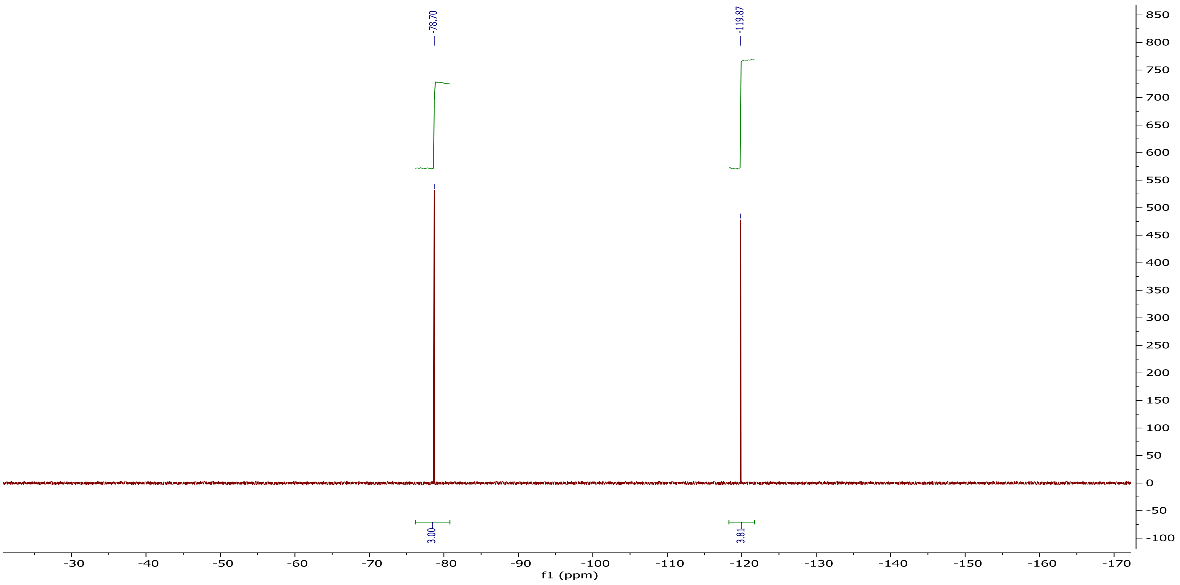

n(OTf⁻)	m (sample)	Yield of F⁻ %
0.099 mmol	23 mg	100%

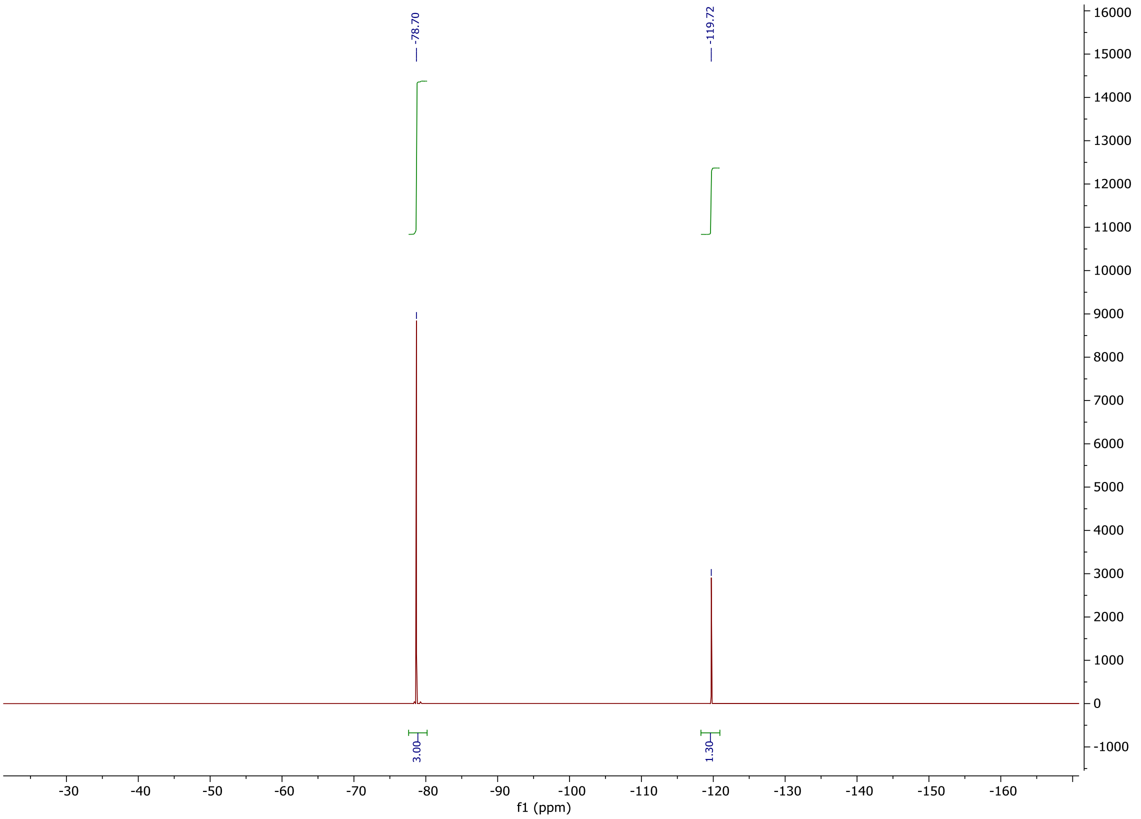

n(OTf⁻)	m (sample)	Yield of F⁻ %
0.13 mmol	22 mg	quant.

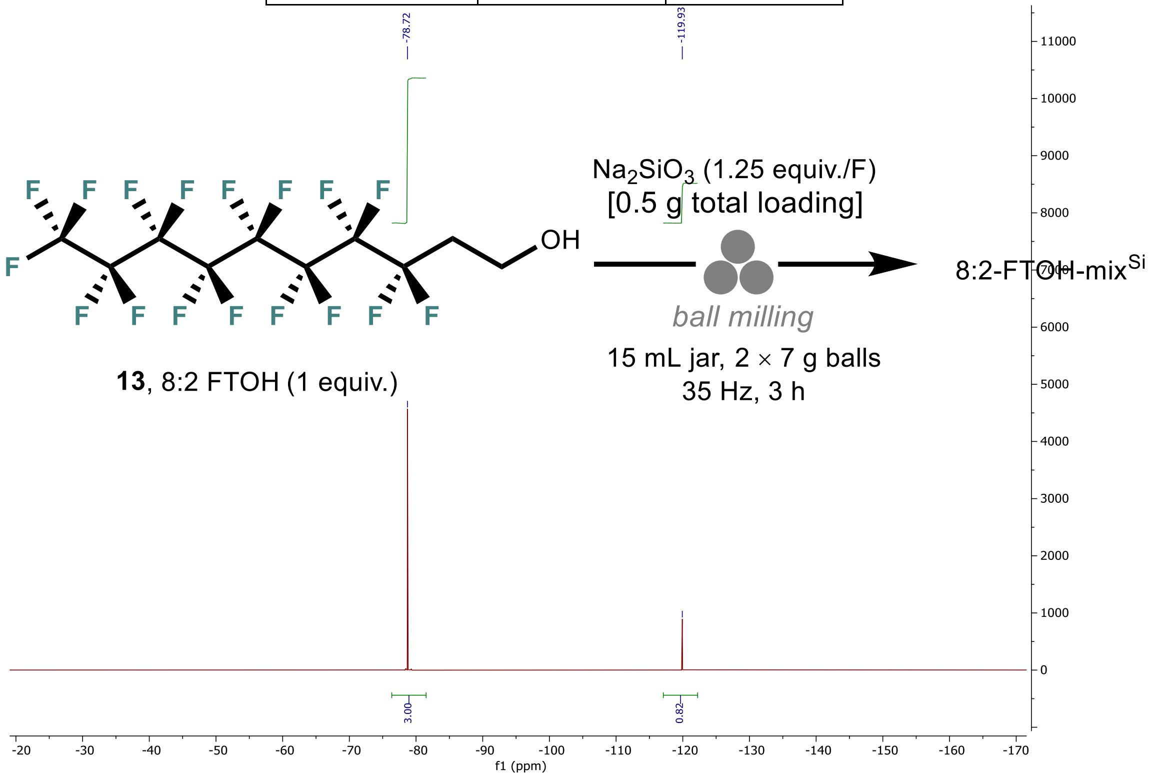

n(OTf⁻)	m (sample)	Yield of F⁻ %
0.099 mmol	25 mg	quant.

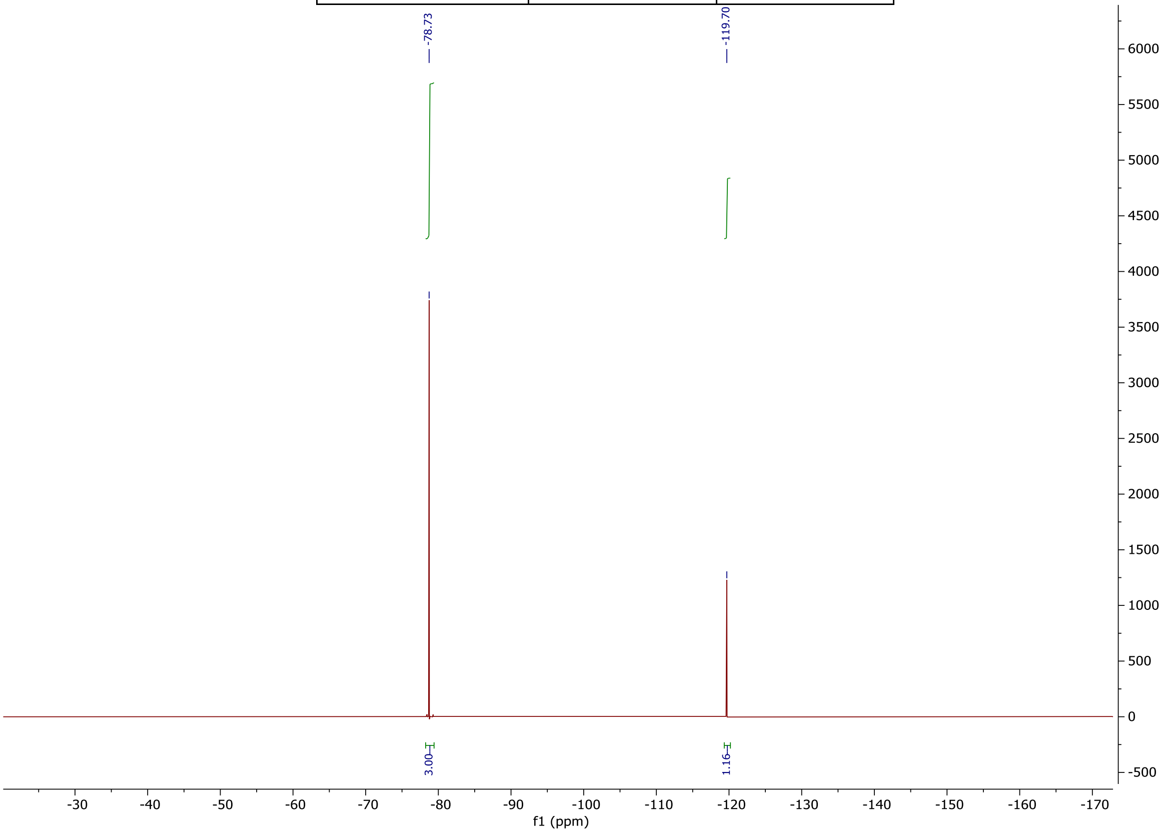

n(OTf⁻)	m (sample)	Yield of F⁻ %
0.062 mmol	20 mg	quant.

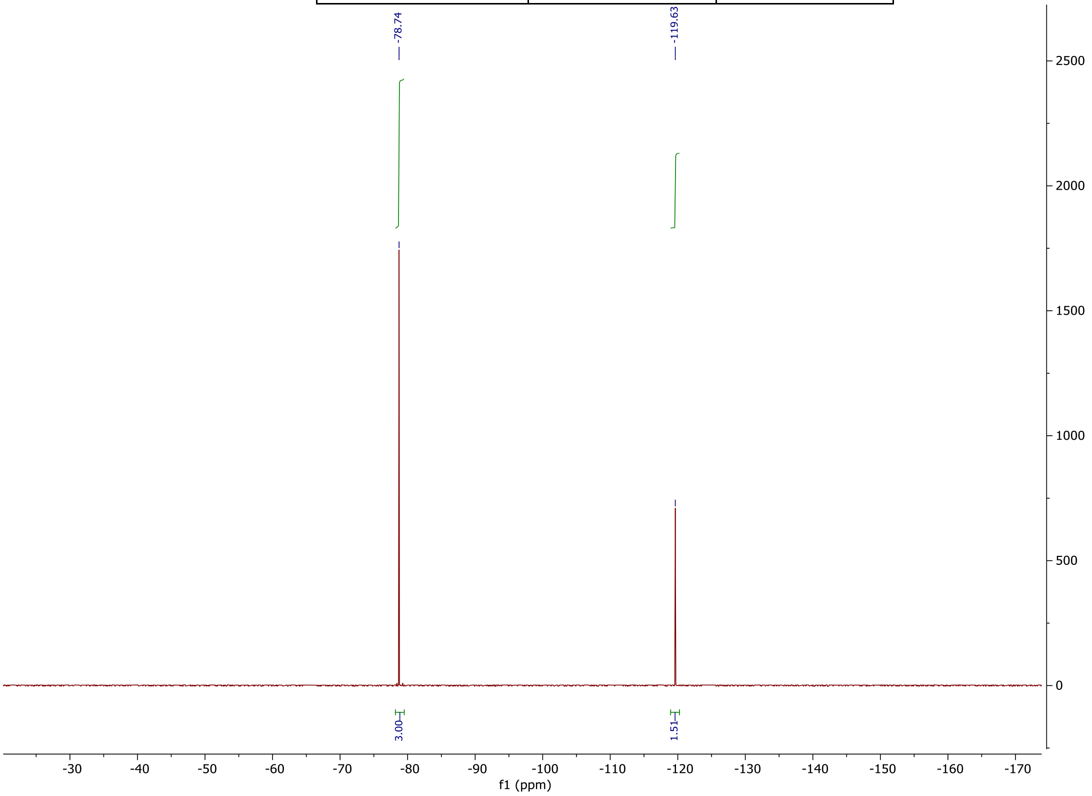

n(OTf⁻)	m (sample)	Yield of F⁻ %
0.044 mmol	35 mg	99%

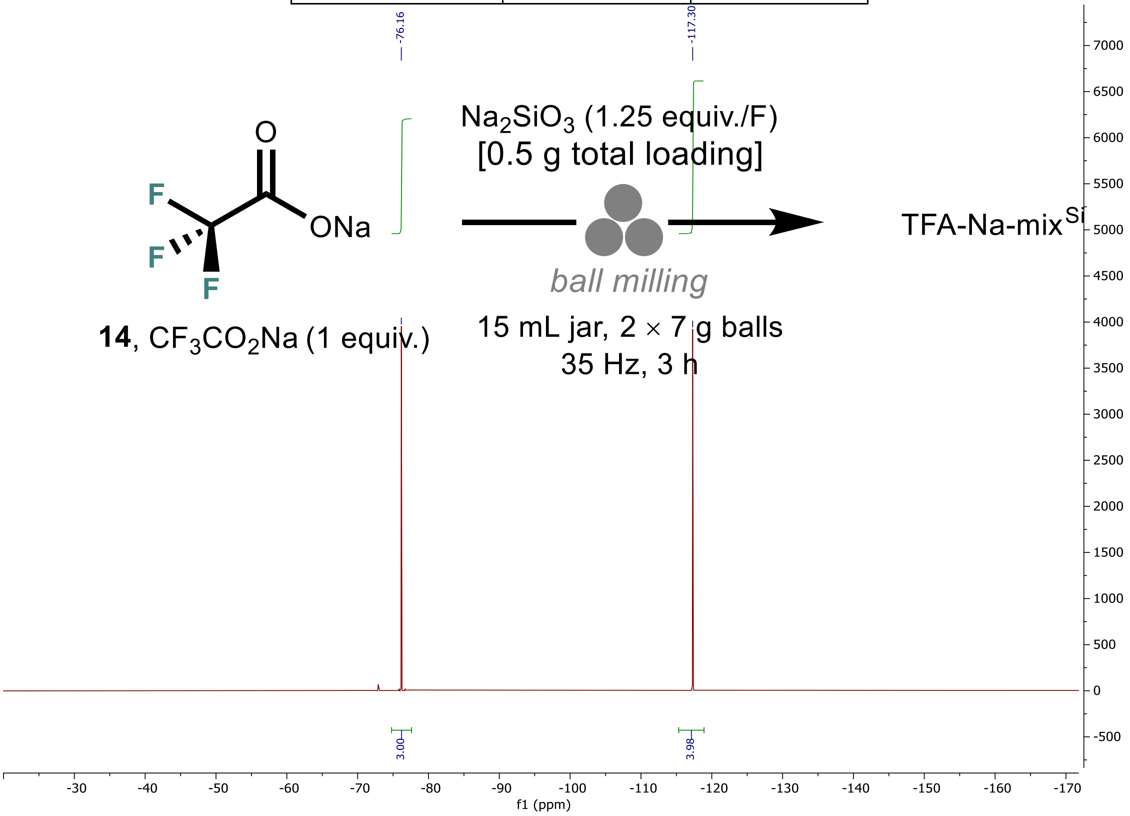

n(OTf⁻)	m (sample)	Yield of F⁻ %
0.092 mmol	28 mg	quant.

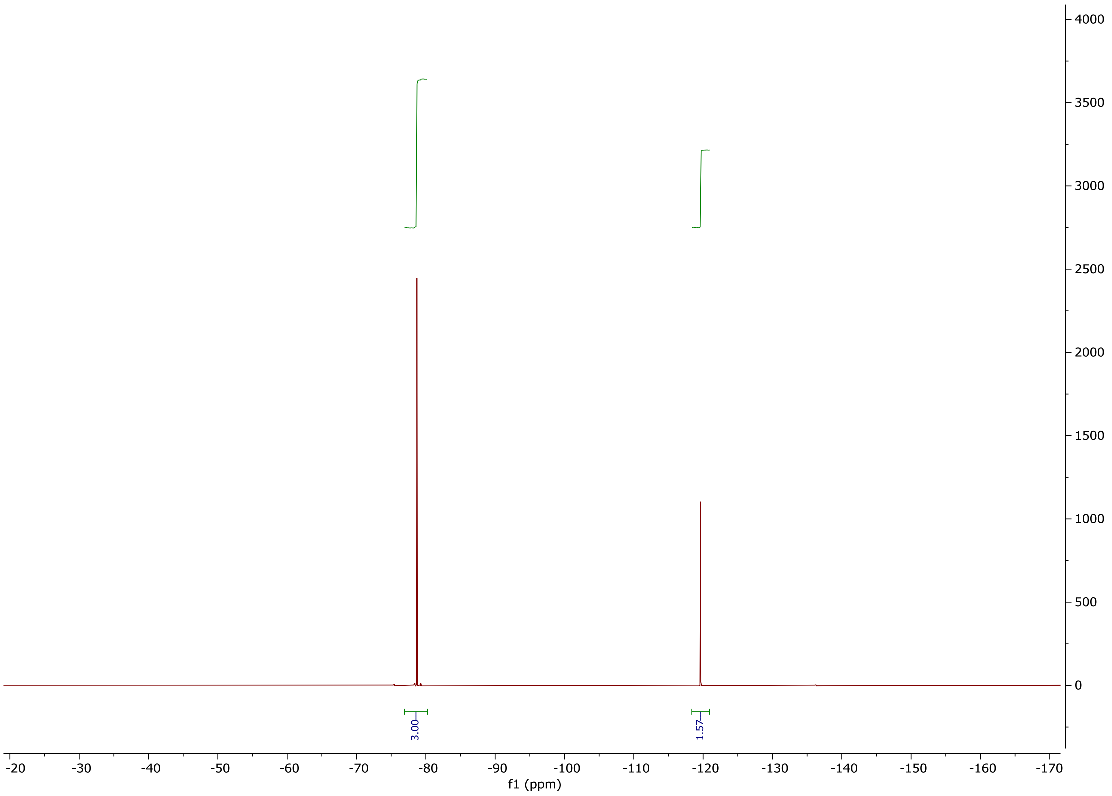

n(OTf⁻)	m (sample)	Yield of F⁻ %
0.070 mmol	21 mg	99%

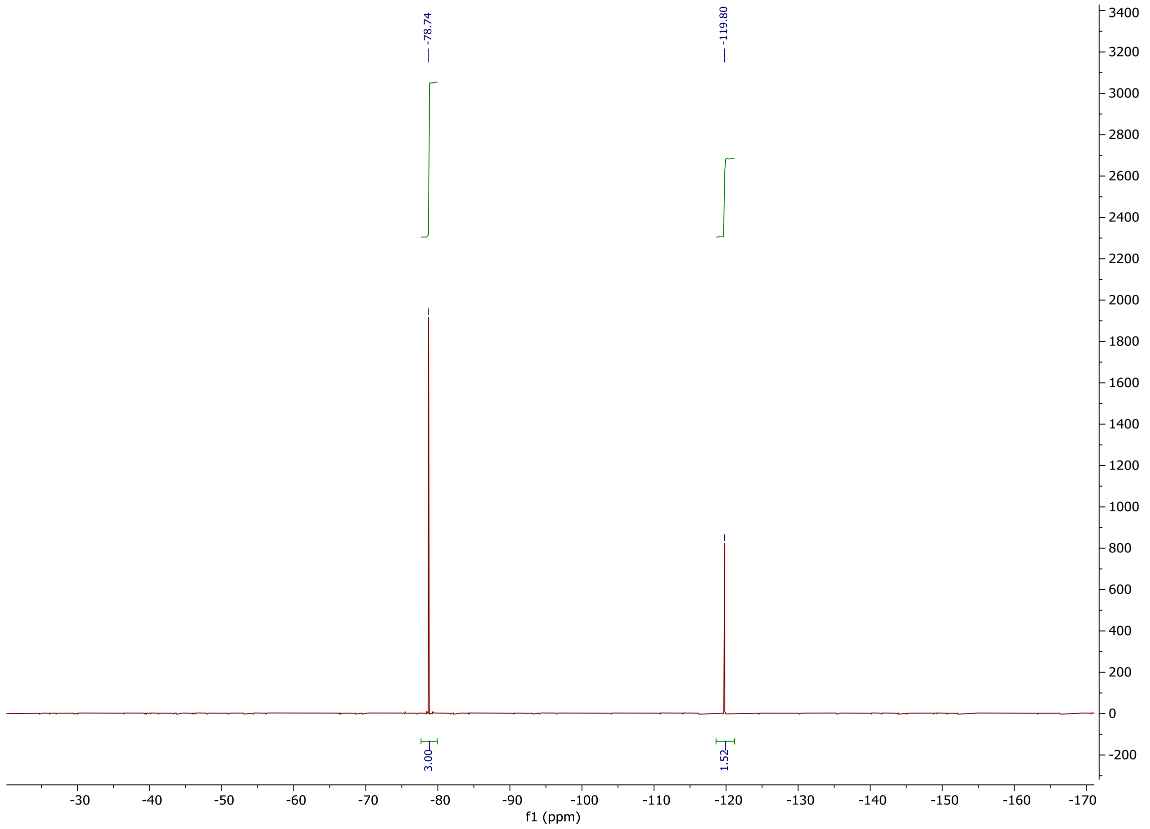

n(OTf⁻)	m (sample)	Yield of F⁻ %
0.058 mmol	17 mg	quant.

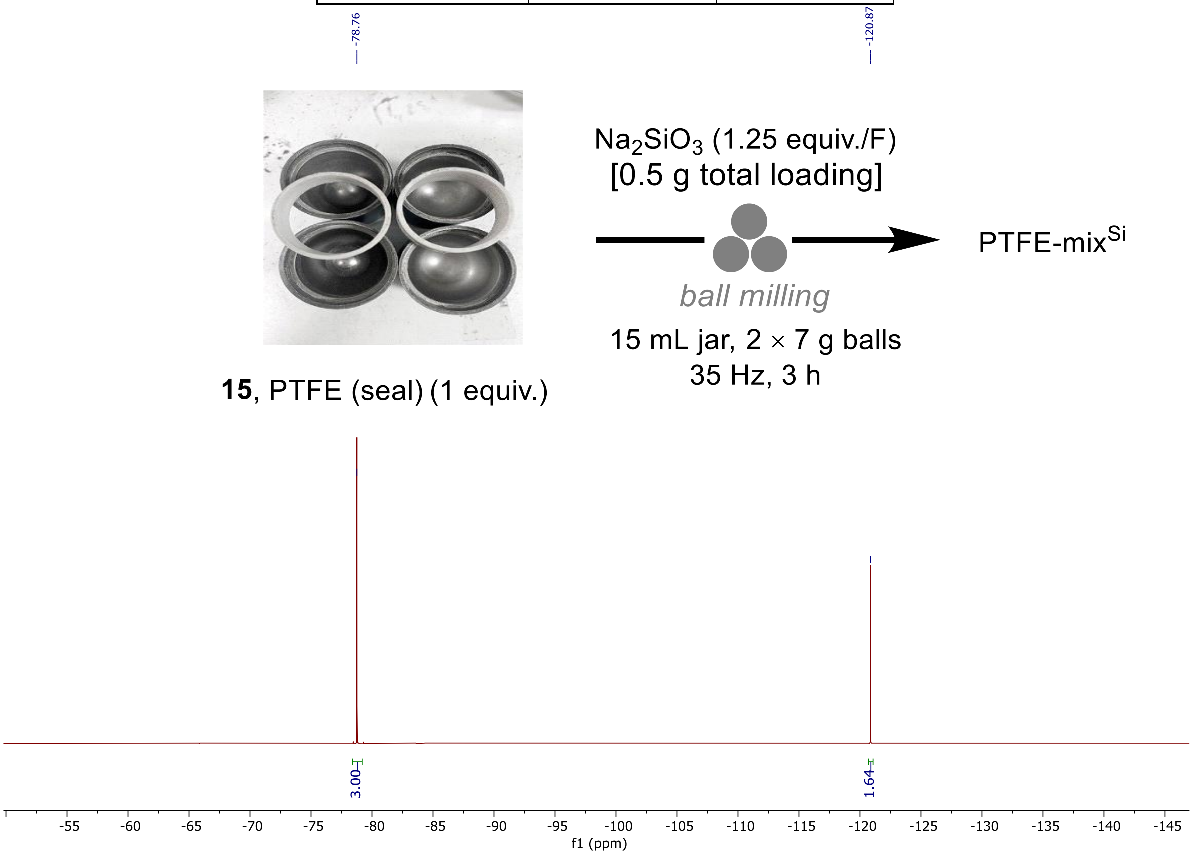

15, PTFE (seal) (1 equiv.)

n(OTf⁻)	m (sample)	Yield of F⁻ %
0.058 mmol	13.5 mg	quant.

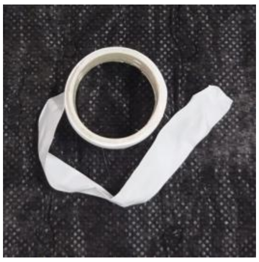

16, PTFE (tape) (1 equiv.)

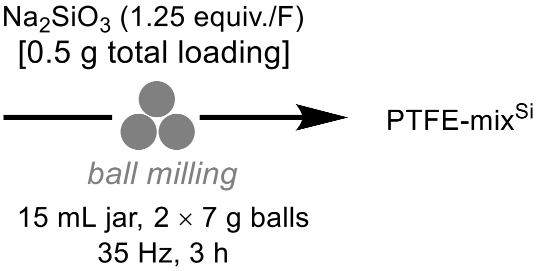

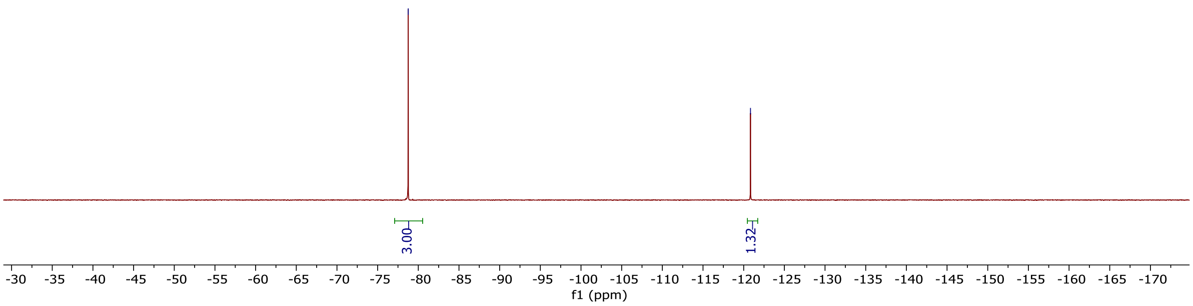

n(OTf⁻)	m (sample)	Yield of F⁻ %
0.058 mmol	20.5 mg	quant.

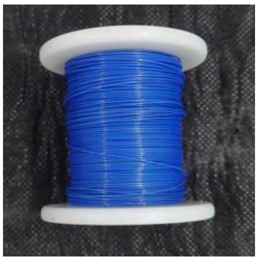

17, ETFE wire (1 equiv.)

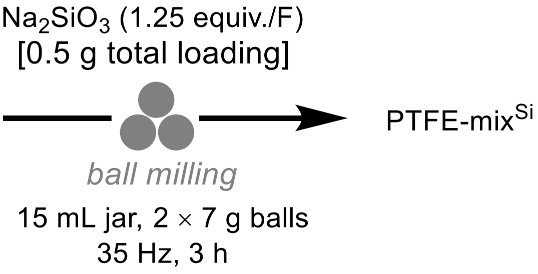

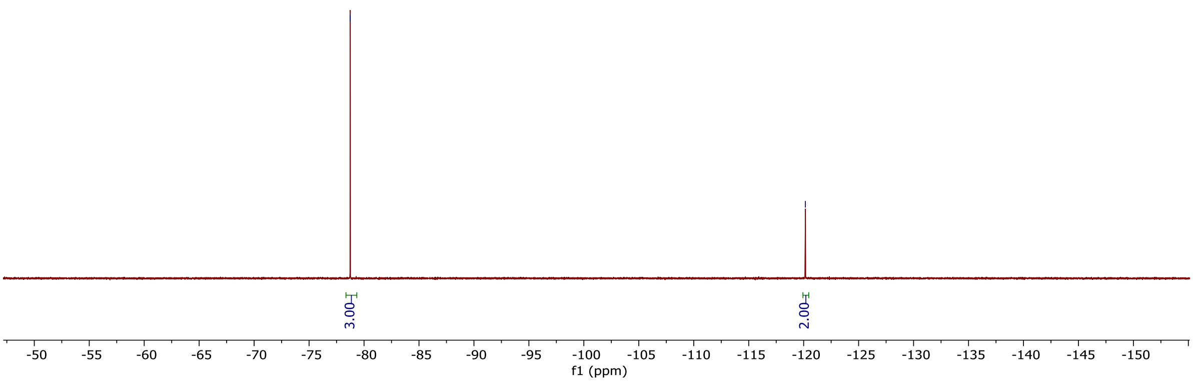

n(OTf⁻)	m (sample)	Yield of F⁻ %
0.058 mmol	12.4 mg	quant.

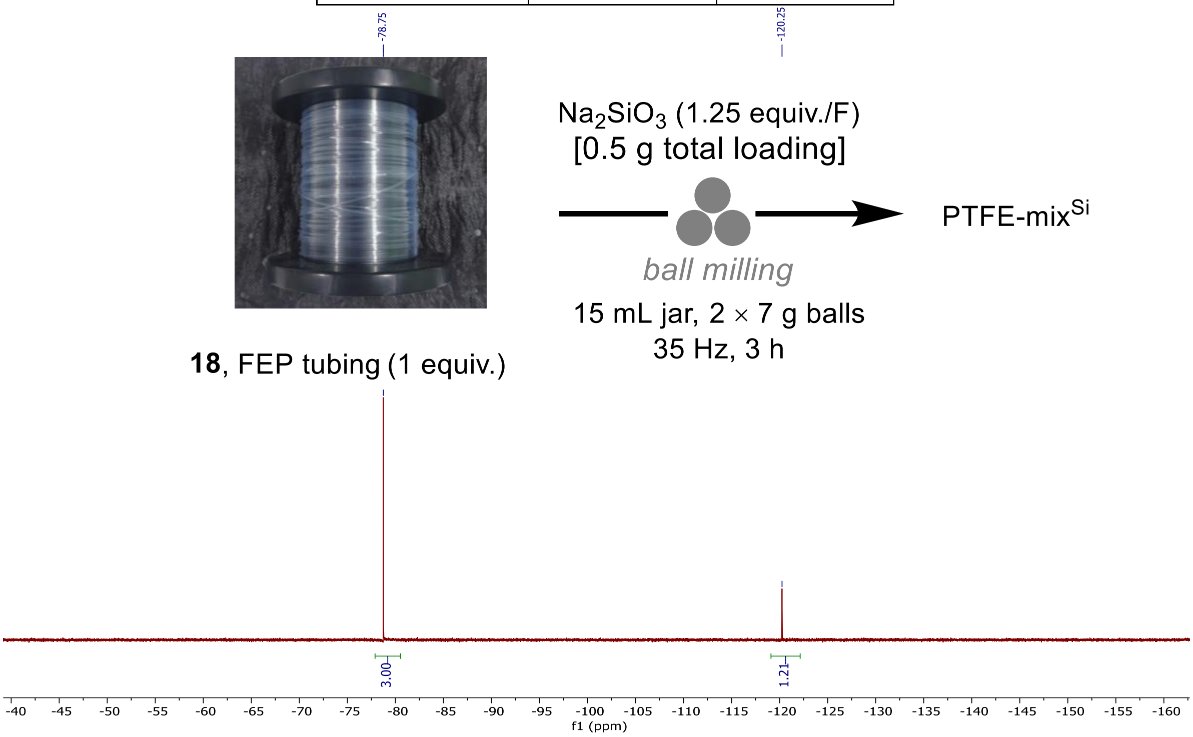

n(OTf⁻)	m (sample)	Yield of F⁻ %
0.058 mmol	49 mg	quant.

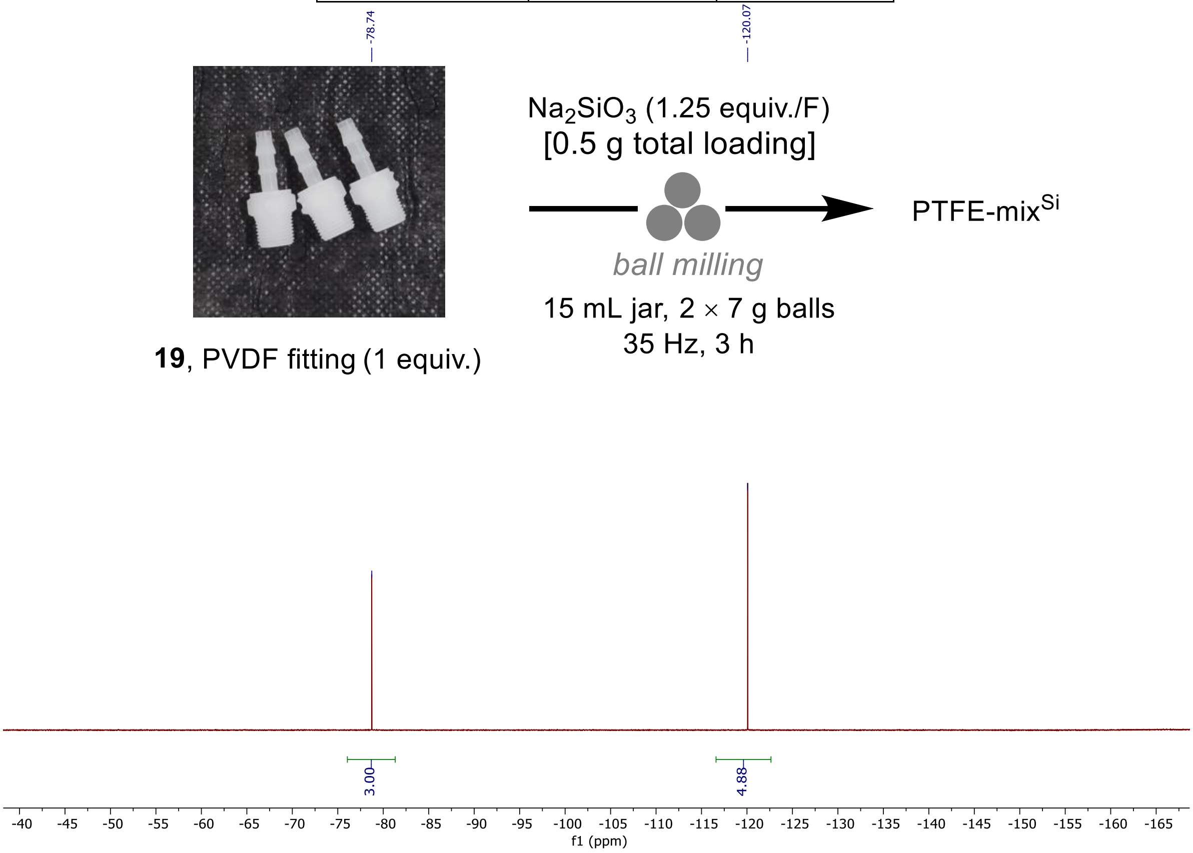

n(OTf⁻)	m (sample)	Yield of F⁻ %
0.058 mmol	25.0 mg	quant.

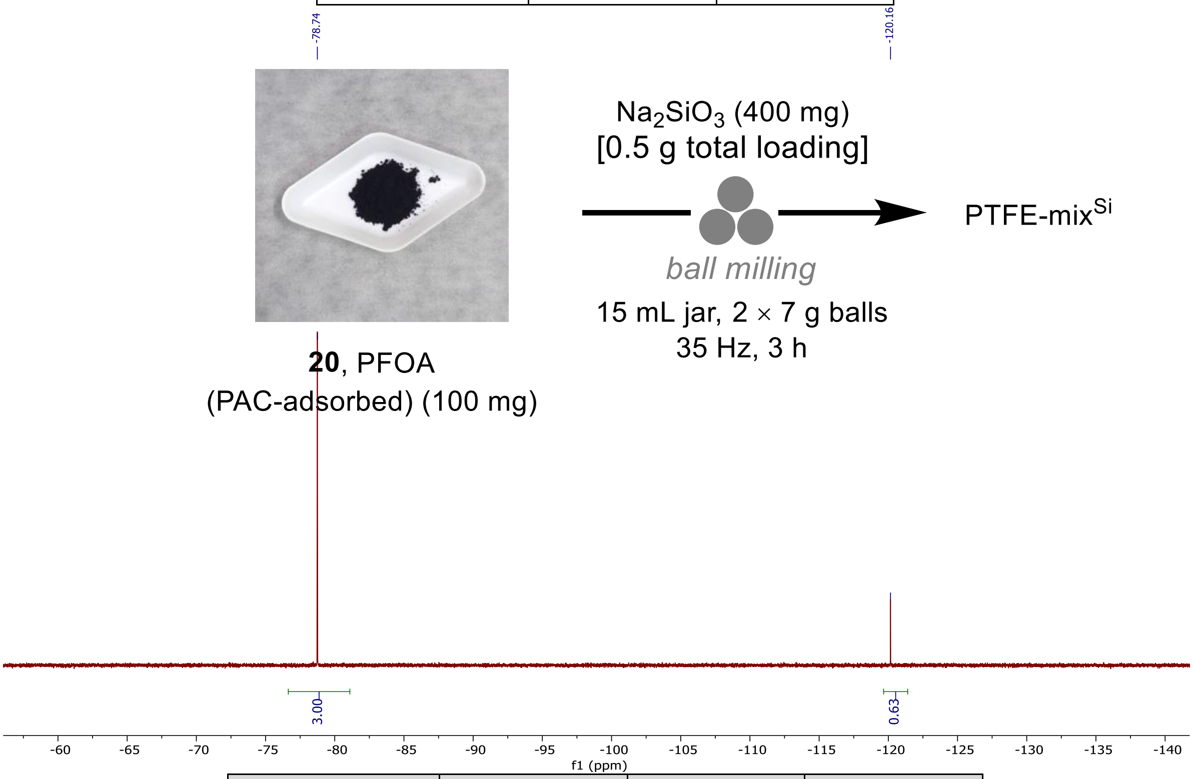

n(OTf⁻)	m (sample)	Water content (mol%)	Yield of F⁻ %
0.19 mmol	500 mg	0.5 mol%	90%

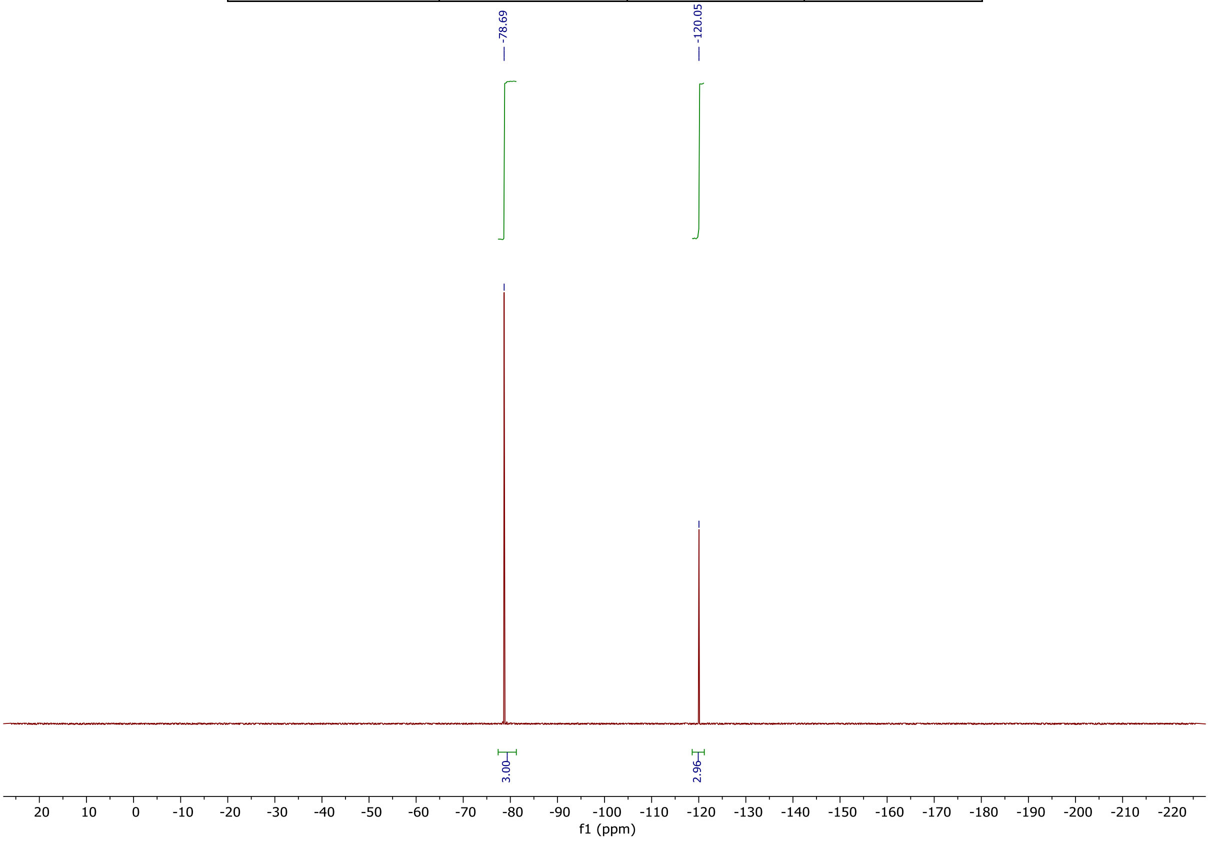

n(OTf⁻)	m (sample)	Water content (mol%)	Yield of F⁻ %
0.25 mmol	500 mg	5 mol%	81%

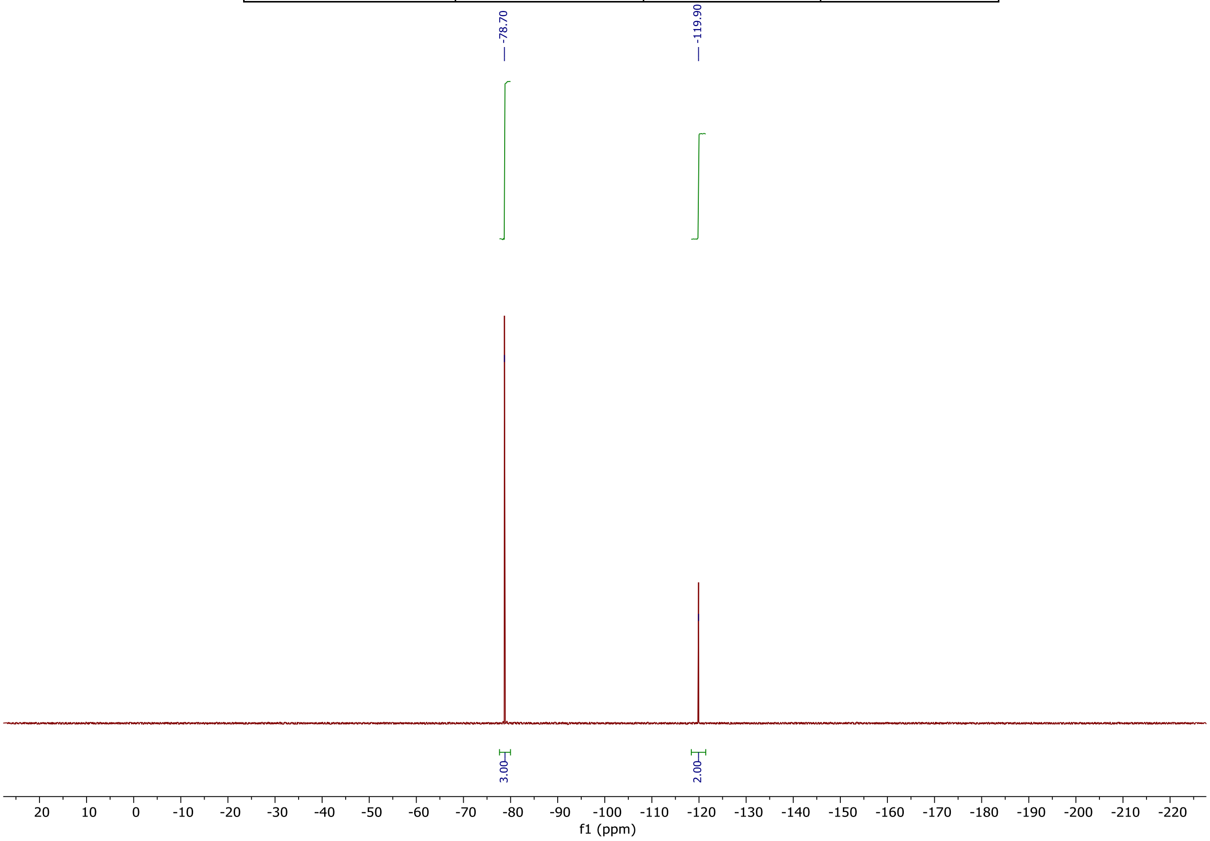

n(OTf⁻)	m (sample)	Water content (mol%)	Yield of F⁻ %
0.25 mmol	500 mg	10 mol%	83%

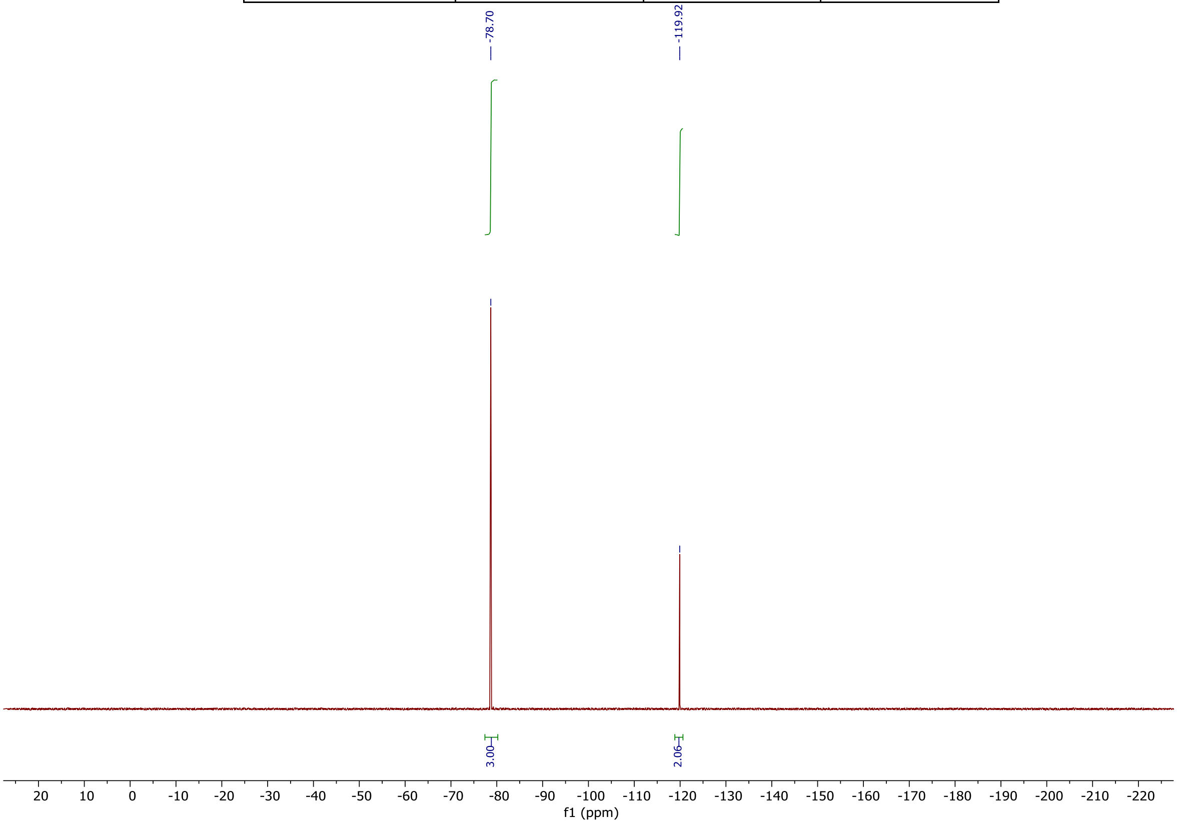

n(OTf⁻)	m (sample)	Yield of F⁻ %
0.058 mmol	33 mg	quant.

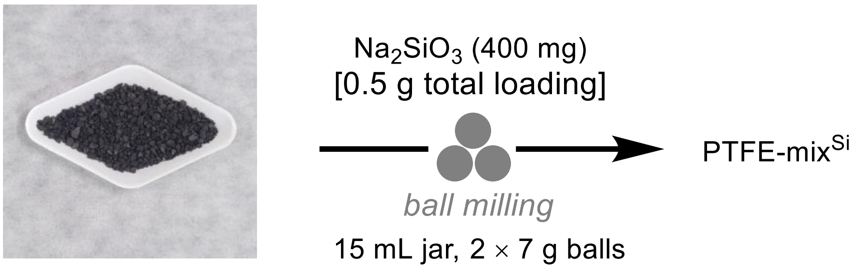

21, 8:2 FTOH
(GAC-adsorbed) (100 mg)

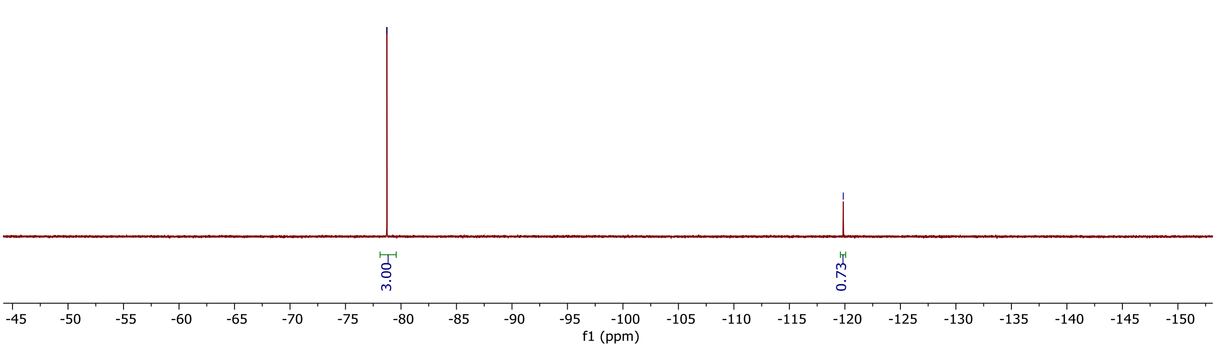

Supplement: Supplementary file 1 [file ja6c01470_si_001.pdf]
